# Early prolonged prone position in noninvasively ventilated patients with SARS-CoV-2-related moderate-to-severe hypoxemic respiratory failure: clinical outcomes and mechanisms for treatment response in the PRO-NIV study

**DOI:** 10.1186/s13054-022-03937-x

**Published:** 2022-04-29

**Authors:** Giovanni Musso, Claudio Taliano, Federica Molinaro, Caterina Fonti, Deliana Veliaj, Davide Torti, Elena Paschetta, Elisabetta Castagna, Giorgio Carbone, Luigi Laudari, Claudio Aseglio, Edoardo Zocca, Sonia Chioni, Laura Ceretto Giannone, Federica Arabia, Cecilia Deiana, Francesca Maria Benato, Marta Druetta, Giorgio Campagnola, Margherita Borsari, Martina Mucci, Tiziana Rubatto, Mara Peyronel, Gloria Tirabassi

**Affiliations:** 1 Emergency Medicine Department, HUMANITAS Gradenigo, C.so Regina Margherita 8, 10132 Turin, Italy; 2 Intensive Care Unit, HUMANITAS Gradenigo, Turin, Italy; 3 Radiology Department, HUMANITAS Gradenigo, Turin, Italy

**Keywords:** Noninvasive ventilation, Ventilatory ratio, Corrected minute ventilation, Lung ultrasound, Dead space

## Abstract

**Background:**

Whether prone position (PP) improves clinical outcomes in COVID-19 pneumonia treated with noninvasive ventilation (NIV) is unknown. We evaluated the effect of early PP on 28-day NIV failure, intubation and death in noninvasively ventilated patients with moderate-to-severe acute hypoxemic respiratory failure due to COVID-19 pneumonia and explored physiological mechanisms underlying treatment response.

**Methods:**

In this controlled non-randomized trial, 81 consecutive prospectively enrolled patients with COVID-19 pneumonia and moderate-to-severe (paO2/FiO2 ratio < 200) acute hypoxemic respiratory failure treated with early PP + NIV during Dec 2020–May 2021were compared with 162 consecutive patients with COVID-19 pneumonia matched for age, mortality risk, severity of illness and paO2/FiO2 ratio at admission, treated with conventional (supine) NIV during Apr 2020–Dec 2020 at HUMANITAS Gradenigo Subintensive Care Unit, after propensity score adjustment for multiple baseline and treatment-related variables to limit confounding. Lung ultrasonography (LUS) was performed at baseline and at day 5. Ventilatory parameters, physiological dead space indices (DSIs) and circulating inflammatory and procoagulative biomarkers were monitored during the initial 7 days.

**Results:**

In the intention-to-treat analysis. NIV failure occurred in 14 (17%) of PP patients versus 70 (43%) of controls [HR = 0.32, 95% CI 0.21–0.50; *p* < 0.0001]; intubation in 8 (11%) of PP patients versus 44 (30%) of controls [HR = 0.31, 95% CI 0.18–0.55; *p* = 0.0012], death in 10 (12%) of PP patients versus 59 (36%) of controls [HR = 0.27, 95% CI 0.17–0.44; *p* < 0.0001]. The effect remained significant within different categories of severity of hypoxemia (paO2/FiO2 < 100 or paO2/FiO2 100–199 at admission). Adverse events were rare and evenly distributed. Compared with controls, PP therapy was associated with improved oxygenation and DSIs, reduced global LUS severity indices largely through enhanced reaeration of dorso-lateral lung regions, and an earlier decline in inflammatory markers and D-dimer. In multivariate analysis, day 1 CO2 response outperformed O2 response as a predictor of LUS changes, NIV failure, intubation and death.

**Conclusion:**

Early prolonged PP is safe and is associated with lower NIV failure, intubation and death rates in noninvasively ventilated patients with COVID-19-related moderate-to-severe hypoxemic respiratory failure. Early dead space reduction and reaeration of dorso-lateral lung regions predicted clinical outcomes in our study population.

**Clinical trial registration:**

ISRCTN23016116. Retrospectively registered on May 1, 2021.

**Supplementary Information:**

The online version contains supplementary material available at 10.1186/s13054-022-03937-x.

## Take home message

Clinical benefits of prone position (PP) and mechanisms underlying clinical outcomes in COVID-19-related moderate-to-severe acute hypoxemic respiratory failure treated with NIV are unknown.

In this study,

- early (i.e., initiated within 24 h of admission) prolonged (i.e., at least 8-h/day) PP therapy was feasible, safe, and was associated with reduced 28-day NIV failure, mortality and endotracheal intubation.
- a reduction in dead space indices and improved aeration and recruitment of dorso-lateral lung regions underline the observed clinical benefits
- early (day 1) increase in CO2 clearance predicted NIV success and survival in the whole study population, independently of oxygenation indices, and could help select patients for PP therapy and monitor NIV adequacy.

## Introduction

Acute hypoxemic respiratory failure is the most frequent life-threatening complication of severe Acute Respiratory Syndrome Corona Virus 2 (SARS‐CoV‐2) infection. Despite ongoing pharmacological trials, the treatment of patients with Coronavirus disease 2019 (COVID-19) pneumonia and moderate-to-severe respiratory failure remains supportive, with up to 60% of these patients requiring invasive mechanical ventilation and suffering from a mortality ranging 40–81% [1–3]. Hence, noninvasive strategies reducing the need for invasive mechanical ventilation in this category of COVID-19 patients are eagerly awaited [3–5].

Prone positioning (PP) therapy is a non-pharmacological treatment which ameliorates oxygenation through several mechanisms, including improved ventilation/perfusion matching, relief of the compression of dependent lung regions from mediastinum's weight, and change in chest wall elastance [6–8]. Furthermore, PP showed benefits independently of its effects on gas exchange [9, 10].

Prolonged PP is currently recommended for invasively ventilated patients with severe acute respiratory distress syndrome (ARDS), in whom it reduced 28-day mortality [11], but its role in awake patients with moderate-to-severe acute respiratory failure is unknown. In small case series and observational studies [12, 13]. PP for short periods of time (i.e., < 3 h/day) improved oxygenation in awake patients with acute respiratory failure of varying severity due to SARS‐CoV‐2 pneumonia receiving continuous positive airway pressure (CPAP), but the durability of this effect after resupination was inconstant, and there was no evidence for a clinical benefit on hard outcomes.

Two trials found either a reduced intubation rate or no benefits from awake PP of varying duration in COVID-19 patients with a wide range of respiratory failure severity treated with high flow nasal cannula [14, 15]. In those trials, patients treated with noninvasive ventilation (NIV) had no clinical benefits from awake PP. Hence, the utility of proning noninvasively ventilated COVID-19 patients, who are at greatest risk of adverse outcomes, remains uncertain.

Further important knowledge gaps include the optimal timing and duration of PP, as well as underlying mechanisms and predictors of response to PP in COVID-19 patients.

Hence, we investigated in patients with acute moderate-to-severe hypoxemic respiratory failure due to SARS‐CoV‐2 pneumonia receiving NIV.

1. the effect of early (i.e., within 24 h of admission) prolonged (i.e., at least 8 h/day) PP on 28-day NIV failure, intubation, and mortality as compared with supine NIV
2. underlying physiological mechanisms and early predictors of treatment response to NIV delivered in supine and prone position

## Methods

The prone position in noninvasive ventilation (PRO-NIV) study was an investigator-initiated, open-label, single-center, non-randomized controlled clinical trial conducted at HUMANITAS Gradenigo COVID Subintensive Care Unit, Turin (Italy). Between December 16, 2021 and May 30, 2021, 28-day follow-up was completed by June 30, 2021.

The study received no fund, was approved by the Comitato Etico Interaziendale A.O.U. Città della Salute **e** della Scienza di Torino (prot. N. 0046392) on December 15, 2020 and is registered with ISRCTN clinical trial registry (study ID: ISRCTN23016116). Complete study protocol and statistical analysis plan are available in Additional file 3.

## Study design

Consecutive patients with acute moderate-to-severe acute hypoxemic respiratory failure due to SARS‐CoV‐2 pneumonia treated with NIV (CPAP or Pressure Support Ventilation, PSV) and prolonged PP (experimental group), prospectively enrolled from December 16, 2020 to May 30, 2021, were compared with a group of matched historical controls, constituted by consecutive patients with moderate-to-severe acute hypoxemic respiratory failure due to SARS‐CoV‐2 pneumonia treated with NIV (CPAP or PSV) delivered in the conventional (supine) position, in the same unit from April 1, 2020 to December 15, 2020 (Additional file 1: Figure S1**)**.

### Patients

All consecutive adult patients with confirmed severe SARS‐CoV‐2 pneumonia and acute (i.e., symptom onset < 14 days of hospital admission) moderate-to-severe hypoxemic respiratory failure (defined by a paO2/FiO2 ratio < 200 mmHg while receiving O2-therapy through either a Venturi mask with FiO2 50% or a non-rebreather reservoir bag mask) admitted to HUMANITAS Gradenigo Subintensive Care Unit from April 1, 2020 to May 30, 2021, who required NIV and were able to provide informed consent were eligible for inclusion.

We excluded patients who were unable or refused to provide informed consent to treatment, were pregnant, hemodynamically unstable or needed urgent endotracheal intubation (ETI), or candidates for palliative care: inclusion and exclusion criteria were the same for both arms and are detailed in the Additional file 3.

### Interventions

In both arms, patients received NIV within 24 h of admission and the duration, settings and modes of NIV were based on available literature and consolidated clinical practice [16, 17].

### Experimental arm (prone position and NIV)

PP therapy was initiated within 24 h after admission to the Subintensive Care Unit.

After a period of NIV in the supine position and written informed consent, patients were asked to remain in PP throughout the day as long as possible, with at least 1 PP session/day lasting ≥ 8 h scheduled overnight. This mandatory 8-h PP could be extended daytime and/or integrated by additional daytime sessions according to patient compliance and clinical judgement. Study design is described in Additional file 3: Figure S1.

The following five steps were followed when undertaking PP therapy [18]: preparation, position, placement of interface, position optimization, and monitoring (see protocol). Ventilator settings were unchanged when turning from supine to PP. Patient position was continuously monitored with vital signs and recorded hourly on a predefined form (provided at the end of the protocol).

Patients completing at least one 8-h proning session/day for the initial two calendar days were considered to have successfully completed PP therapy, while those who did not were considered to have failed PP therapy.

Termination of PP procedure was considered whether the patient maintained the following conditions in the supine position, for at least 2 h following the last PP session:

- PaO2/FiO2 > 300 with FiO2 ≤ 40%, and respiratory rate ≤ 24/min during NIV.
- SpO2 ≥ 92% with FiO2 ≤ 40% via Venturi mask or via nasal cannula oxygen 10 l/m and RR ≤ 24/min and no signs of altered respiratory mechanics.

Proning procedure was resumed if patient’s clinical status or oxygenation deteriorated.

### Matching controls (NIV supine)

The controls were selected among consecutive patients with acute hypoxemic respiratory failure due to SARS‐CoV‐2 pneumonia treated in the HUMANITAS Gradenigo Subintensive Care Unit with NIV (CPAP or PSV) delivered in the conventional (supine) position from April 1, 2020 to December 15, 2020.

All controls had the same enrollment criteria described for the experimental arm. The physician who made the selection was not aware of the results of the study and of the evolution of the treatment.

To reduce the risk of bias due to confounders, propensity score (PS) analysis was performed to match PP and control group for the following baseline and treatment-related variables (see Statistics):

- paO2/FiO2 ratio while receiving inspired oxygen by Venturi or reservoir mask on admission to the Subintensive Care Unit within the same category (paO2/FIO2 ratio of 150–199 or 100–149 or < 100) of the PP patients and arterial pH within 0.04 of the values of the PP treatment patients.
- age
- BMI
- severity of illness on admission as assessed by the Simplified Acute Physiology Score (SAPS) II
- in-hospital mortality risk as assessed by the International Severe Acute Respiratory and Emerging Infections Consortium Coronavirus Clinical Characterization Consortium (ISARIC 4C) score [19],
- time from symptom onset to hospital admission
- time from hospital admission to NIV initiation
- pharmacological treatment with steroids, enoxaparin, remdesivir, and tocilizumab
- ventilatory mode (CPAP vs. PSV)

## Standard care

Both groups received NIV via standard compressed gas ICU ventilators (Dräger Savina® 300 by Draeger, BPL Elisa 600 by Lowenstein Medical, Kronsaalsweg, Hamburg, Germany) or turbine-powered air source ventilators (iVent 201 GE, Versamed Medical Systems. Haifa, Israel) in pressure support ventilation (PSV) or continuous positive airway pressure (CPAP) mode.

Interfaces were full-face masks (PerfomrMax SAU W/SE, Philips Respironics, Pennsylvania, USA**)** and dedicated helmets for CPAP (CaStar, Starmed Intersurgical, UK) or for NIV (Dimar, Modena, Italy).

Initial ventilatory settings (detailed in Additional file 3 and described in Additional file 2: Table S1**)** were chosen in the supine position and maintained during PP. Any modifications in settings and interface to optimize comfort and patient-ventilator interaction were left at the discretion of the attending physicians, but positive end-expiratory pressure (PEEP) had to be kept ≥ 10 cm H2O with helmet and ≥ 5 cmH2O with face mask.

Criteria for NIV discontinuation and discharge from Subintensive Care Unit, monitoring, hemodynamic management are detailed in study protocol. Mild intravenous sedation and analgesia were allowed according to the physician’s decision and protocol recommendations.

### Treatment failure

The decision to terminate NIV support was made by the attending physician in conjunction with an experienced Intensivist who was unaware of the study results, and was based on any of the following predefined criteria [16, 17]: persisting or worsening respiratory failure (RR > 40/min, respiratory-muscle fatigue, copious tracheal secretions, respiratory acidosis, persisting SpO2 < 90% with FIO2 ≥ 0.8 or paO2/FiO2 < 100), intolerance to devices, hemodynamic instability, neurologic status deterioration.

## Outcomes

The primary outcome was the occurrence of NIV failure within 28 days of enrolment, defined as intubation OR death.

Secondary clinical outcomes censored at 28 days after enrolment were:

1. death;
2. intubation (after excluding patients with a do-not-intubate, DNI, order);
3. time to NIV failure/intubation/death;
4. daily hours of PP therapy;
5. duration of the longest PP session each day;
6. total number of PP sessions each day;
7. daily hours of NIV;
8. days of PP therapy;
9. days of NIV;
10. length of Subintensive Care Unit/hospital stay;
11. N-patients discharged from hospital
12. days of invasive mechanical ventilation
13. death in invasively mechanically ventilated patients;
14. device-related discomfort and dyspnea: via the Numeric Pain Rating Scale (NRS) and the Critical-Care Pain Observation Tool (CPOT), respectively;
15. predefined safety outcomes as prospectively recorded by investigators;

The following parameters were recorded during the initial 7 days after enrollment to explore physiological mechanisms underlying treatment response:

(1)* Lung ultrasound (LUS) indices of lung aeration and recruitment* assessed at baseline (within 24 h of enrollment) and at day 5 (details of the assessment and justification of the timing are provided in the protocol).

The severity and extent of parenchymal involvement of each of six lung regions (two anterior, two lateral, two dorsal) were scored (range 0–3) [20] and recorded by three expertized intensivists on a predefined form.

Global LUS score (corresponding to the sum of the 12 regions’ score, range 0–36) and anterior, lateral and dorsal LUS score (each ranging 0–12) were calculated.

We first internally evaluated the accuracy of LUS in staging lung disease severity against a sample of suitable CT scans (i.e., good quality images, double-blinded operators, LUS performed within 24 h of CT examination) performed in patients with SARS-CoV-2 pneumonia admitted to our Subintensive Care Unit [21] during the study period: the correlation between global LUS score and CT severity score was evaluated with the Spearman correlation coefficient with a two-tailed *p* value < 0.05 considered statistically significant.

Then, the following LUS indices were assessed:

- regional and global LUS score
- regional and global number of consolidations (N-consolidations): the number of regions with consolidated (score 3) areas, which impacts prognosis independently of overall LUS score [22]
- regional and global LUS reaeration score [23], a validated index of lung aeration and recruitment (i.e., change from consolidated, non-aerated tissue to aerated tissue) [24, 25].

**Fig. 1:**
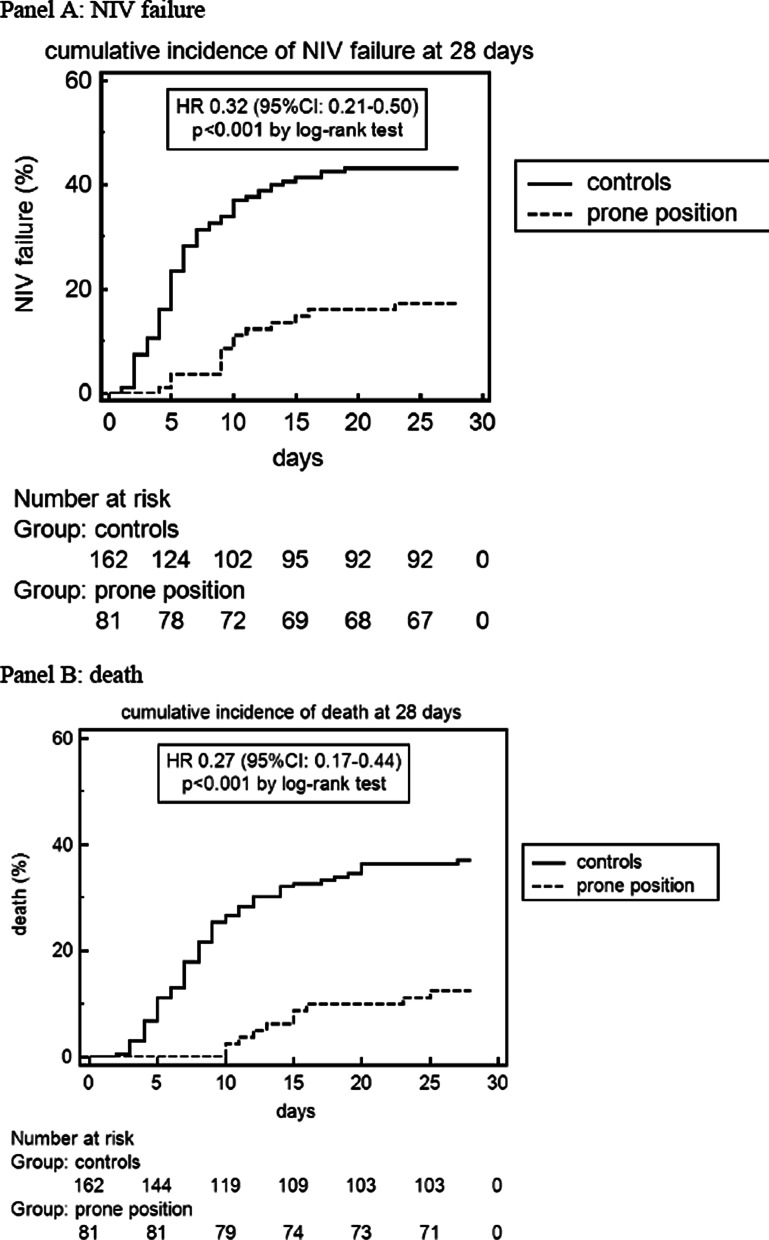

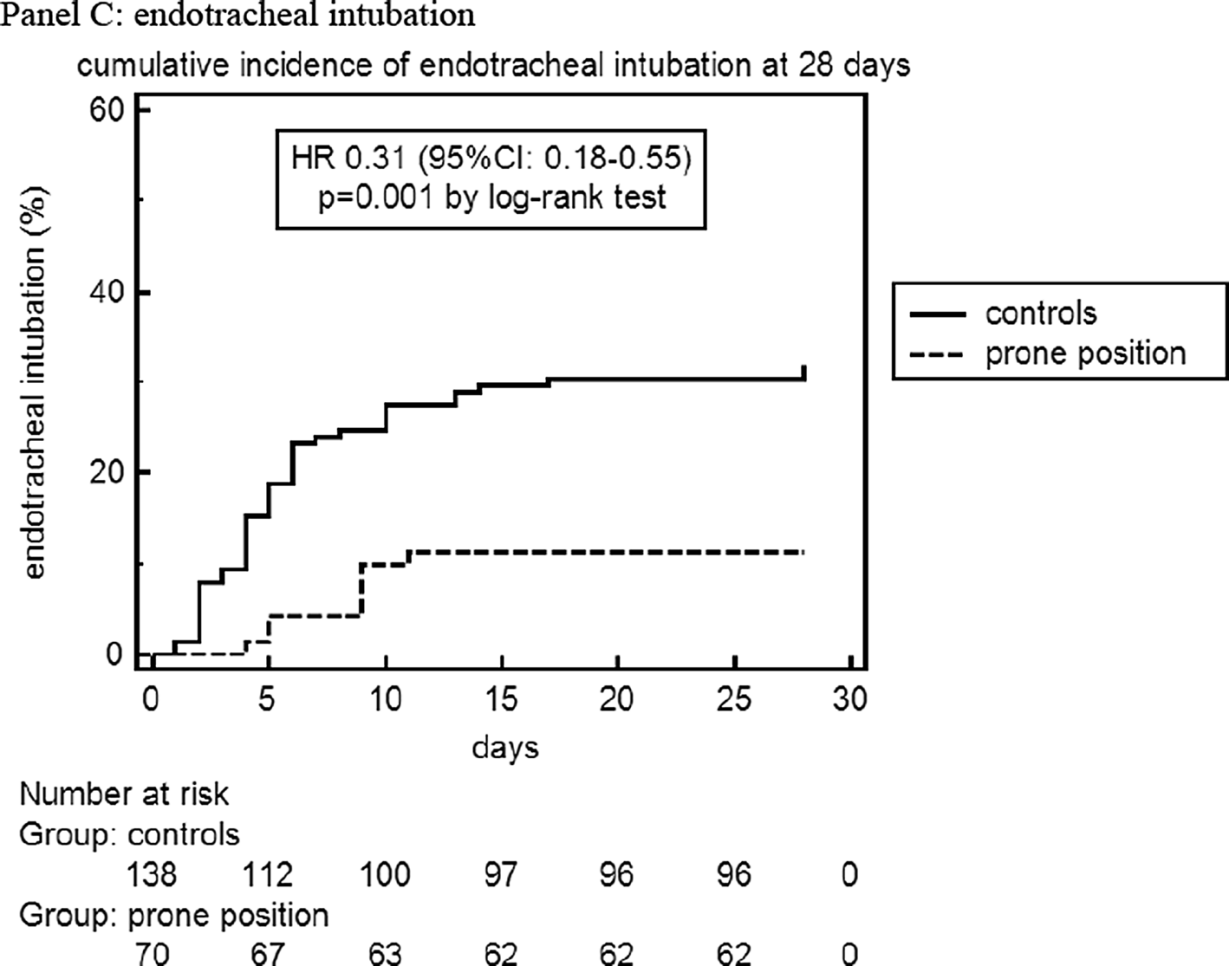
Cumulative incidence of noninvasive ventilation (NIV) failure (**A**), death (**B**) and endotracheal intubation (**C**) in the prone position and control group at 28 days after enrollment. In the endotracheal intubation group, patients with a Do-Not-Intubate (DNI) disposition were excluded

The PEEP at which each LUS examination was made was recorded.

(2)* paO2/FiO2 ratio, pCO2, respiratory rate (RR)* obtained from ABG drawn 1 h after initiating NIV in supine position, 1 h after starting the 8-h PP session and 1 h after resupination following the 8-h PP session (Fig. 1). RR and VTe were recorded at the time of each ABG.

Hypothesizing that PP during NIV homogenizes lung inflation and improves oxygenation by recruiting consolidated lung regions rather than overdistending already aerated lung, we measured the following indices of dead space and of lung aeration and recruitment:

(3)* Physiological dead space indices (DSIs): ventilatory ratio (VR) and corrected minute ventilation (MV*_*corr*_*)* These indices correlated with direct measures of dead space and predicted adverse outcomes independently of oxygenation in invasively ventilated patients with ARDS [26–28], but their validity in noninvasively ventilated patients is unknown.

Due to the inaccuracy of helmet VTe, DSIs were only calculated in patients ventilated with face mask, at the time of ABG, after achievement of ventilatory stability (defined by a ≤ 10% variation in RR and VTe and air leaks < 10% for at least 30 min).

(4)* Change in 18 blood laboratory parameters measured daily from admission to day 7,* including inflammatory and procoagulative biomarkers (detailed in protocol).

### Power analysis

Data on noninvasively ventilated patients with hypoxemic respiratory failure due to severe SARS‐CoV‐2 pneumonia report a NIV failure of 50% [29, 30], an intubation rate ranging 33–60% [4, 31], and a mortality rate ranging 40–78% [1, 2].

Assuming a 50% decrease in the risk of NIV failure, intubation and mortality to be clinically relevant, in a 1:2 ratio of experimental-to-control arm trial design and a minimal (i.e., < 5%) loss at follow-up, a total of 180 patients (60 in experimental arm and 120 in conventional arm) would be needed to detect a statistically significant (*p* < 0.05) difference between groups in NIV failure, with a beta error of 0.2, and an alpha error of 0.05.

### Statistical analysis

To reduce the risk of bias due to unbalanced groups, PS analysis was performed through a logistic regression model adjusted for the baseline and treatment-related variables previously specified. We used a greedy nearest-neighbor matching algorithm with a 1:2 matching ratio and a caliper width ≤ 0.2*SD [32].

Standardized mean differences (SMDs) between groups were calculated to assess balance in each baseline covariate, with absolute standardized differences < 0.1 indicating adequate balance between groups.

All inferential analyses were performed for all patients in the original cohort and for the propensity score-matched cohorts.

Quantitative continuous variables are presented as median (inter-quartile range, IQR) and are compared using the unpaired Student’s *t* test or the Mann–Whitney test. Normality was evaluated with the Shapiro–Wilk test. Qualitative or categorical variables are compared with the Chi-square or the Fisher’s exact test, as appropriate.

To compare continuous variables collected at different time points (i.e., respiratory and biochemical parameters), we used repeated measures two-factor (within subject and between group) ANOVA for continuous variables assessed at multiple timepoints, after log-transformation of non-normal variables.

The effect of the intervention on 28-day NIV failure, death, and endotracheal intubation (ETI) was evaluated via a time-to-event analysis with the Kaplan–Meier procedure and compared with the log-rank test. Hazard ratios (HRs) together with 95% CIs were estimated using this procedure.

We used Cox proportional multivariable regression analysis that adjusted for imbalanced variables between PP and controls in the primary dataset to assess the effect of confounders on 28-day NIV failure, death and ETI, with the maximum number of covariates allowed in each model set at (event rate × *N*)/10, where *N* is the sample size [33].

We also ran a second Cox model after including respiratory physiological variables assessed at day 1 after enrollment, to explore the role of these variables in early prediction of NIV failure, death and ETI. Interaction between time and covariates was also included.

In the pathophysiological model we used the following definitions [13]:

- O2 responders: patients who increased the paO2/FiO2 ratio during NIV in supine position at day 1 (timepoint sp1, see Fig. 3) as compared with NIV supine at baseline (timepoint sp0): i.e., pO2/FiO2sp1—pO2/FiO2sp0 > 0 or ΔpaO2/FiO2sp0-1 > 0).
- CO2 responders: patients with an increase in CO2 clearance were defined by a reduction in their dead space indices (VR and MV_corr_, tested separately) at sp1 as compared with timepoint sp0 [VRsp1 − VRsp0 < 0 (or ΔVRsp0-1 < 0) and MV_corr_sp1 − MV_corr_sp0 < 0 (or ΔMV_corr_sp0-1 < 0)].

This allowed comparing gas exchange responses between PP and control group after day 1 of NIV, in the same (supine) position, after taking into account the effect of the first PP session (timepoint pp1) in the PP group (Fig. 3).

We planned to explore dose–response relationship between PP therapy and respiratory, ultrasonographic and biochemical parameters by univariable and multivariable regression analysis, after log transformation of skewed parameters; we searched the best fit among four predictive models (linear, exponential, logarithmic, binomial) using *R*^2^ values. In these multivariable models, we used a combination of backward procedure and exclusion of highly collinear variables through model-dependent variance inflation factor (VIF) cut-off values to select covariates (see protocol).

Time change in continuous variables was assessed by computing the area under the curve (AUC) was computed by the trapezoid method [34].

All tests were performed at two-tails with significance set at a *p* value < 0.05.

PP failure was defined by the inability to keep PP for at least 8 h/day during the initial two days since enrollment in the PP arm.

In controls, PP could be considered as a rescue therapy after failure of ≥ 2 days of NIV delivered in the supine position: these patients remained in the control group in the intention-to-treat primary analysis. The day of initiation, duration and respiratory variables during rescue PP were recorded as for early PP therapy for these patients and a sensitivity analysis was planned after excluding patients with rescue PP (see below).

In the primary analysis, data were analyzed on an intention-to-treat basis with all data for patients who consented to PP (regardless of whether they successfully completed PP therapy or not).

### Prespecified secondary subgroup and sensitivity analyses

Subgroup and sensitivity analyses were planned to assess the effect of the following factors on main clinical efficacy (NIV failure, death, ETI) and safety outcomes:

- severe (paO2/FiO2 < 100) versus moderate hypoxemic respiratory failure (paO2/FiO2: 100–199) at admission;
- per protocol analysis (excluding patients with PP failure and rescue PP);
- interface (helmet vs. face mask);
- ventilatory mode (CPAP vs. PSV);
- sedation (yes/no);
- presence of complete LUS assessment (yes/no);
- controls enrolled during the 1st pandemic wave (April 1–June 30, 2020) versus those enrolled during the second pandemic wave (after June 30, 2020).
- different definition of O2 response and CO2 response in the PP group, based on gas exchange values during the first PP session (timepoint pp1) as compared with sp0 and on different thresholds for gas exchange responses [13, 35] (see protocol);
- after excluding patients with paCO2 > 45 mmHg at admission;
- for inflammatory and coagulative markers: after excluding patients treated with tocilizumab, with documented microbial infection and those with venous thromboembolism, respectively.

All analyses were carried out with MedCalc 19.7 (MedCalc Software Ltd Ostend, Belgium) and STATISTICA 5.1 statistical software package (Statsoft, Tulsa, OK). Box & whiskers and regression plots were produced with Microsoft XLSTAT 2021.1

## Results

### Characteristics at inclusion

Patient flow through the study is reported in Additional file 1: Figure S1.

The analysis of pre-post-matching SMDs, propensity score (PS) density, and logit (PS) distribution plots revealed good balance in baseline and treatment-related covariates between groups (Additional file 1: Figure S2**,** Additional file 2: Table S2).

At the end of selection, 81 consecutive patients treated with NIV delivered in PP and 162 consecutive controls treated with conventional (supine) NIV were included in the study (Table 1).

**Table 1:** Baseline features of included patients, grouped according to study intervention (*n* = 243)

Parameter	Controls	Prone position	*p*
	(*n* = 162)	(*n* = 81)	
Age (years)	69 (61–78)	68 (60–75)	0.498
Male sex (*n*, %)	116 (72%)	62 (76%)	0.401-
Race			
White, non-hispanic	155 (95%)	75 (93%)	0.401
White, hispanic	6 (4%)	4 (5%)	0.738
Black	1 (1%)	2 (2%)	0.613
Time from symptom onset to hospital admission( d)	7 (5–10)	7 (5–10)	0.676
Time from hospital admission to enrollment (d)	2 (1–3)	2 (1–3)	0.966
BMI (kg/m^2^)	27.5 (25.1–31.7)	27.3 (25.0–31.2)	0.505
Obesity (BMI ≥ 30 kg/m^2^) *n *(%)	48 (30%)	23 (29%)	0.612
Type 2 diabetes mellitus *n *(%)	32 (20%)	17 (21%)	0.667
Hypertension *n *(%)	94 (58%)	45 (56%)	0.419
Chronic lung disease			
COPD	26 (16%)	16 (19%)	0.679
Asthma	5 (3%)	3 (4%)	0.513
Coronary heart disease *n* (%)	13 (8%)	8 (9%)	0.815
Chronic atrial fibrillation *n* (%)	7 (4%)	4 (5%)	0.771
Chronic kidney disease *n* (%)	16 (10%)	8 (10%)	0.982
History of cancer *n* (%)	4 (2%)	3 (4%)	0.512
Immunocompromised state^a^	5 (3%)	3 (4%)	0.887
Smoking status			
Former (%)	23 (14%)	16 (19%)	0.513
Current (%)	14 (8%)	6 (7%)	0.395
ISARIC 4 C mortality score	14 (10–15)	14 (11–15)	0.536
SAPS II score	36 (31–39)	35 (32–40)	0.727
Temperature (°C)	36.5 (36–36.9)	36.4 (36–36.8)	0.821
Sys BP (mmHg)	130 (120–140)	130 (116–140)	0.767
Dia BP (mmHg)	74 (65–80)	75 (68–80)	0.839
Heart Rate (beats/min)	85 (71–96)	81 (70–99)	0.744
Respiratory rate (breaths/min)	28 (22–30)	28 (23–30)	0.953
PaO2/FiO2 ratio	102 (78–140)	104 (80–142)	0.539
PaO2/FiO2 ratio category *n* (%) patients			
150–199	28 (17%)	14 (17%)	0.999
100–149	54 (33%)	27 (33%)	0.999
< 100	80 (49%)	40 (49%)	0.999
paCO2 (mmHg)	34 (31–37)	35 (31–39)	0.484
paCO2 > 45 mmHg at admission *n*(%)	3 (2%)	2 (2%)	0.739
Arterial pH	7.47 (7.43–7.49)	7.46 (7.44–7.49)	0.680
Adjuvant therapies for COVID-19			
Corticosteroids	162 (100%)	81 (100%)	0.999
Dexamethasone	156 (96%)	100%	0.313
− 10 mg/day	38 (23%)	0%	0.319
− 6 mg/day	118 (73%)	81 (100%)	0.138
Methylprednisolone 40 mg/day	5 (4%)	0%	0.615
Remdesivir	1 (1%)	1 (1%)	0.818
Tocilizumab	4 (2%)	0 (0%)	0.897
Convalescent plasma	1 (1%)	0 (0%)	0.981
Enoxaparin	155 (96%)	78 (96%)	0.912
Prophylactic dose	68 (42%)	36 (44%)	0.391
Intermediate dose^b^	16 (16%)	14 (16%)	0.223
Anticoagulant dose	71 (38%)	28 (36%)	0.749
Warfarin/DOACs	7 (4%)	4 (5%)	0.786
Antibiotics			
Azithromycin	11 (17%)	8 (10%)	0.412
Beta-lactams	100 (62%)	48 (59%)	0.749
Others	5 (3%)	2 (2%)	0.813
Antifungal	2 (1%)	1 (1%)	0.702
Any SARS-CoV-2 vaccine	0 (0%)	0 (0%)	0.999

For each parameter median (IQR) is indicated, unless otherwise specified

The *p* values value refer to comparison between groups at baseline, at the end of follow-up and to comparison in changes during the follow-up, respectively. Data are expressed as mean ± SEM

DOAC, direct oral anticoagulants; ISARIC, International Severe Acute Respiratory Infection Consortium; NIV, noninvasive ventilation; SAPSS, Simplified acute physiology score

^a^HIV, ongoing chemotherapy, chronic immunosuppressor therapy

^b^Prophylactic dose twice daily

Apart from patient position during NIV, the staff, equipment, standard care, and monitoring were the same for both groups.

Baseline patient demographics, clinical features, and adjunctive therapies did not differ between PP and controls (Table 1). Dexamethasone use was implemented early in the course of the pandemic, based on recommendations by Italian Society of Infective and Tropical diseases issued on March 13, 2020 [36]

### Treatments

Patients initiated NIV and PP within 24 h of admission to the Subintensive Care Unit.

The median (IQR) duration of NIV and of PP during the initial 7 days are represented in Additional file 1: Figure S3: over the initial 48 h of treatment, NIV was delivered continuously or until intubation and only brief interruptions were allowed for eventual adjustments and nursing care, lasting no more than few minutes; subsequently, daily breaks, lasting no more than 2 h, were allowed for meals and nursing care, depending on patient clinical condition and tolerance.

Daily hours of NIV and PP, the duration of the longest PP session and the daily number of PP sessions at 7 days are reported in Table 2.

**Table 2:** Efficacy and safety outcomes at 28 days in patients included in the primary (intention-to-treat) analysis, grouped according to study intervention (*n* = 243)

Outcome	Controls (*n* = 162)	Prone position (*n* = 81)	Absolute or mean difference (95% CI)	HR (95% CI )	*p*
*Primary outcome*					
NIV failure at 28 days	70 (43%)	14 (17%)	− 26% (− 17%, − 41%)	0.32 (0.21, 0.50)	**< 0.001**
*Secondary outcomes*					
Death at 28 days	59 (36%)	10 (12%)	− 24% (− 15%, − 39%)	0.27 (0.17, 0.44)	**< 0.001**
ETI at 28 days^a^	44 (30%)	8 (11%)	− 19% (− 13%, − 33%)	0.31 (0.18–0.55)	**0.001**
Length of stay in subintensive care unit (d)	7 (5, 9)	6 (5, 8)	− 1 (− 2, 0)		**0.047**
Days of invasive mechanical ventilation (d)	8 (2, 14)	6 (2, 12)	− 1 (− 2, 2)		0.618
Death in invasively ventilated mechanically ventilated patients	27 (64%)	2 (25%)	− 39% (− 20% to − 85%)	0.39 (0.24–0.67)	**0.034**
Length of hospital stay (d):					
Whole study population	16 (12, 20)	15 (10, 20)	0 (− 1, 0)		0.156
Hospital survivors	19 (15, 22)	15 (10, 20)	− 3 (− 5, − 1)		**0.040**
Discharged from hospital *n* (%)	101 (62%)	70 (86%)			**< 0.001**
Daily hours of NIV	20.3 (15.9, 22.2)	20.1 (18.2, 22.4)	− 1 (0, − 1)		**0**.738
Total days of NIV at 28 days	7 (5, 9)	6 (5, 8)	− 1 (− 2, 0)		**0.047**
Daily hours of PP	–	12.2 (10.1, 13.8)	−		
Duration of the longest PP session each day	–	10.9 (9.1, 13.3)	–		
Number of PP sessions each day	–	2 (1, 3)	–		
Total days of PP therapy at 28 days	–	6 (5, 8)	–		
*Safety endpoints*					
Back pain	14 (9%)	10 (12%)			0.512
Intravenous/arterial line dislodgement	10 (6%)	5 (6%)			0.825
Hemodinamic instability	0 (0%)	0 (0%)			0.999
Barotrauma^b^	4 (3%)	4 (4%)			0.831
Pneumothorax/	1 (1%)	0 (0%)			
Pneumomediastinum	4 (3%)	4 (5%)			
Subcutaneous emphysema	3 (2%)	3 (4%)			
Gastric distension and vomiting	0 (0%)	0 (0%)			0.999
Device-related: nasal skin ulceration	3 (2%)	2 (2%)			0.816
Facial edema	5 (3%)	6 (7%)			0.513
Thoraco-abdominal wall hematoma	2 (1%)	3 (3%)			0.639
Venous thrombosis					0.315
Upper limb	5 (3%)	4 (5%)			
Lower limb	0	0			
Subintensive Care Unit-acquired Infection	12 (7%)	8 (10%)			0.332
Excessive sedation^c^	1 (1%)	1 (1%)			0.911
Acute kidney injury requiring renal replacement therapy	1 (1%)	1 (1%)			0.974
Liver failure	0 (0%)	0 (0%)			0.999
Need for emergency ETI	0 (0%)	0 (0%)			0.999
Time to NIV failure (d)	5 (3, 9)	9 (5, 13)			**0.031**
Time to death (d)	8 (6, 11)	14 (10, 16)			**0.013**
Time to ETI (d)^a^	5 (2, 8)	9 (4, 10)			**0.044**
Reason for ETI^a,b^					
Worsening or non-improving hypoxemia	44 (32%)	8 (11%)			**0.001**
Respiratory muscle fatigue	22 (16%)	6 (9%)			0.209
Worsening or unbearable dyspnea	40 (29%)	4 (6%)			**< 0.001**
Intolerance to treatment	4 (3%)	3 (4%)			0.316
Altered mental status	1 (1%)	0 (0%)			0.741
Shock	0 (0%)	0 (0%)			0.999
Hypercapnia	1 (1%)	0 (0%)			0.741
Inability to clear secretions	1 (1%)	0 (0%)			0.741
Extracorporeal membrane oxygenation	1 (1%)	0 (0%)			0.639

ETI, endotracheal intubation; NIV, noninvasive ventilation

^a^Among patients with a full treatment indication (*n* = 208)

^b^Subcategories are not mutually exclusive and may not necessarily sum to the category total

^c^Defined by a RASS < − 3 for more than 30 min

Statistically significant differences are written in bold character

There was no difference in initial ventilatory settings and parameters between the two groups; during the initial 7 days, the two groups showed similar VTe and MV, while patients in the PP group had higher median paO2/FiO2 ratio, lower paCO2, RR, dyspnea (CPOT score), dead space indices (VR and MVcorr) and required lower median PEEP and FiO2 than controls (Additional file 2: Table S3).

In the PP arm, 1 patient failed PP therapy and was intubated on day 1 after 7 h of PP therapy; in controls, 10 (6%) patients underwent rescue PP therapy.

As per clinical decision, continuous infusion of the short-acting sedative dexmedetomidine was used in 55 patients (67%) in the PP group and in 56 patients (35%) in the controls (*p* = 0.002).

### Efficacy and safety outcomes at 28 days

No patient was lost to follow-up, and there were no missing data for the primary, secondary, and safety end-points.

Results of the primary, intention-to-treat analysis for efficacy and safety outcomes at 28 days are reported in Table 2 and Fig. 1.

NIV failure rate was 43% in controls versus 17% in the PP group (absolute difference, − 26%; 95% CI − 17 to − 41%; unadjusted HR: 0.32, 95% CI 0.21–0.50, *p* < 0.0001).

Mortality was 36% in controls versus 12% in the PP group (absolute difference, − 24%; 95% CI − 15 to − 39%; unadjusted HR: 0.27, 95% CI 0.17–0.44, *p* < 0.0001).

After excluding patients with a DNI order (15% in controls and 14% in the PP group; *p* = 0.891), 138 patients in controls and 70 patients in the PP group had a full-treatment code. The rate of ETI among these patients was 32% in controls versus 10% in the PP group (absolute difference, − 22%; 95% CI − 13 to − 39%; unadjusted HR: 0.31, 95% CI 0.18–0.55, *p* = 0.0012).

Kaplan–Meier curves showed no evidence against the assumption of proportionality.

Among the other outcomes assessed at 28 days, the PP group showed a lower ICU mortality among invasively ventilated patients, fewer days of NIV and of Subintensive Care Unit stay, a lower dyspnea severity (by CPOT), and a shorter hospital stay among hospital survivors (Table 2).

In a Cox proportional multivariable model adjusting for baseline variables associated with outcomes at univariable analysis, PP therapy remained independently associated with 28-day NIV failure, death and ETI (Additional file 2: Tables S4–S7).

### Safety endpoints at 28 days

The incidence of adverse events was low and not statistically different between the two groups (Table 2). No patient required emergency ETI and median time to NIV failure, ETI and death was longer in the PP group as compared with controls.

Early prolonged PP therapy was associated with lower NIV failure, death and ETI rates in patients with both severe (paO2/FiO2 < 100 at admission) and moderate hypoxemic respiratory failure (paO2/FiO2 100–199 at admission) and when comparing PP group with controls either from the first or the second pandemic wave **(**Additional file 1: Figure S4, S5).

### Physiological study at 7 days

We next explored mechanisms associated with the observed clinical outcomes.

Over the initial 7 days, there were missing data in the physiological parameters due to the occurrence of NIV failure or success and subsequent unit discharge. Because these data were not missing at random but due to the consequence of treatment effect, we did not perform multiple imputation and excluded missing values from analyses.

### Ultrasonographic indices of lung aeration and recruitment

*Validation of LUS findings vd computed tomography (CT)* During the study period, 189 chest CT scans were performed in patients during their Subintensive Care Unit stay; 158 patients had a CT scan at admission and 31 of them repeated a CT scan during their stay.

**Fig. 2:**
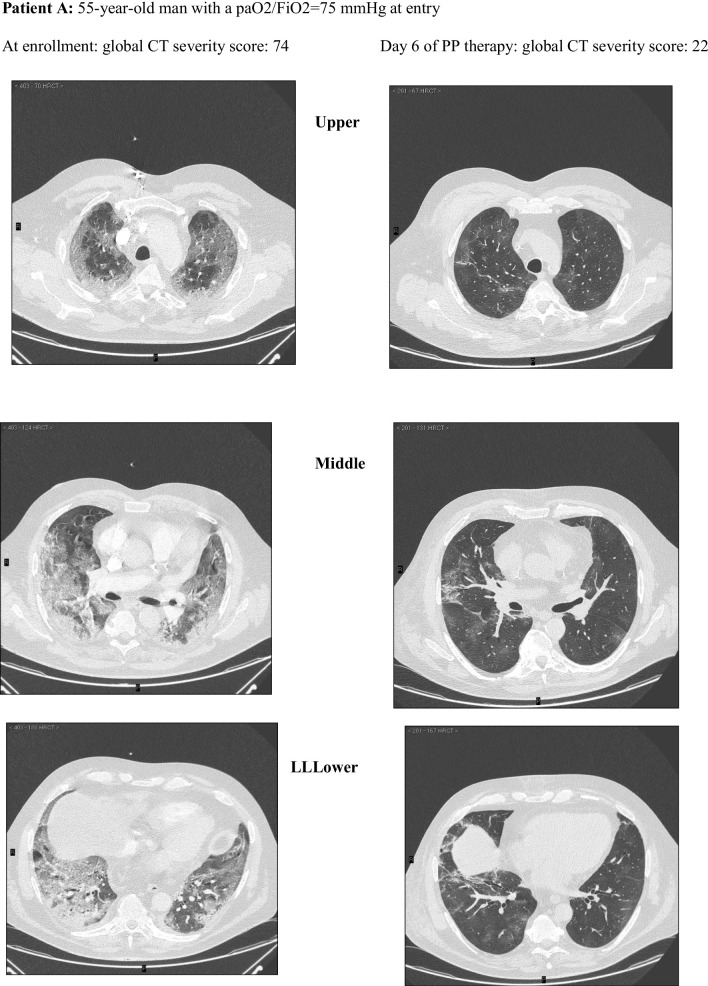

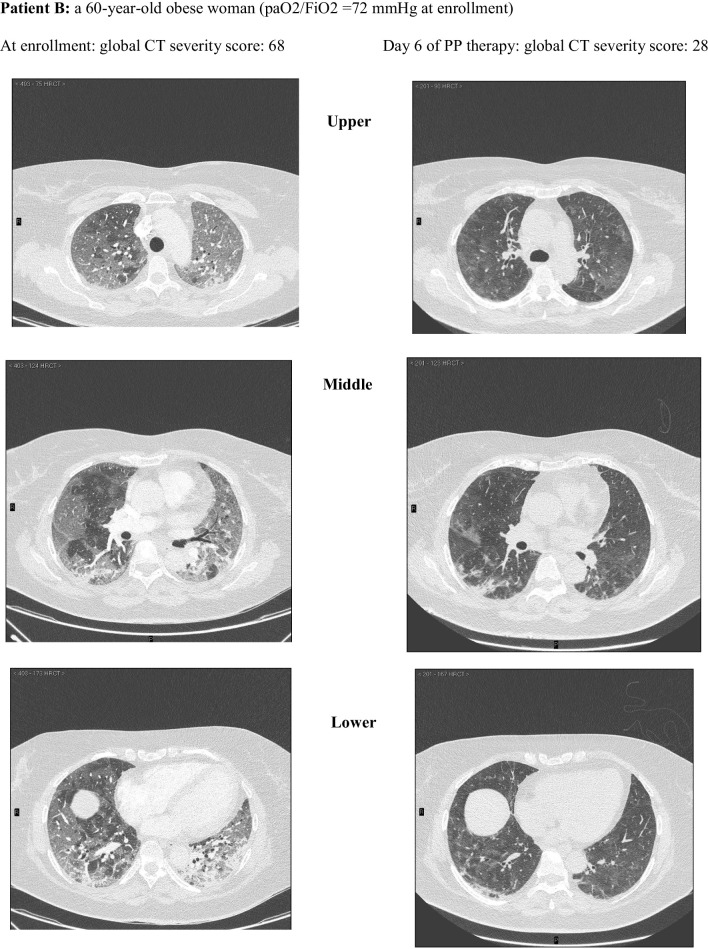
Extent and nature of lung parenchymal involvement at three levels (upper, middle, lower) were quantified according to the score validated by Salaffi et al. (score range 0–96). Two cases with pre/post-treatment CT scans are provided below

Among these, 162 CT scans were suitable for comparison with an equal number of LUS examinations and were reviewed and scored according to a validated CT severity score [37] by an experienced radiologist (FA) blinded to clinical and ultrasonographic data **(**Fig. 2). The analysis of CT scans and LUS reports yielded a median (IQR) global CT severity score of 46 (32, 60) and a global LUS severity score of 25 (21,30), respectively.The correlation between global LUS and global CT severity scores was consistent with existing literature [38]: *r*_s_ = 0.84 (95% CI 0.78–0.89; *p* < 0.0001) (Additional file 1: Figure S6).

187 patients (81 in the PP group and 106 controls) had a LUS examination at baseline and at day 5 since enrollment, available for pre/post-treatment comparison. The clinical and respiratory features of controls with LUS were comparable to those without LUS (see sensitivity/subgroup analyses).

**Fig. 3:**
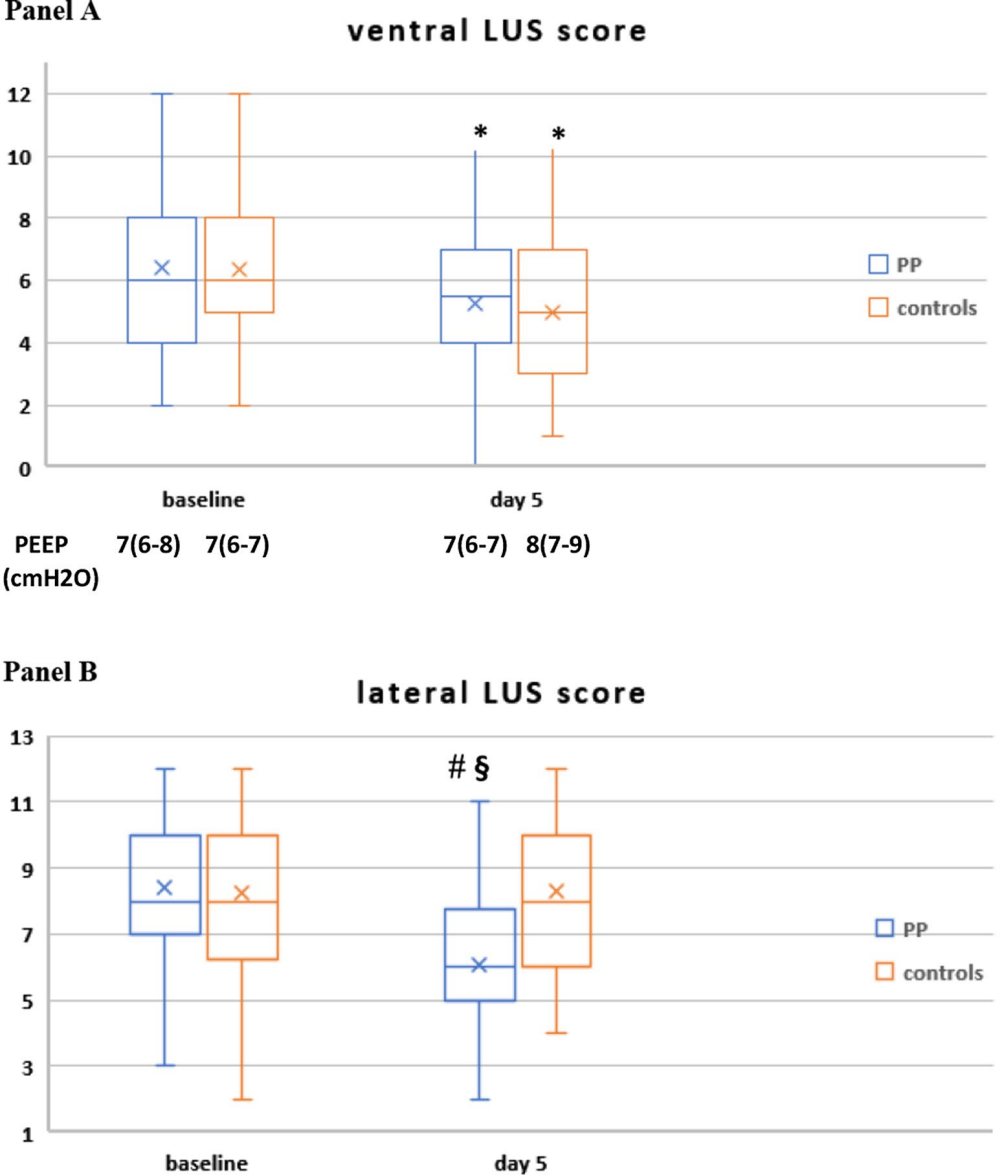

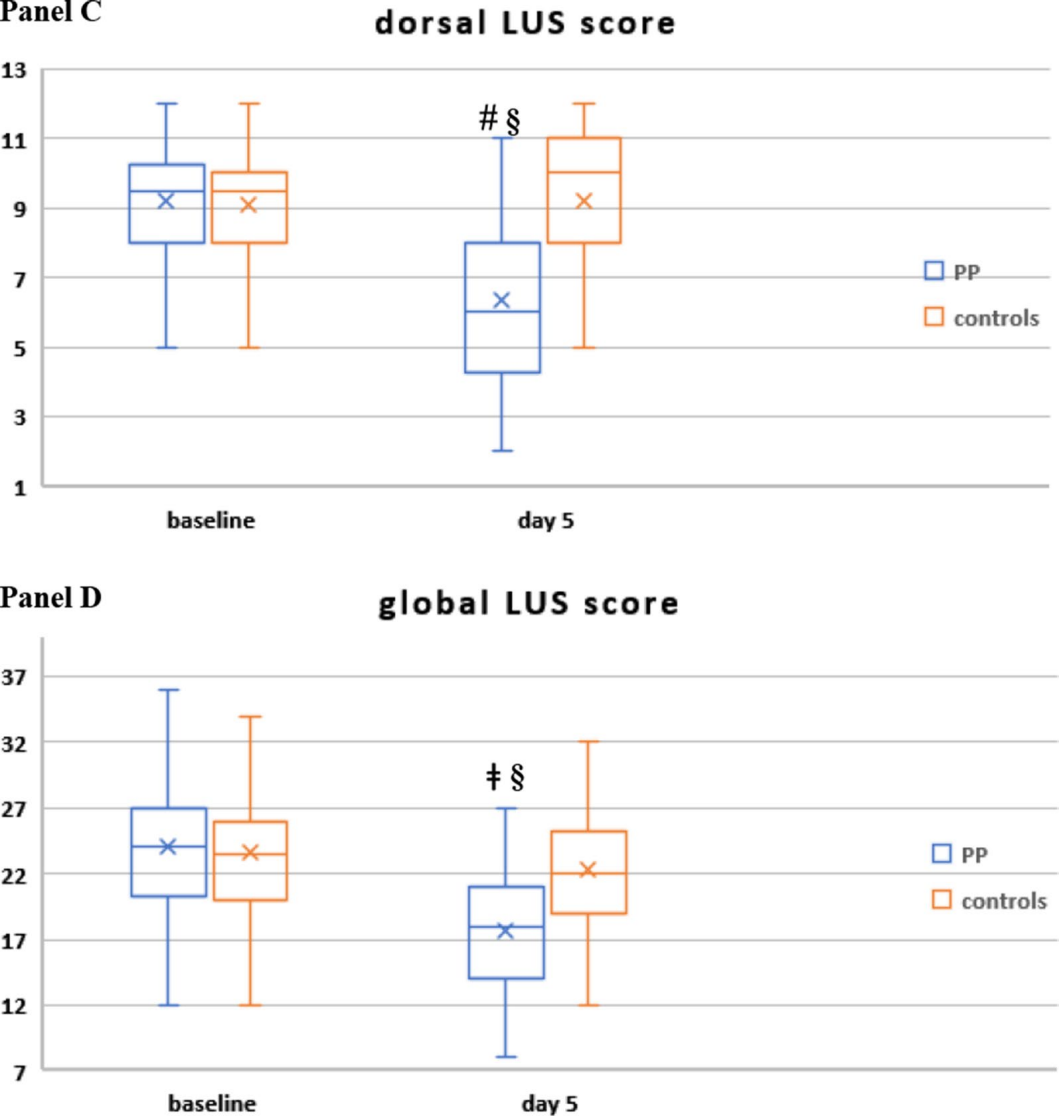

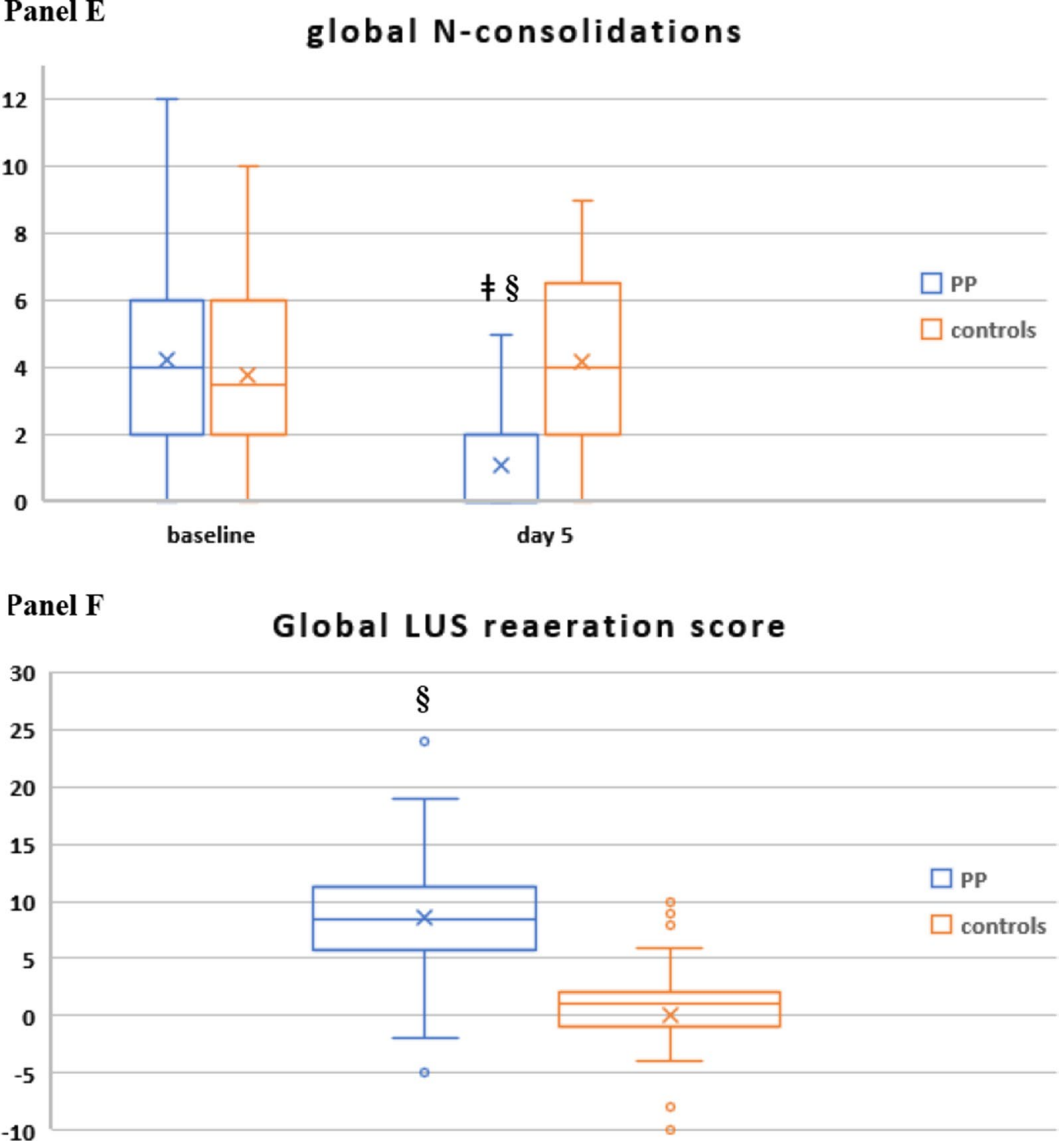
Change in regional and global LUS score (**A**–**D**), change in global number of regions with consolidated areas (N-consolidations; **E**) and global LUS reaeration score at day 5 (**F**) in study population (*n* = 187). In the box plots the middle line represents the median observed value, boxes represent the interquartile range (IQR), whiskers extend to the most extreme observed values with 1.5 times the interquartile range of the nearer quartile, and dots represent observed values outside that range. The cross represents the mean value within each box plot

Patients in the PP group showed a significant reduction in global LUS score and in N-consolidated regions and a higher global LUS reaeration score than controls, who slightly improved only anterior LUS score. The improvement in global LUS indices observed in the PP group was driven by more reaeration in the dorso-lateral lung regions (Fig. 3, Additional file 2: Table S8) and was associated with reduced 28-day NIV failure, death and ETI (not shown).

Notably, 57 (70%) patients in the PP group versus 5 (3%) controls showed a LUS reaeration score ≥ 8 (*p* < 0.0001), which corresponded to a lung recruitment greater than 600 ml in the study by Bouhemad [23].

### Oxygenation, respiratory rate and dead space indices (DSIs)

Indices of dead space were calculated in 182 patients initially ventilated with face mask. The features and outcomes of patients ventilated with face mask were similar to those of patients ventilated with helmet (see sensitivity/subgroup analyses).

**Fig. 4:**
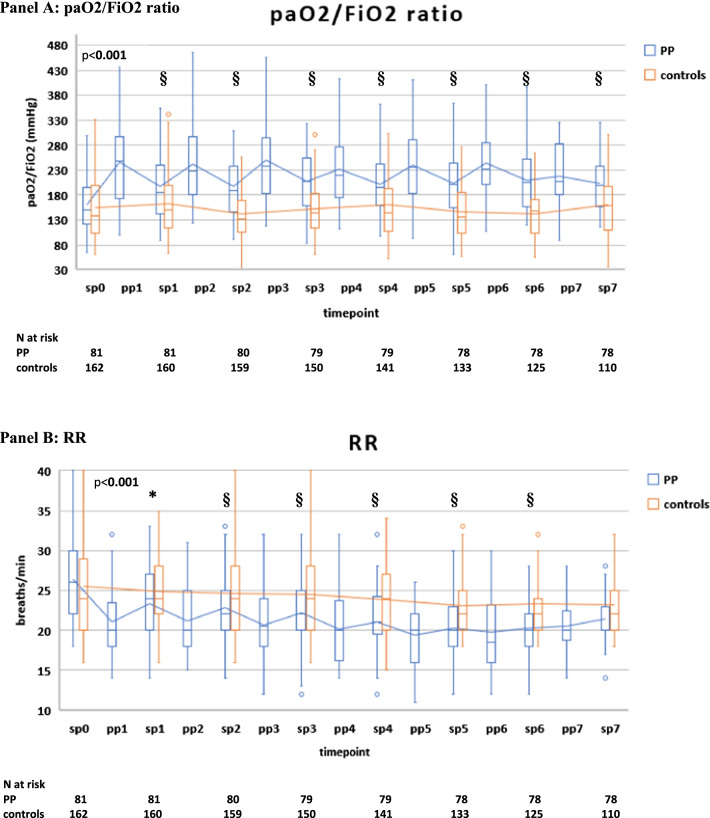

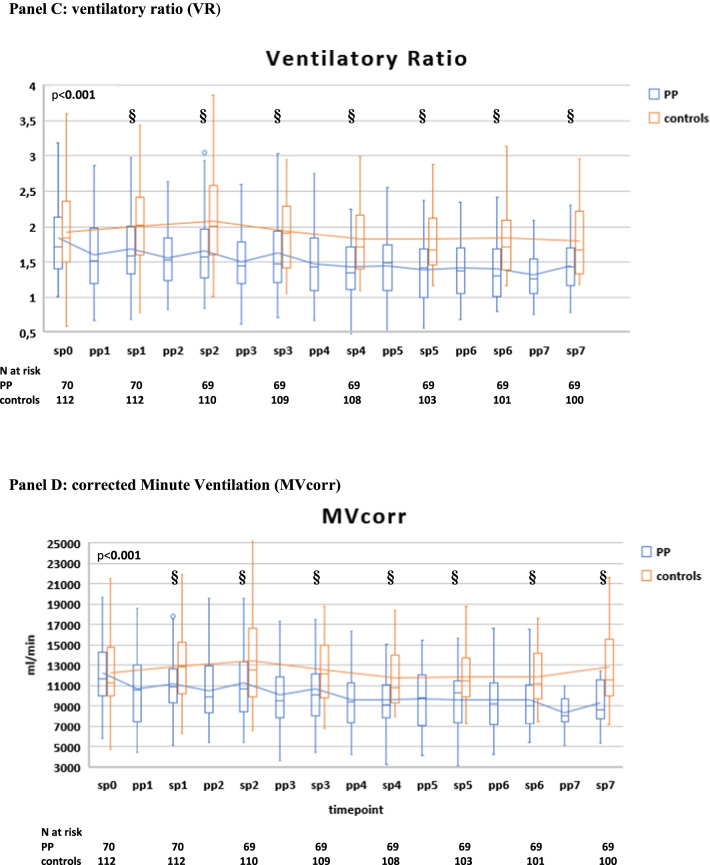

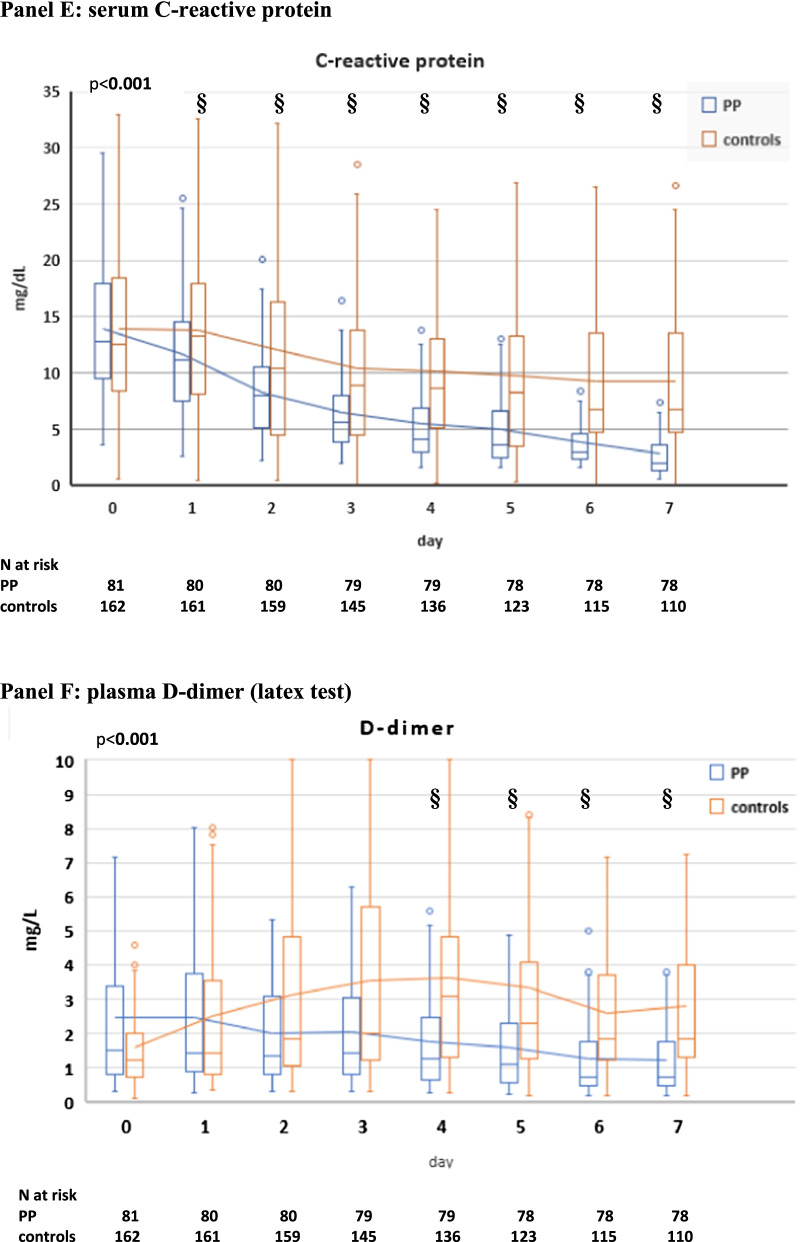

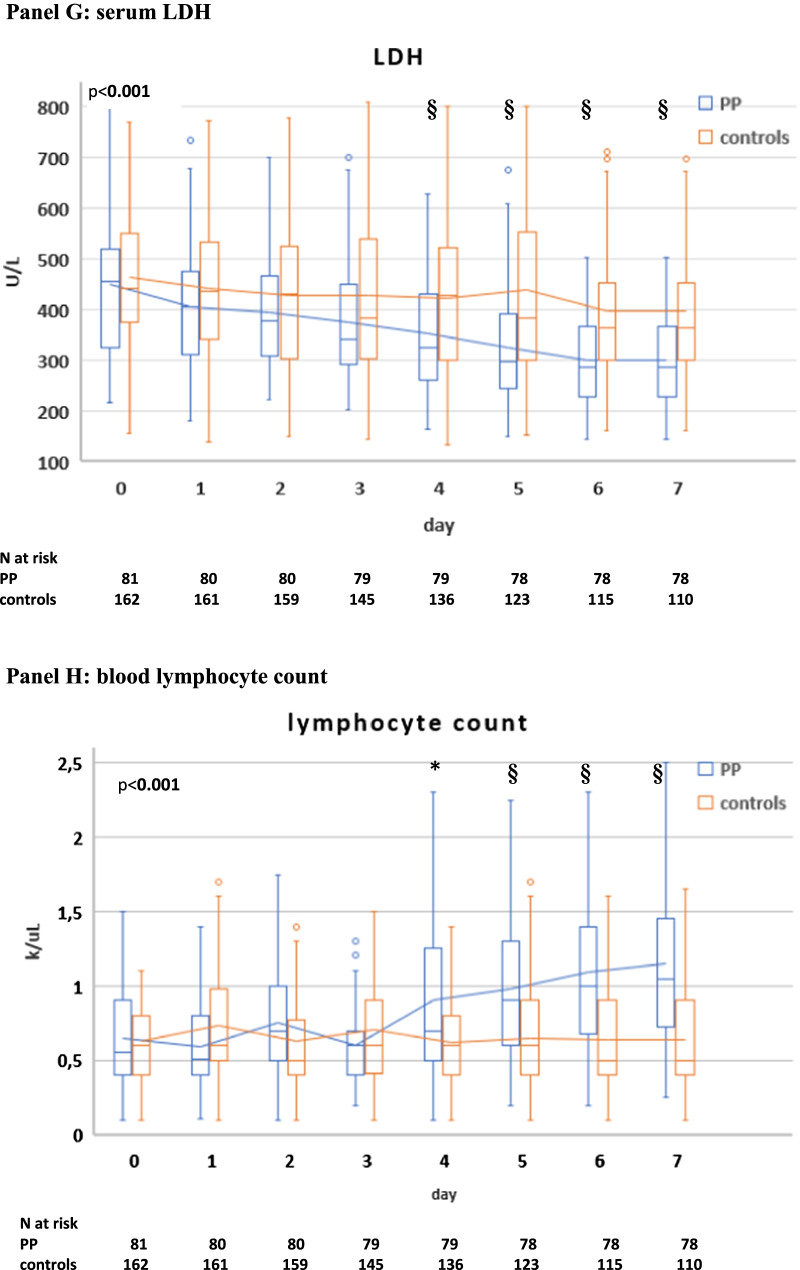

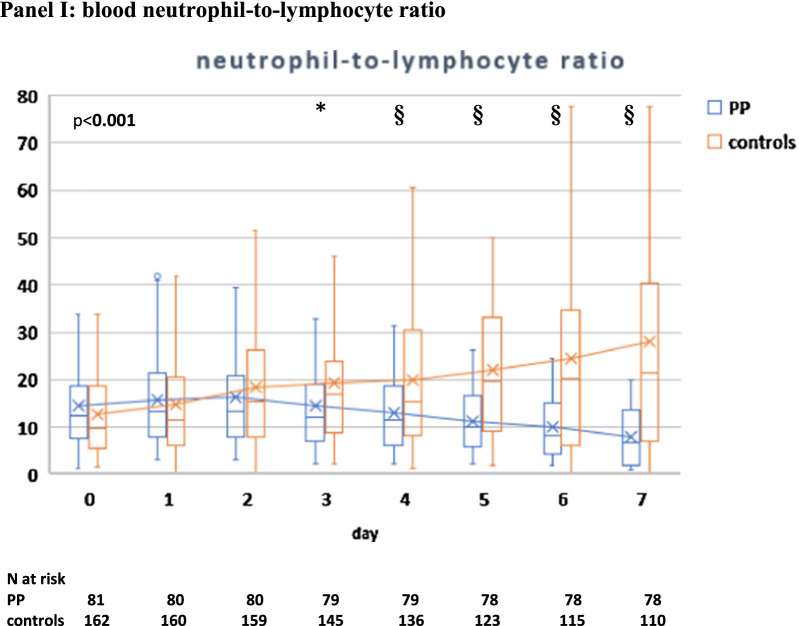
Gas exchange, respiratory rate (RR) and blood biomarker responses during NIV in the PP group and controls. The following parameters are represented: paO2/FiO2 ratio (**A**), RR (**B**), ventilatory ratio (VR) (**C**), corrected Minute Ventilation (MVcorr) (**D**), serum C-reactive protein (**E**), plasma D-dimer (latex test, **F**), serum LDH (**G**). Blood lymphocyte count (**H**), neutrophil-to-lymphocyte ratio (**I**). Patients were censored from the analysis after NIV failure or success. In the box plots, the middle line represents the median observed value, boxes represent the interquartile range (IQR), whiskers extend to the most extreme observed values with 1.5 times the interquartile range of the nearer quartile, and dots represent observed values outside that range. The connecting line connects the mean values within each box plot at different time points. The PP group had a mandatory night PP session of at least 8 h; this session could be extended though the daytime or additional daytime sessions could be delivered according to clinical status and patient compliance. All ABGs were drawn during NIV, at least 1 h after assuming PP or supine position. ABG, arterial blood gas analysis; BC, blood chemistry; LUS, lung ultrasound; PP, prone position; SUP, supine position. The timepoints are indicated as follows: sp0: supine position timepoint 0 (baseline, after NIV initiation), pp1: during the first PP session in the PP group; sp1: supine position timepoint 1 (after the initial 24 h of NIV; in the PP group this also corresponded to the resupination after the first PP session) on day 1, pp2: during the second PP session, sp2: supine position timepoint 2 (after the second day of NIV, in the PP group this also corresponded to the resupination after the second PP session on day 2. pp3 during the third PP session, sp3 supine position timepoint 3 (after the third day of NIV, in the PP group this also corresponded to the resupination after the third PP session on day 3. pp4 during the fourth PP session, sp4 supine position timepoint 4 (after the 4th day of NIV, in the PP group this also corresponded to the resupination after the fourth PP session on day 4. pp5 during the fifth PP session, sp5 supine position timepoint 5 (after the fifth day of NIV, in the PP group this also corresponded to the resupination after the fifth PP session on day 5. pp6 during the sixth PP session, sp6 supine position timepoint 6 (after the sixth day of NIV, in the PP group this also corresponded to the resupination after the sixth PP session on day 6. pp7 during the seventh PP session, sp7 supine position timepoint 7 (after the seventh day of NIV, in the PP group this also corresponded to the resupination after the seventh PP session on day 7

Compared with controls, PP therapy was associated with an increase in paO2/FiO2 and a reduction in RR and DSIs during PP sessions and in supine position (Fig. 4A–D, Additional file 2: Table S3).

Between-group difference in supine position became statistically significant at timepoint sp1 (corresponding in the PP group to the first resupination after the first PP session) and remained significant throughout the initial 7 days (Fig. 4A–D).

In the PP group**,** daily swings in RR and DSIs between prone and supine position subsided after day 5 (timepoint pp5) [P supine vs. prone < 0.05 for RR, VR, MV_corr_), suggesting no additional effect on these parameters from further PP days **(**Fig. 4A–D).

### Relationship of early (day 1) gas exchange responses with clinical outcomes and LUS reaeration and recruitment

**Fig. 5:**
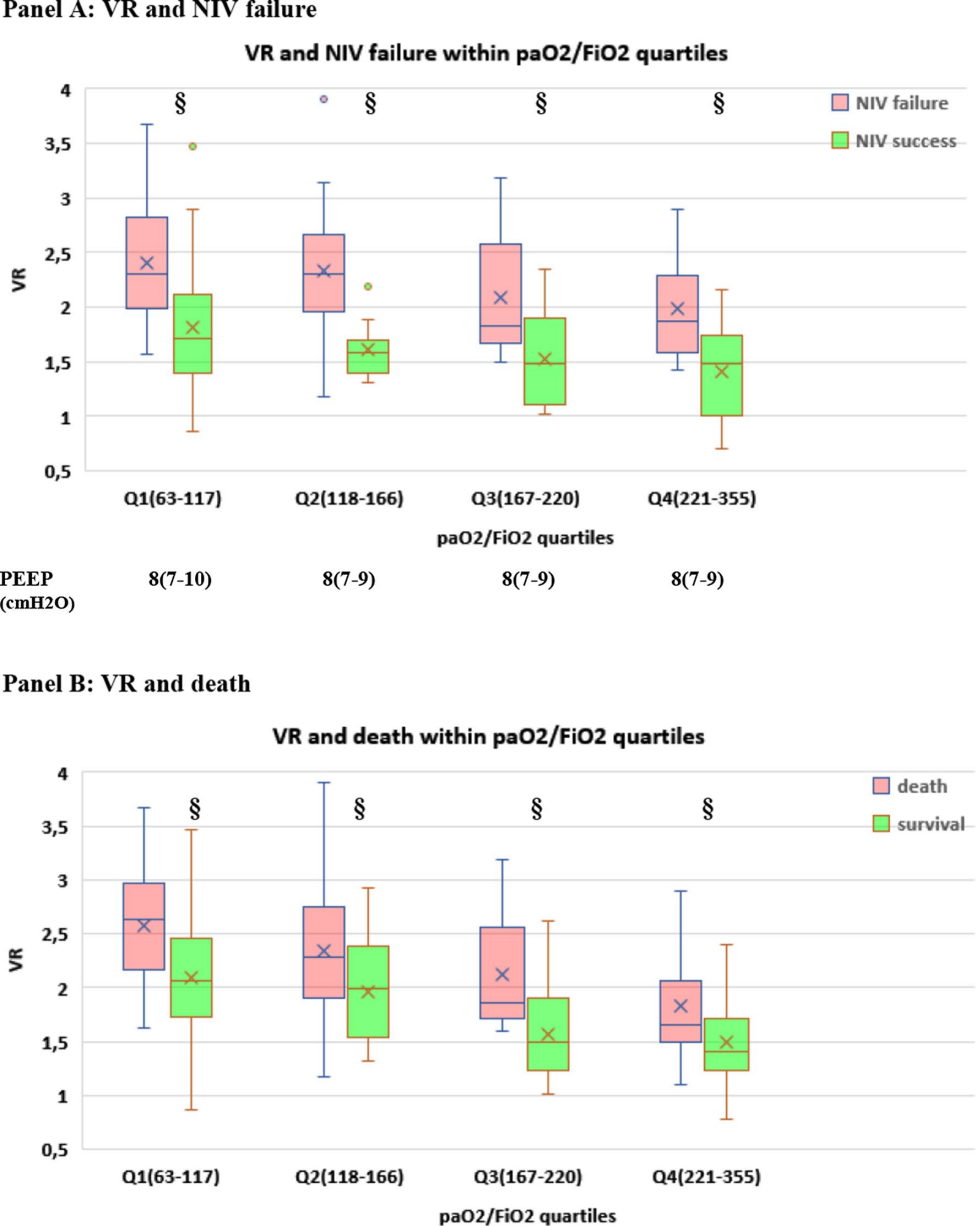

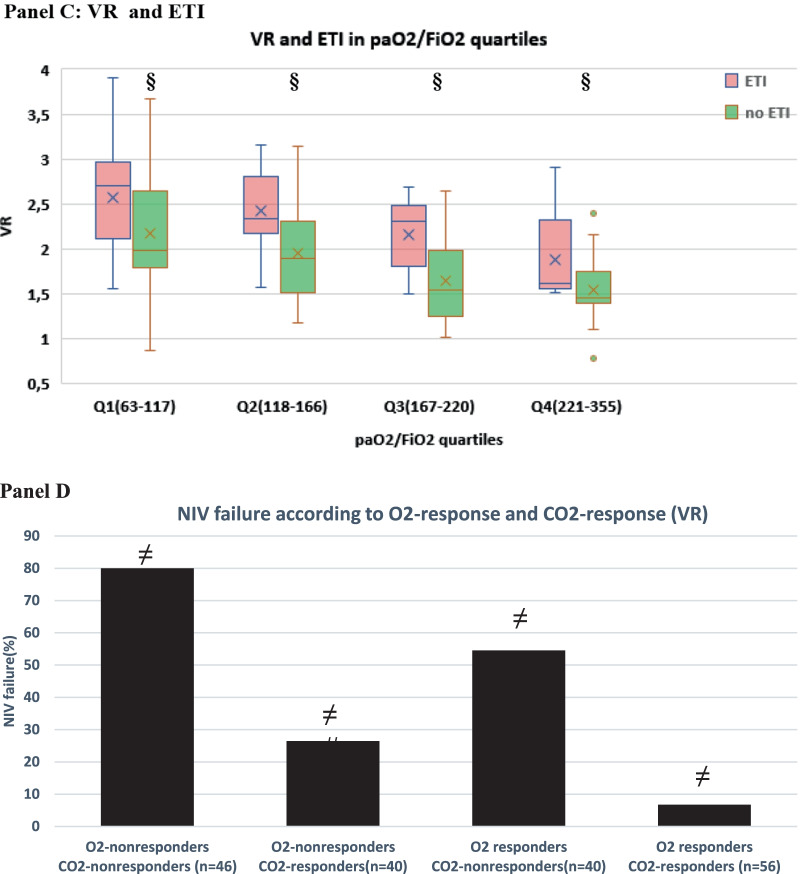

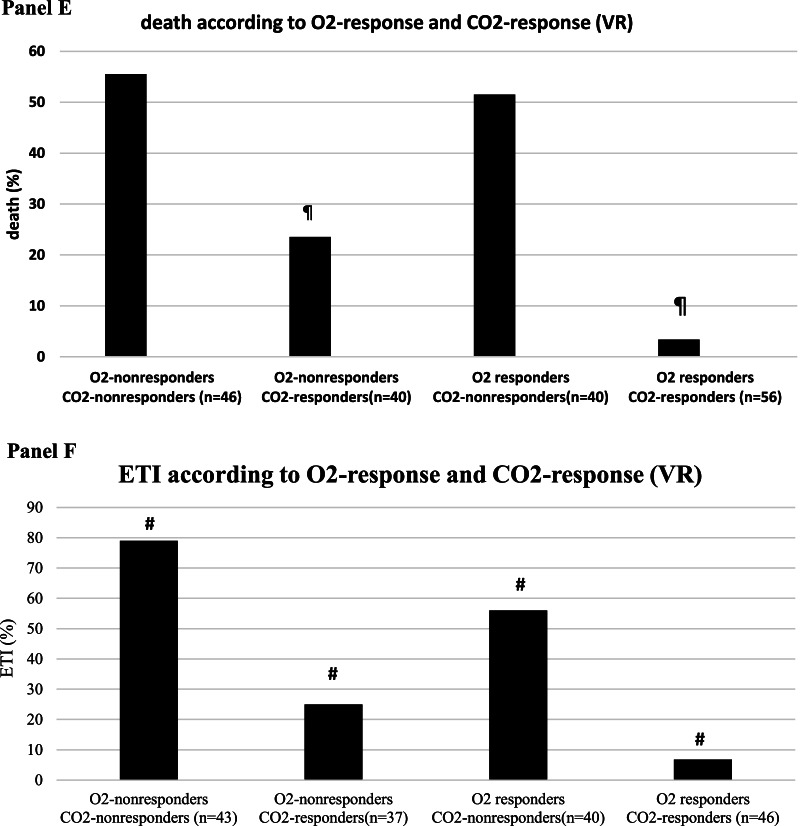
Relationship between gas exchange parameters at day 1 and clinical outcomes (NIV failure, death, endotracheal intubation, ETI) (*n* = 182). **A–C** Relationship between dead space index Ventilatory Ratio (VR) and NIV failure, death and ETI within each paO2/FiO2 quartile at timepoint sp1 (i.e., supine position at day 1, corresponding in the PP group to resupination after the first 8-h overnight PP session). paO2/FiO2 range within each quartile and PEEP (median, IQR) at which measurements were made are reported at the bottom of the panels. §*p* < 0.001 versus treatment failure within quartile. **D–F** Simultaneous impact of O2 response and CO2 response on NIV failure, death and ETI. CO2 response was assessed via ventilatory ratio (VR). *p < 0.05 versus other groups #*p* < 0.01 versus other groups ¶*p* < 0.001 versus other groups

To gain insight into the clinical impact of early changes in oxygenation and dead space, we first explored the relationship between 28-day clinical outcomes (NIV failure, death, ETI), paO2/FiO2 and dead space indices at timepoint sp1: within each paO2/FiO2 quartile, a favorable clinical outcome was associated with lower dead space (as assessed by either VR and MVcorr) (Fig. 5A–C, Additional file 1: Figure S7**)**.

### CO2 response predicts clinical outcomes independently of O2 response

When categorizing the physiological substudy population (*n* = 182) based on O2 response and CO2 response at day 1, CO2 nonresponders had a twofold higher rate of NIV failure, death and ETI than CO2 responders, regardless of O2 response (Fig. 5D–F).

In a Cox multivariable regression model including also gas exchange responses and PEEP at timepoint sp1, CO2 response and O2 response independently predicted NIV failure and ETI, but only CO2 response predicted death (Table 3).

**Table 3:** Cox multivariable analysis of predictors of 28-day NIV failure, death and endotracheal intubation (ETI), after inclusion of gas exchange responses and PEEP at day 1 in supine position (timepoint sp1). CO2 response was assessed via ventilatory ratio (VR) (*n* = 182)

Parameter	HR	95% CI	*p*
*NIV failure*			
Age	1.0241	0.9969–1.0520	0.084
SAPS II	1.0324	0.9886–1.0782	0.151
PP therapy	0.2538	0.1401–0.4597	**< 0.001**
paO2/FiO2 at admission	0.9985	0.8731–1.1014	0.282
RR at admission	0.9621	0.9274–1.1981	0.118
O2 response	0.5901	0.3644–0.9557	**0.032**
CO2 response (VR)	0.2537	0.1483–0.4341	**< 0.001**
PEEP (day 1)	0.9484	0.8414–1.0691	0.388
*Death*			
Age	1.0581	1.0257–1.0915	**0.004**
Type 2 DM	0.9894	0.5432–1.8019	0.971
ISARIC 4C score	1.0597	0.9427–1.1912	0.334
PP therapy	0.2329	0.1127–0.4813	**< 0.001**
paO2/FiO2 at admission	0.9862	0.9420–1.2004	0.286
O2 responder	0.6490	0.3679–1.1448	0.137
CO2 responder (VR)	0.2755	0.0486–0.5107	**< 0.001**
PEEP (day 1)	1.0709	0.9354–1.2261	0.333
*Endotracheal intubation*^a^			
PP therapy	0.1938	0.0870–0.4317	**< 0.001**
pO2/FiO2 at admission	0.9929	0.9873–1.0986	0.132
RR at admission	0.9618	0.9260–1.1096	0.108
O2 responder	0.4623	0.2399–0.8906	**0.022**
CO2 responder (VR)	0.1991	0.1008–0.3934	**< 0.001**
PEEP (day 1)	0.9509	0.7196–1.1063	0.164

^a^For those with a full-treatment indication (*n* = 166)

ISARIC, International Severe Acute Respiratory Infection Consortium; NIV, noninvasive ventilation; SAPS, Simplified acute physiology score

Statistically significant differences are written in bold character

Replacing VR with MV_corr_ yielded similar results (Additional file 1: Figure S8**,** Additional file 2: Table S9).

### Predictors of LUS reaeration and recruitment

In a linear multivariable model, early (day 1) CO2 response, but not PEEP or O2 response, predicted LUS reaeration and recruitment at day 5 (Table 4).

**Table 4:** Multivariable linear regression analysis of early (day 1) predictors of 5-day changes in LUS indices. Changes in pO2/FiO2 and in dead space indices between timepoint sp1 and sp0 (sp0-1) were included in the analysis (*n* = 187). CO2 response was assessed by using either VR (panel A) or MVcorr (panel B)

Parameter	*r*	SE	*p*	VIF	*R*^2^	*R*^2^_adj_
Panel A						
***Δ Global LUS***						
Initial LUS	− 0.51		**< 0.001**	0.05	0.66	0.65
CO2 responder (VR sp0-1)	− 0.39		**0.009**	1.44		
Not included in the model						
O2 responder (pO2/FiO2 sp0-1)	− 0.19		0.391	1.22		
PEEP (day 1)	− 0.13		0.151	1.51		
***Δ global N-consolidations***						
Initial N-consolidations	− 0.51		**0.001**	0.18	0.68	0.67
CO2 responder (VR sp0-1)	− 0.45		**0.001**	1.41		
Not included in the model						
O2 responder (pO2/FiO2 sp0-1)	− 0.21		0.29	1.21		
PEEP (day 1)	0.34		0.16	1.30		
***Global reaeration score***						
Initial LUS	0.55		**0.001**	0.09	0.65	0.64
CO2 responder (VR sp0-1)	0.44		**0.009**	1.37		
Not included in the model						
PEEP (day 1)	0.29		0.138	1.29	0.65	0.64
O2 responder (pO2/FiO2 sp0-1)	0.16		0.479	1.13		

Panel B						
***Δ Global LUS***						
CO2 responder (MVcorr sp0-1)	− 0.39		**0.02**	1.47	0.64	0.62
Initial LUS	− 0.51		**< 0.001**	1.31		
Not included in the model						
O2 responder (pO2/FiO2 sp0-1)	− 0.19		0.41	1.06		
PEEP (day 1)	0.24		0.16	1.08		
***Δ global N-consolidations***						
CO2 responder (MVcorr sp0-1)	− 0.36		**0.010**	1.50	0.68	0.67
Initial N-consolidations	− 0.51		**0.002**	1.30		
Not included in the model						
O2 responder (pO2/FiO2 sp0-1)	− 0.21		0.390	1.05		
PEEP (day 1)	0.31		0.091	1.28		
***Global reaeration score***						
CO2 responder (MVcorr sp0-1)	0.39		**0.009**	1.45	0.64	0.63
Initial LUS	0.50		**0.001**	1.27		
Not included in the model						
O2 responder (pO2/FiO2sp0-1)	0.16		0.381	1.04		
PEEP (day 1)	0.29		0.253	1.13		

Δ global LUS score, global Lung Ultrasonographic Score (LUS) (day 5) + global LUS (day 0); Δ global N-consolidations, global N-areas with consolidations (i.e., score 3) at day 5 − global N-areas with consolidations (i.e., score 3) at day 0; MVcorr, corrected Minute Ventilation; PEEP, positive end-expiratory pressure; *r*, partial correlation coefficient; *R*^2^, coefficient of determination; *R*^2^ adj, adjusted *R*^2^; SE, standard error; VIF, variance inflation factor; VR, ventilatory ratio. Statistically significant associations are written in bold characters

### Dose–response relationship between day 1 h of PP therapy, gas exchange and LUS indices

**Fig. 6:**
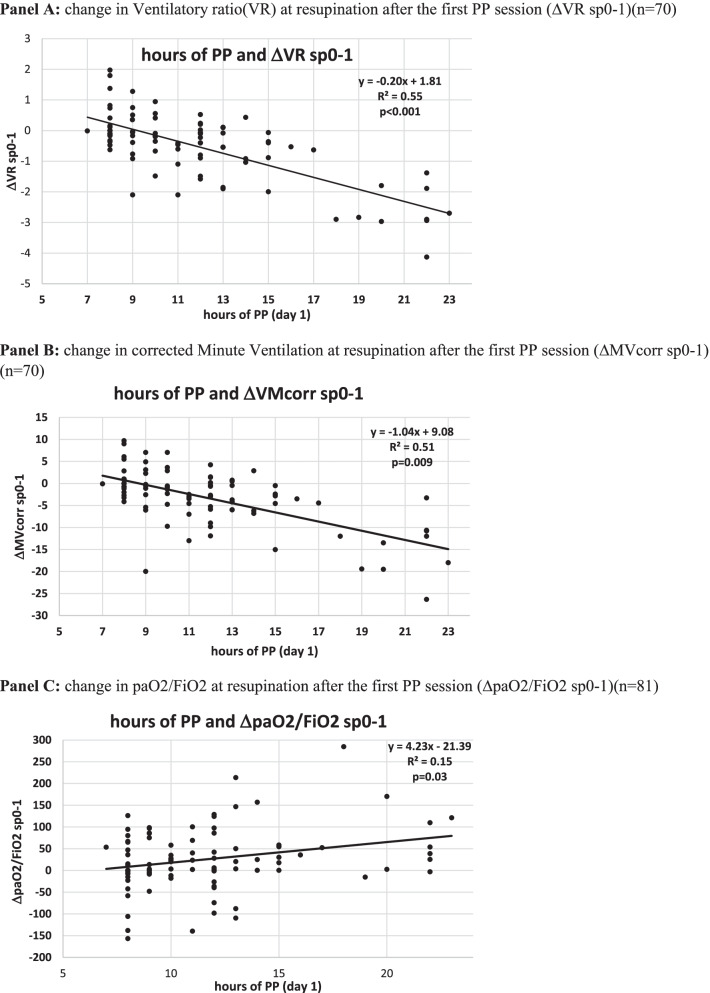

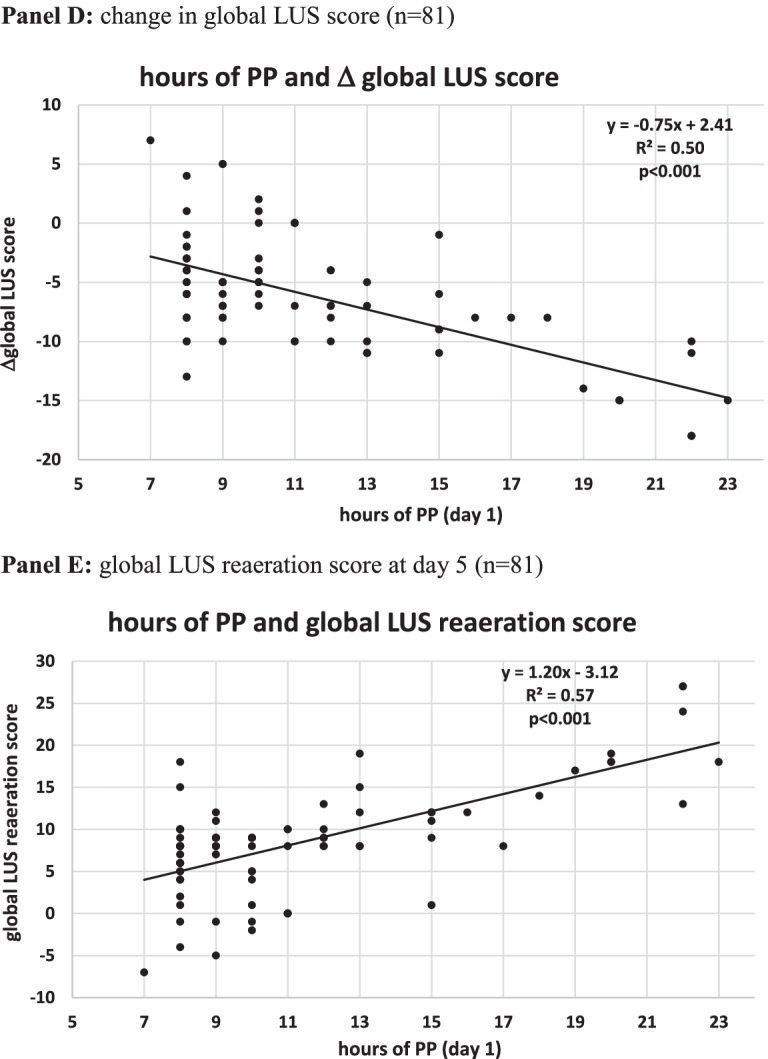
Dose–response relationship between hours of individual PP sessions (day 1) and changes in gas exchange and ultrasonographic indices in PP patients. **A** Change in ventilatory ratio (VR) at resupination after the first PP session (VR sp0-1) (*n* = 70). **B** Change in corrected minute ventilation at resupination after the first PP session (MVcorr sp0-1) (*n* = 70). **C** Change in paO2/FiO2 at resupination after the first PP session (paO2/FiO2 sp0-1) (*n* = 81). **D** Change in global LUS score (*n* = 81). **E** Global LUS reaeration score at day 5 (*n* = 81). ABG, arterial blood gas analysis; BC, blood chemistry; LUS, lung ultrasound; PP, prone position; pp1: during the first PP session in the PP group; pp2: during the second PP session; SUP: supine position: sp0: supine position timepoint 0 (baseline, after NIV initiation). sp1: supine position timepoint 1 (after the initial 24 h of NIV; in the PP group this also corresponded to the resupination after the first PP session on day 1); sp2: supine position timepoint 2

The length of PP sessions at day 1 predicted over 50% of variation in DSIs (ΔVRsp0-1 and ΔMVcorr sp0-1) and in LUS reaeration and recruitment, while the ability to predict paO2/FiO2 changes was lower (Fig. 6).

The regression line indicated that an improvement in paO2/FiO2 ratio (ΔpaO2/FiO2 0–1 > 0) was observed in patients proning for at least 6 h, a reduction in dead space (i.e., ΔVRsp0-1 and ΔMVcorr sp0-1 < 0) was observed in patients proning for at least 9 h, and a global LUS reaeration score ≥ 8 was observed in patients proning for at least 10 h (Fig. 6).

### Biomarker changes over the initial 7 days

Five biomarkers of COVID-19 disease severity and mortality [22, 39] differed significantly between PP group and controls: C-reactive protein (CRP), D-dimer, LDH and neutrophil-to-lymphocyte ratio (NLR) fell significantly, while lymphocyte count increased significantly in the PP group as compared with controls (Fig. 4E–I).

The earliest changes were observed with serum CRP levels, which fell significantly after the first PP session, while the difference in the other biomarkers became significant at day 4.

In multivariable regression analysis, CO2 response (day 1) and LUS reaeration score independently predicted CRP, D-dimer and NLR changes over the initial 7 days (Additional file 2: Table S10).

### Other prespecified secondary and post hoc analyses (Additional file 2: Tables S11–S18)

In the prespecified per-protocol analysis that excluded patients with PP failure and rescue PP, PP therapy remained significantly associated with a reduced risk of NIV failure, ETI, and death.

Baseline patient demographics, clinical features, and adjunctive therapies did not differ between controls from the first and from the second pandemic wave (Additional file 2: Table S16).

Results of other prespecified secondary analyses substantially confirmed the findings from the primary analysis**.** Additionally, after excluding patients with documented bacterial or mycotic infection, serum procalcitonin differed significantly between PP group and controls (Additional file 1: Figure S9).

## Discussion

Main findings of our study are the following:

1. In COVID19-related moderate-to-severe acute hypoxemic respiratory failure, early (i.e., within 24 h of admission) prolonged (i.e., ≥ 8 h/day) PP combined with NIV was associated with a significant reduction in treatment failure, mortality ,and intubation rate as compared to conventional (supine) NIV.
2. Compared with supine NIV, NIV delivered in PP was associated with enhanced lung reaeration and recruitment and with regression of dorso-lateral lung consolidations.
3. In the whole study population, dead space reduction and enhanced CO2 clearance at day 1 predicted lung reaeration, treatment success and survival, outperforming oxygenation indices.
4. Ventilatory and ultrasonographic changes were coupled with a quicker decrease in circulating proinflammatory and procoagulative biomarkers and with normalization of circulating leucocyte subpopulations in the PP group as compared with controls

In noninvasively ventilated COVID-19 patients with moderate-to-severe hypoxemic respiratory failure, early PP was safe and effective as compared with a group of historical controls, matched by PS for relevant baseline and treatment-related parameters.

Several factors may have contributed to the benefits observed with our PP strategy, which was early and prolonged. The critical role of early initiation of PP is highlighted by experimental data, suggesting the effects of PP on lung aeration depend on the stage of lung injury and on the duration of ventilator-induced injury (VILI) during supine NIV, which is attenuated and redistributed by PP [40, 41]; furthermore, in a recent post hoc analysis of HFNC patients, early PP therapy was associated with a 50% mortality reduction as compared with late proning [42].

The minimal duration of individual PP sessions was set at eight consecutive hrs for 2 reasons: first, because ARDS data suggest clinical benefit from long uninterrupted PP sessions [6]; second, to avoid selection bias, whereby sicker, older patients who are more liable to treatment failure are also unable to prone longer [14]. Furthermore, total daily hours of PP therapy were not set a priori, but flexible and dictated by individual oxygenation and RR responses at resupination after individual PP sessions, with patients resuming PP unless meeting weaning goals from PP therapy. Hence daily dose of PP therapy was substantial and commensurate with individual patient respiratory distress severity. Regarding the minimal required duration of individual PP session, regression plots show that while 6 h/day of PP were associated with O2 response, CO2 response was associated with ≥ 9 h/day of PP and a LUS reaeration score ≥ 8 was associated with 10 h/day of PP at day 1 (Fig. 6).

Regarding the minimal days of PP therapy, the integration of daily course of RR, dead space indices, blood biomarkers, and LUS findings indicates that at least 4 days of PP therapy would be required to observe a stable reduction in RR and dead space indices, an improvement in biomarkers of prothrombotic and immune cell dysregulation and ultrasonographic lung reaeration (Figs. 3, 4).

We next explored the association of observed changes in gas exchange and ventilatory parameters with radiological and clinical outcomes.

The comparison of ventilatory parameters between PP and controls and between treatment failures and responders indicates that lower ventilatory volumes, which are hardly achievable in noninvasively ventilated patients with acute hypoxemia respiratory failure [43], were not associated with outcomes of PP (Additional file 2: Tables S3–S6).

Rather, dead space reduction and enhanced CO2 clearance at day 1 predicted LUS reaeration, inflammatory biomarkers decline, and a favorable clinical outcome in the whole study population and outperformed oxygenation indices in death prediction in multivariable models (Table 4; Additional file 2: Tables S3, S5, S9).

These findings are consistent with recent reports in invasively ventilated COVID-19 ARDS [44] and warrant interpretation.

Dead space indices in COVID 19 may reflect a combination of hypoperfused alveoli due to microthrombosis of capillary alveoli and/or interstitial edema and alveolar fluid accumulation impairing CO2 elimination [45]. We did not find any relationship between day 1 changes in DSI and plasma D-dimer, whose levels decreased at later days **(**Fig. 4F). Rather, the prompt DSI reduction at day 1 moderately correlated with PP hours (Fig. 6), suggesting PP may contribute to more homogeneous lung inflation and tidal volume distribution, and enhance aeration and recruitment of consolidated dorso-lateral lung regions as compared with supine position.

The higher CO2 response rate and lung recruitability of our patients as compared with invasively ventilated patients with COVID-19-related ARDS [35] suggests severe parenchymal abnormalities are reversible at earlier disease stages and highlights the need to early identify patients who might benefit from PP therapy.

From a practical standpoint, dead space indices may represent a more useful tool than oxygenation indices to monitor NIV adequacy and select patients for lung protective PP: CO2 response 24 h after supine NIV initiation may indicate the need for PP therapy to enhance dependent lung recruitment, while absent CO2 response after PP initiation may herald NIV failure.

Notably, dead space and lung aeration responses paralleled and predicted a faster dampening of systemic proinflammatory and procoagulative cascade biomarkers and a trend to normalization in circulating immune cell profile **(**Fig. 4, Additional file 2: Table S10). While observational data relate these biomarkers to oxygenation and respiratory mechanics impairment in COVID-19-related ARDS [27, 46], a rapid normalization in these proinflammatory biomarkers during the initial 2 weeks of COVID-19 predicted full recovery without pulmonary fibrosis [39, 47, 48].

Future RCTs need to evaluate if PP therapy may contribute to attenuate NIV-associated lung injury [41] and to limit pulmonary fibrotic sequelae in these patients. The impact of timing of initiation of PP therapy on physiological and clinical outcomes in noninvasively ventilated COVID-19 patients needs also to be assessed.

While the thorough and prolonged integration of ventilatory, ultrasonographic, and biochemical parameters provides novel pathophysiological and clinical insights, limitations of this study deserve mention.

We tried to obviate the lack of randomization by PS matching of PP and controls for known baseline and treatment-related confounders. Even so, unknown confounders may still exist, and the natural drift in disease severity and mortality may have contributed to the observed clinical benefits, as the PP group was enrolled during the 2–3rd wave (Dec 2020–May 2021) and the controls belonged to the 1st–2nd wave (April 2020–Dec 2020). However, restricting the comparison of PP patients with controls belonging to the second wave showed similar baseline and treatment-related characteristics, while the benefits in the PP group remained significant (Additional file 2: Table S16**;** Additional file 1: Figure S5).

Lastly, mortality rate of our PP patients was still considerably lower than that reported for COVID-19-related ARDS patients admitted to European ICUs during the same timeframe, ranging 30–55% [49–51].

Another potential concern with extrapolating dead space indices from invasive to noninvasive ventilation is the impact of dead space and CO2 rebreathing generated by the internal volume of the face mask, which was the interface used for the physiological substudy; however, it has been demonstrated that even with full face masks the dynamic dead space is negligible during ventilation, due to the streaming effect of gas flow during NIV [52, 53].

Furthermore, the monocentric nature mandates caution in generalizability of our findings, which need to be confirmed by randomized trials.

Last, due to the substantial duration of PP therapy a large proportion of patients required continuous infusion of a short-acting, non-respiratory depressant sedative to prone. Although safe in our study, its use requires expertise and close monitoring and prompts development of adequate technical equipment to allow more comfortable patient proning.

## Conclusions

Early prolonged PP therapy during NIV was feasible and was associated with clinical benefits in COVID-19-related moderate-to-severe acute respiratory failure. Integration of ventilatory, ultrasonographic and biochemical parameters provided a pathophysiological frame for observed benefits with PP therapy and individuated useful tools for early prediction of NIV failure. Whether our approach is applicable to other etiologies of hypoxemic respiratory failure characterized by high parenchymal inhomogeneity and dependent lung consolidations warrants future evaluation.

## Supplementary Information

**Additional file 1:** Supplementary Figures.

**Additional file 2:** Supplementary Tables.

**Additional file 3:** Supplementary protocol.

## Data Availability

Individual, de-identified participant data will be shared upon request to the corresponding author by email till up to 2 years after publication of the article.
