# Supplementary material for: Early prolonged prone position in noninvasively ventilated patients with SARS-CoV-2-related moderate-to-severe hypoxemic respiratory failure: clinical outcomes and mechanisms for treatment response in the PRO-NIV study
Source: Crit Care. 2022 Apr 29;26:118. doi: 10.1186/s13054-022-03937-x (PMC9052189; doi:10.1186/s13054-022-03937-x)
Supplement: Supplementary file 1 — Additional file 1: Supplementary Figures. [file 13054_2022_3937_MOESM1_ESM.pdf]

Supplementary Figures

Early prolonged prone position in noninvasively ventilated patients with SARS-CoV-2-related moderate-to-severe hypoxemic respiratory failure: clinical outcomes and mechanisms for treatment response in the PRO-NIV study

Supplementary Figure 1: flow of participants through study

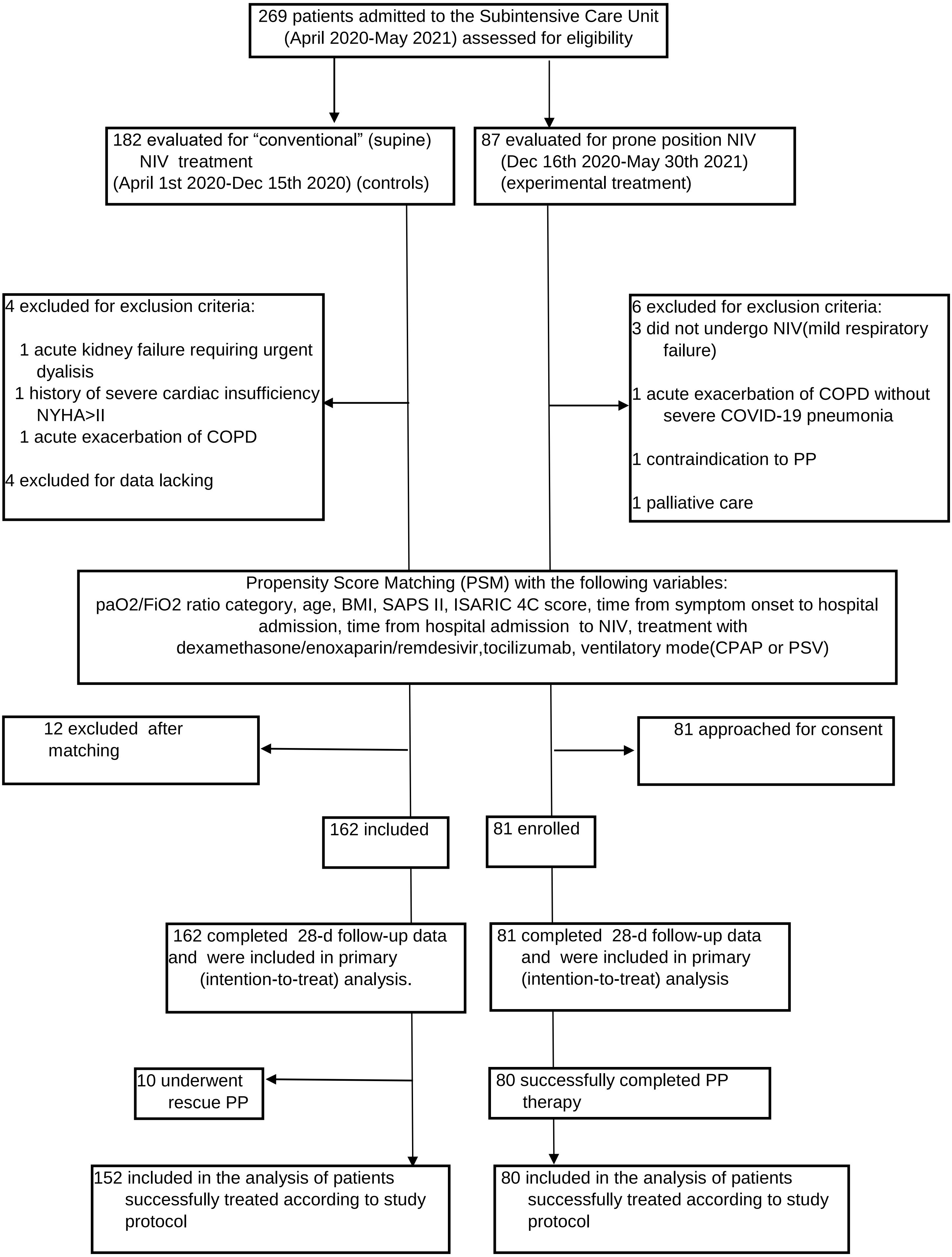

Supplementary Figure 2. Propensity score(PS) density in PP and controls before and after matching (panel A) and balance plots of logit(PS) in PP and controls before and after matching(panel B)

Panel A: Propensity Score Distribution before and after matching

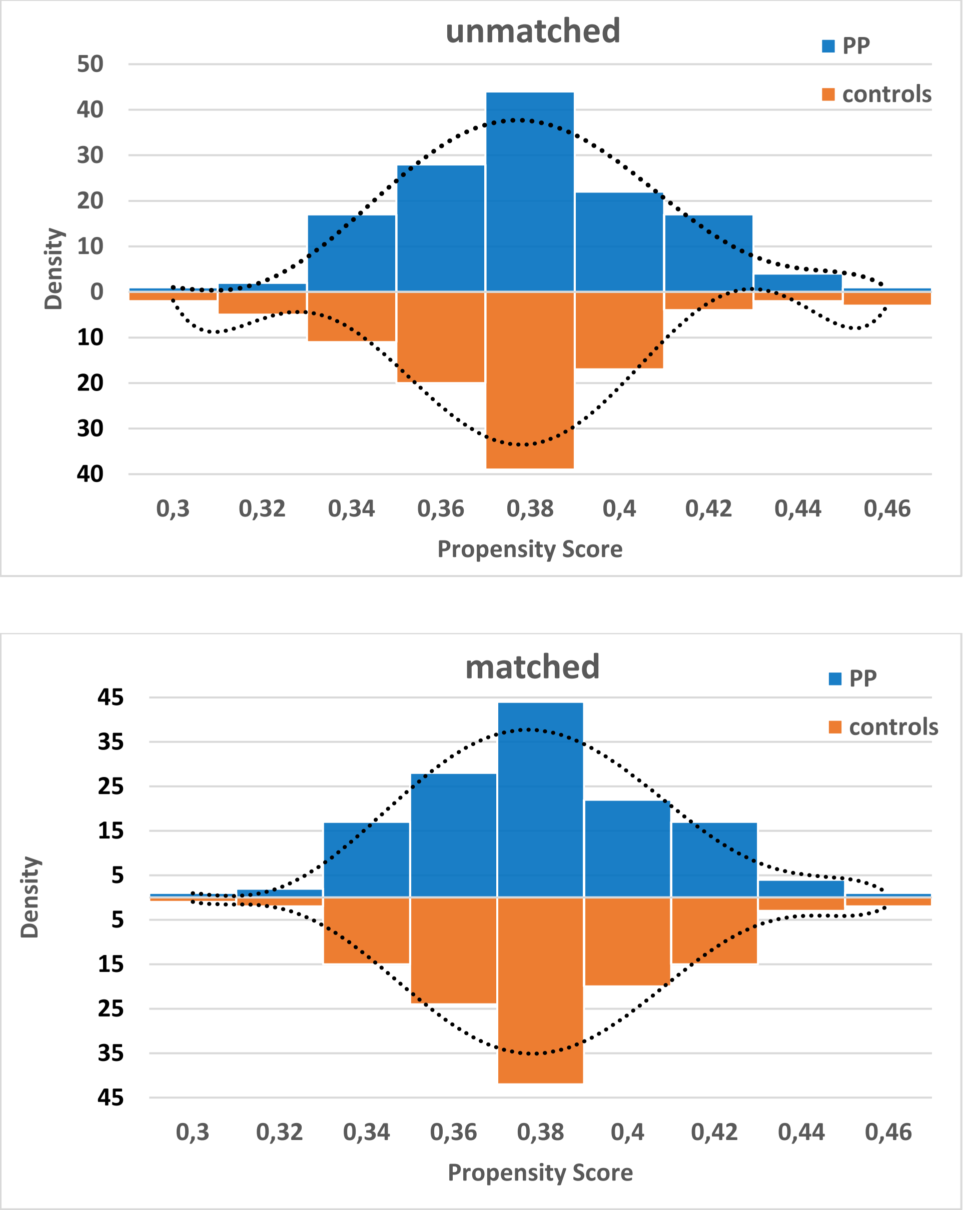

Panel B: balance plots of logit (Propensity Score) in PP and controls before and after matching

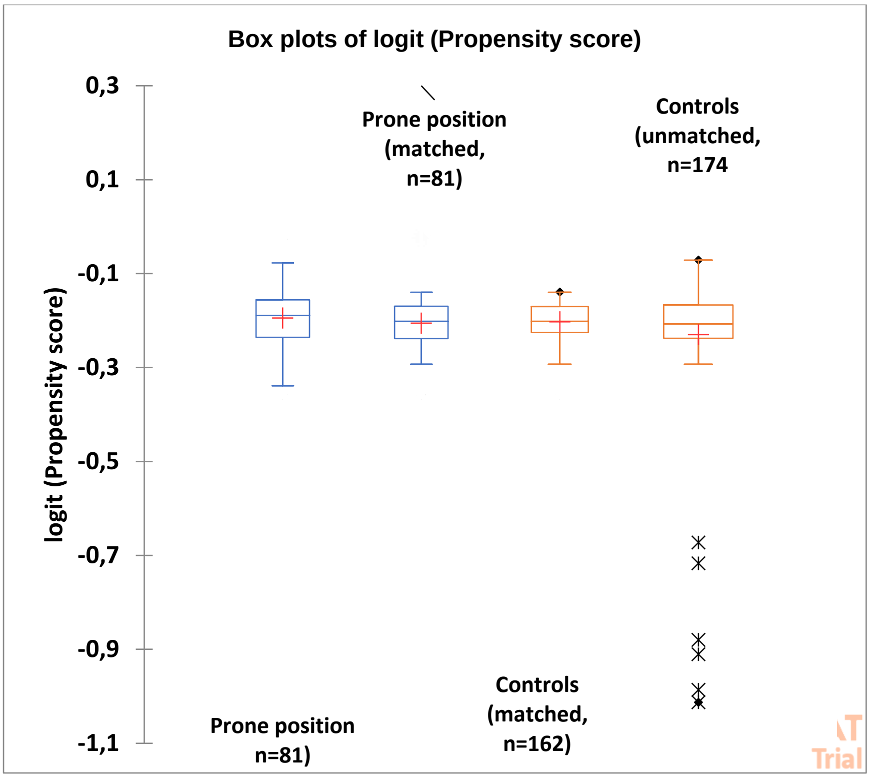

Supplementary Figure 3. Total daily hours of NIV and of NIV in prone position in both groups during the initial 7 study days in the whole study populaton (n=243). Bars represent the median, whiskers the IQR1 and IQR3

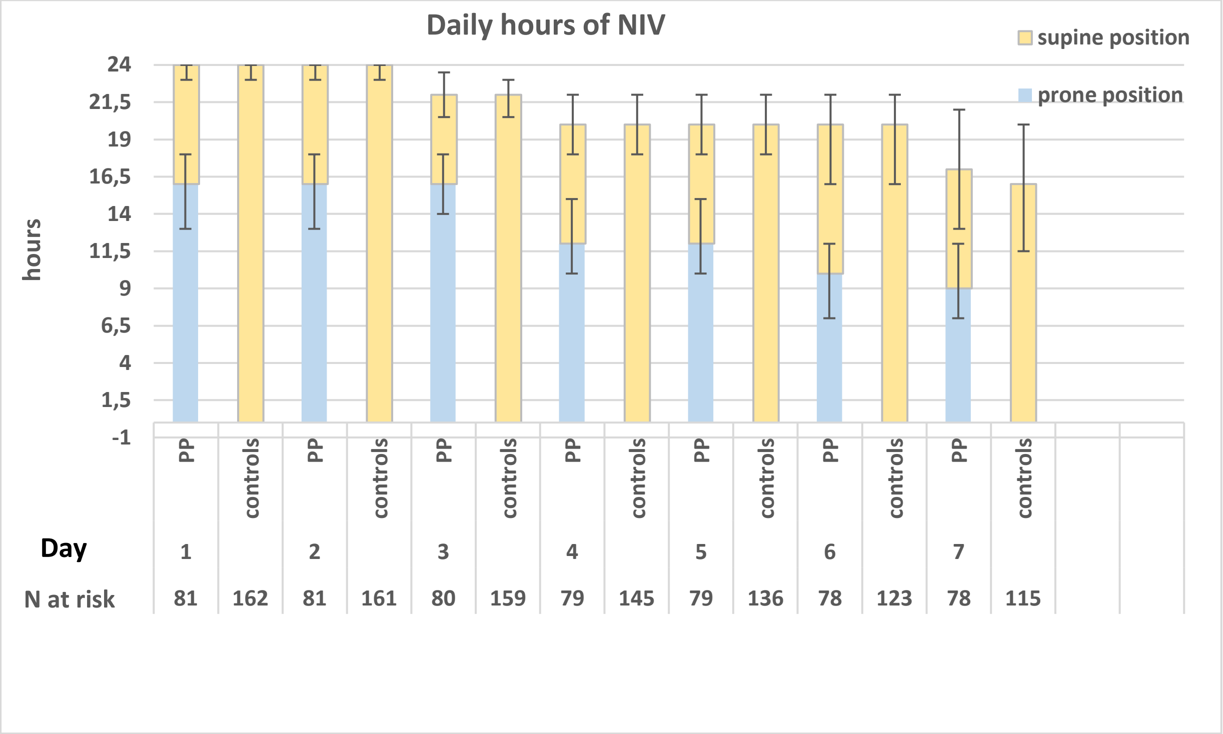

Supplementary Figure 4. NIV failure, death and endotracheal intubation in patients grouped according to paO2/FiO2 at admission: paO2/FiO2<100 vs paO2/FiO2 100-199

Panel A: NIV failure in patients with paO2/FiO2<100

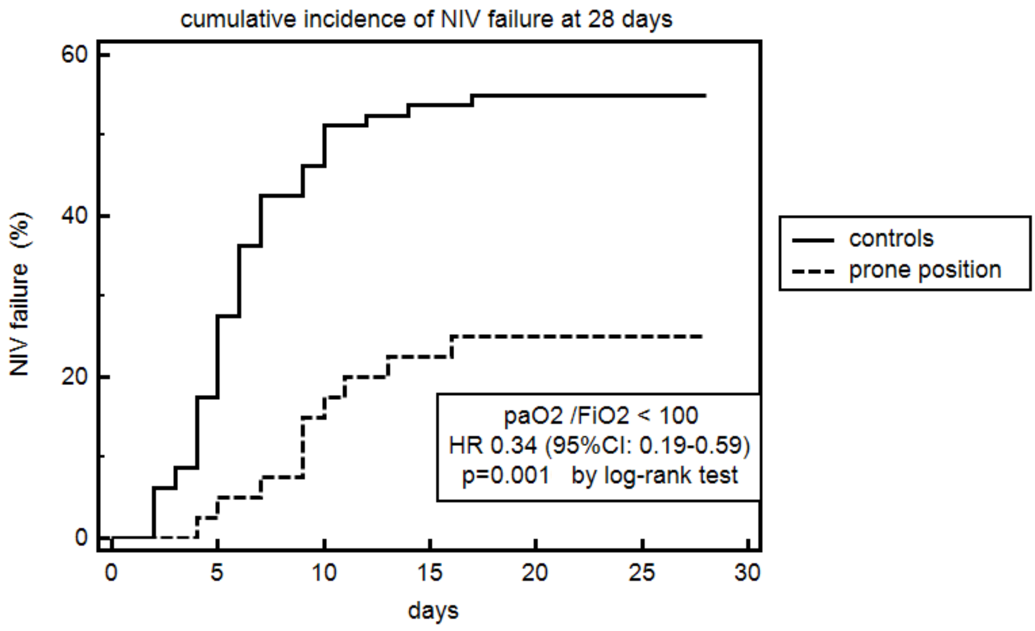

Number at risk

Group: controls							
	80	58	39	37	36	36	0
Group: prone position							
	40	38	33	31	30	30	0

Panel B: NIV failure in patients with paO2/FiO2≥100

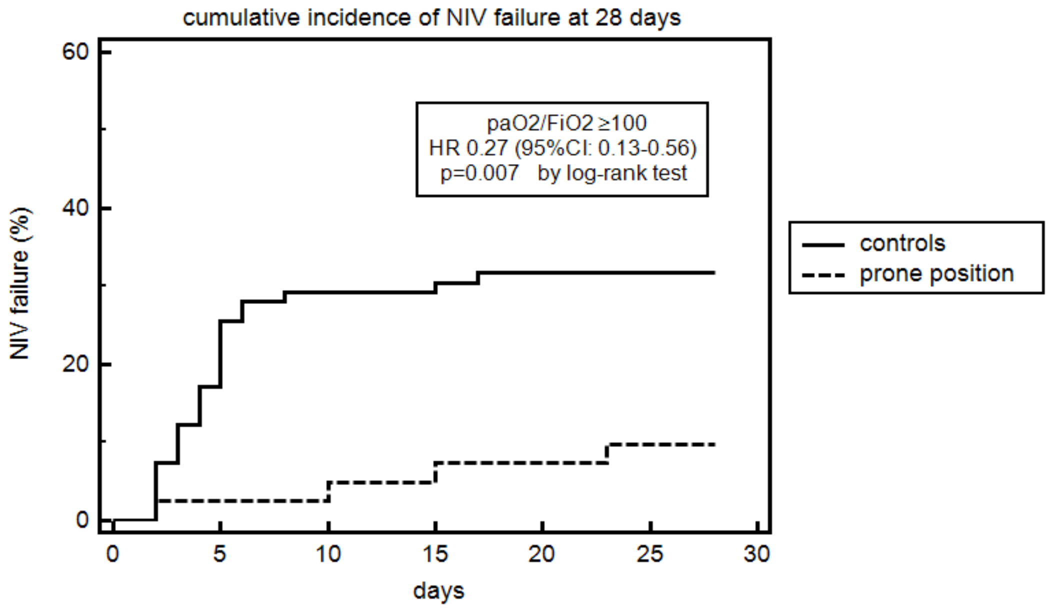

Number at risk

Group: controls							
	82	61	58	57	56	56	0
Group: prone position							
	41	40	39	38	38	37	0

Panel C: death in patients with paO2/FiO2<100

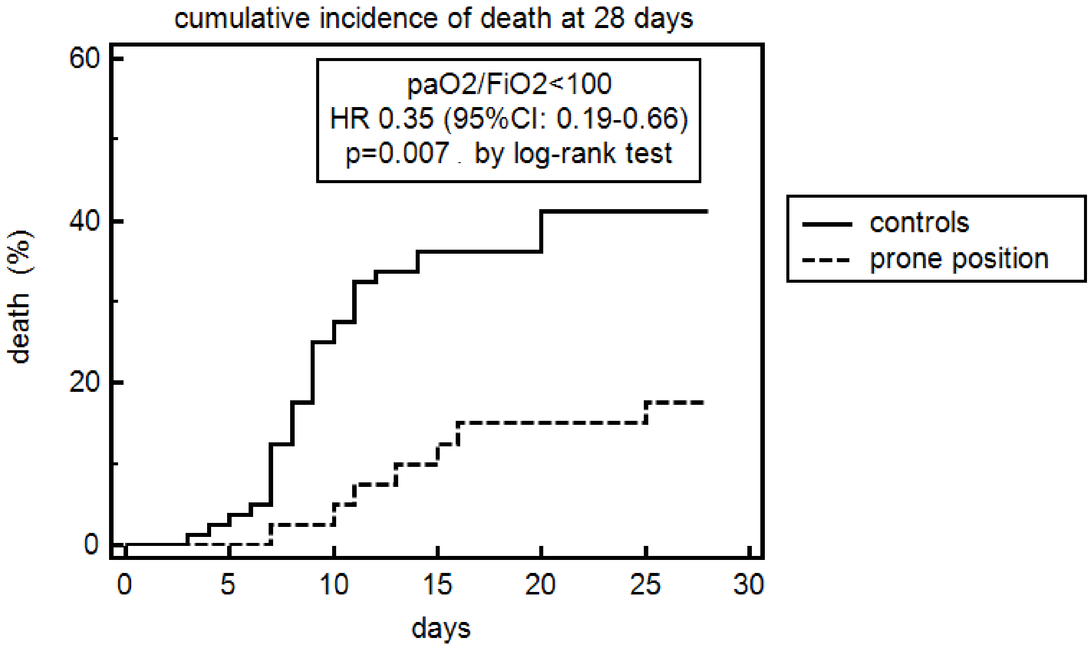

Number at risk

Group	0	5	10	15	20	25	30
Group: controls	80	77	58	51	47	47	0
Group: prone position	40	40	38	35	34	33	0

Panel D: death in patients with paO2/FiO2≥100

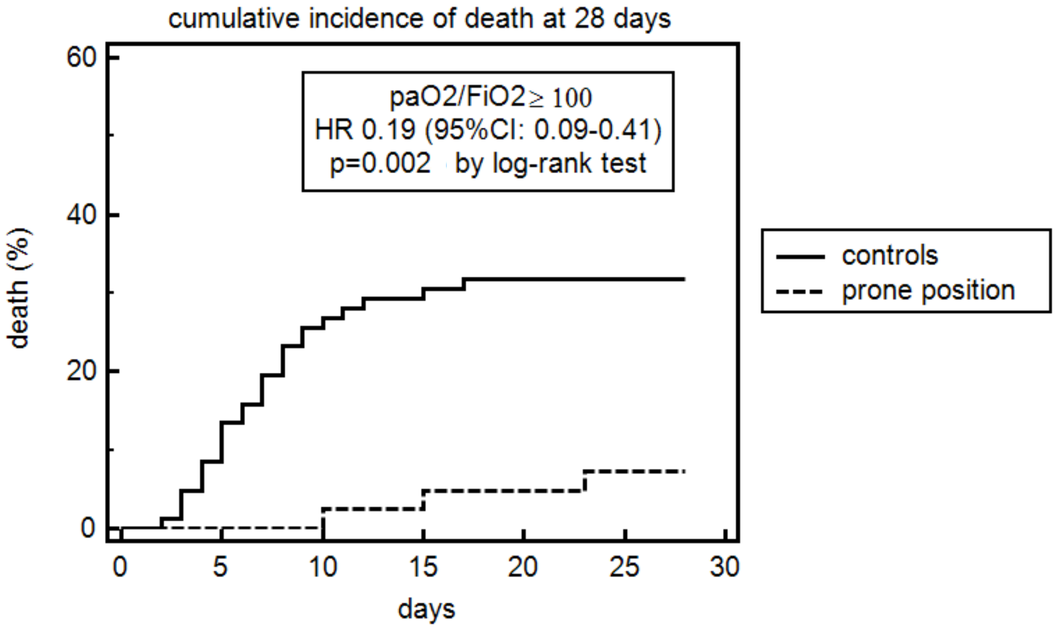

Number at risk

Group	0	5	10	15	20	25	30
Group: controls	82	71	60	57	56	56	0
Group: prone position	41	41	40	39	39	38	0

Panel E: endotracheal intubation in patients with paO2/FiO2<100

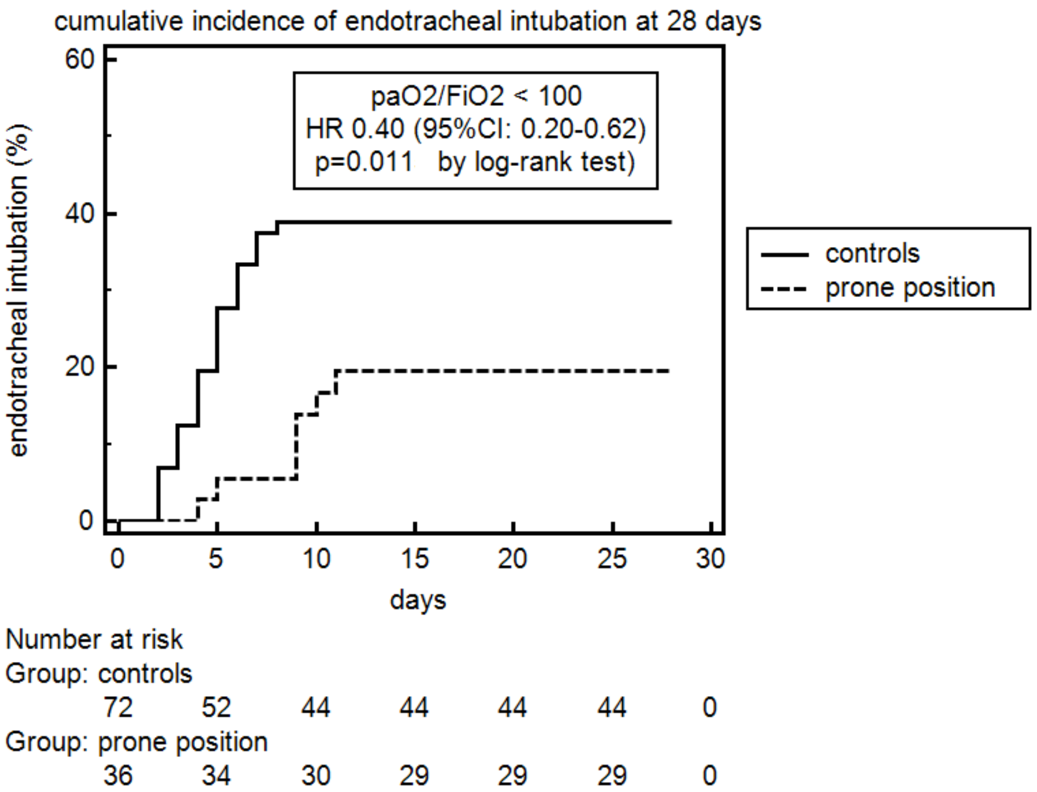

Panel F: endotracheal intubation in patients with paO2/FiO2≥100

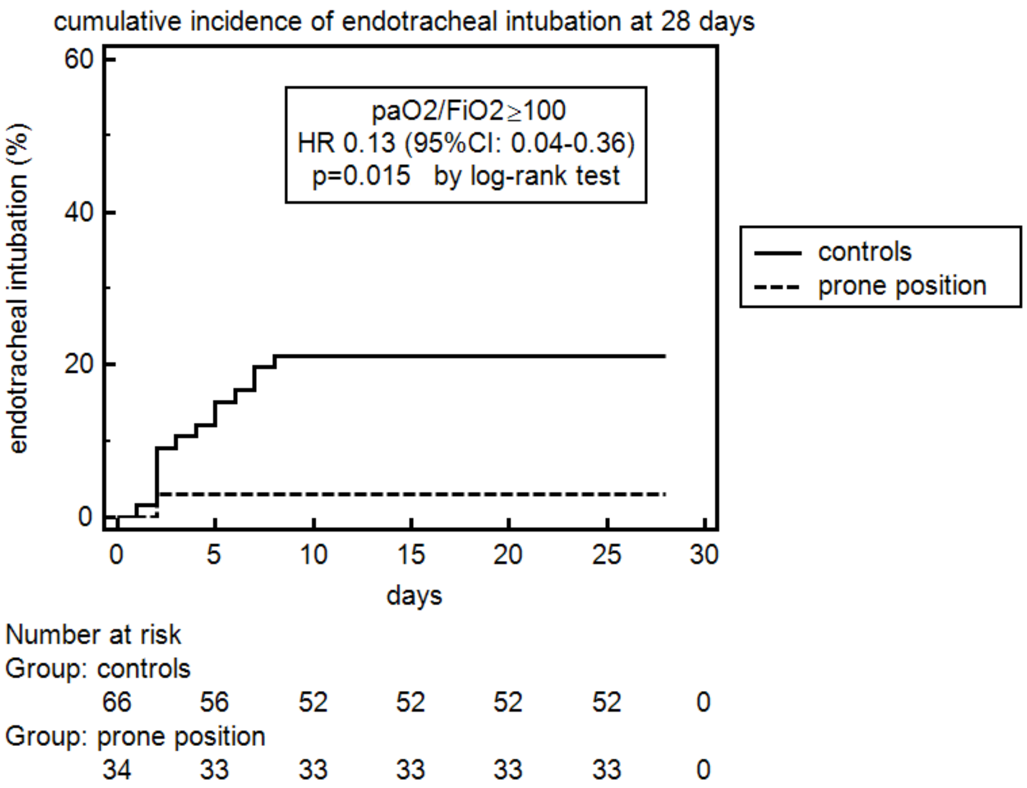

Supplementary Figure 5. NIV failure, death and endotracheal intubation in the prone position group vs. controls enrolled the 1st pandemic wave (March 1st-June 30th, 2020) or the 2nd pandemic wave (July 1st-Dec 15th, 2020).

Panel A: NIV failure (controls from the 1st wave)

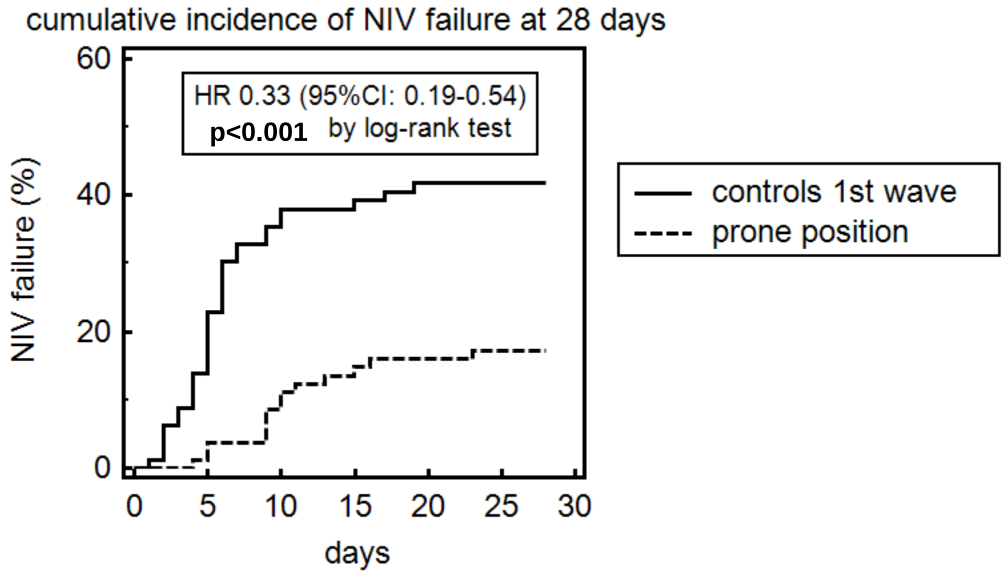

Number at risk

Group: controls 1st wave

79 61 49 48 46 46 0

Group: prone position

81 78 72 69 68 67 0

Panel B: NIV failure (controls from the 2nd wave)

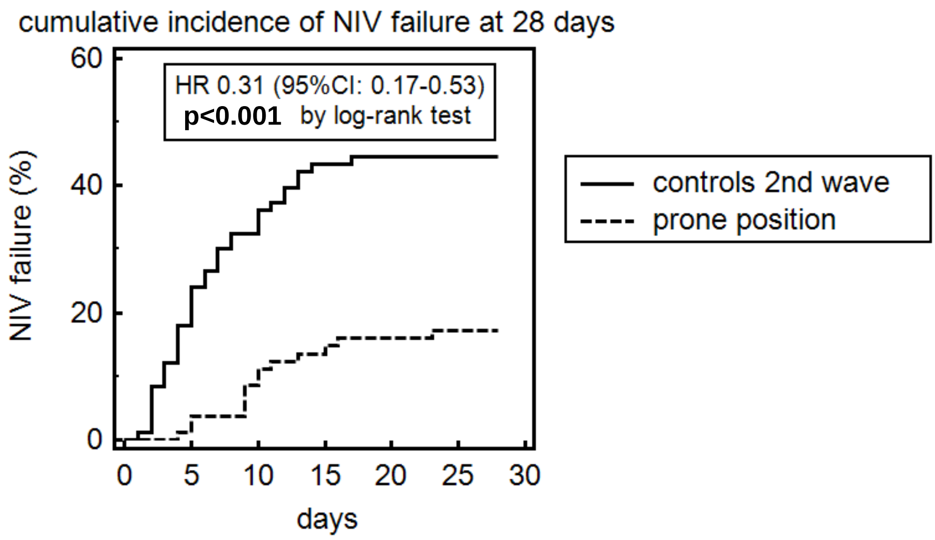

Number at risk

Group: controls 2nd wave

83 63 53 47 46 46 0

Group: prone position

81 78 72 69 68 67 0

Panel C: death (controls from the 1st wave)

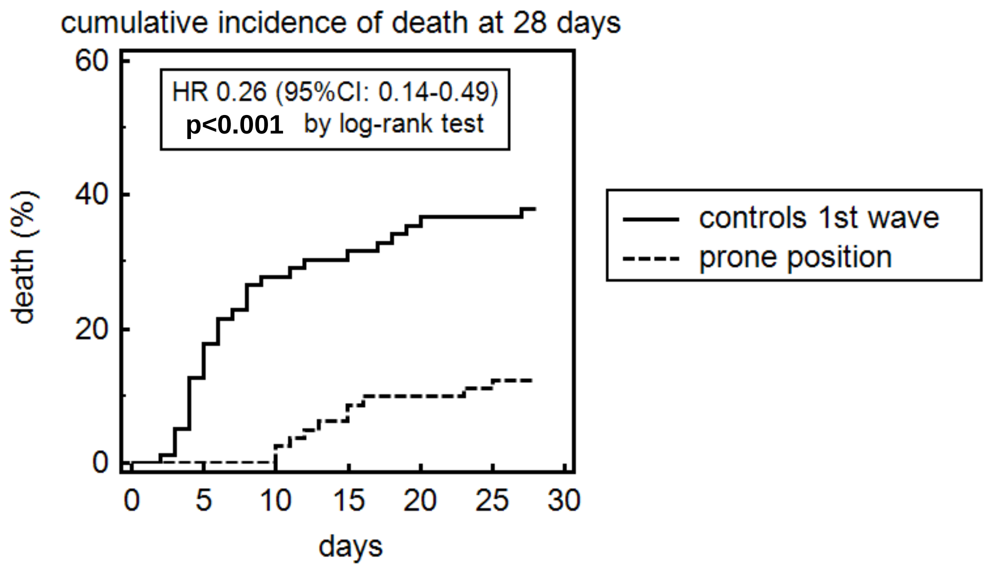

Number at risk

Group: controls 1st wave	79	65	57	54	50	50	0
Group: prone position	81	81	79	74	73	71	0

Panel D: death (controls from the 2nd wave)

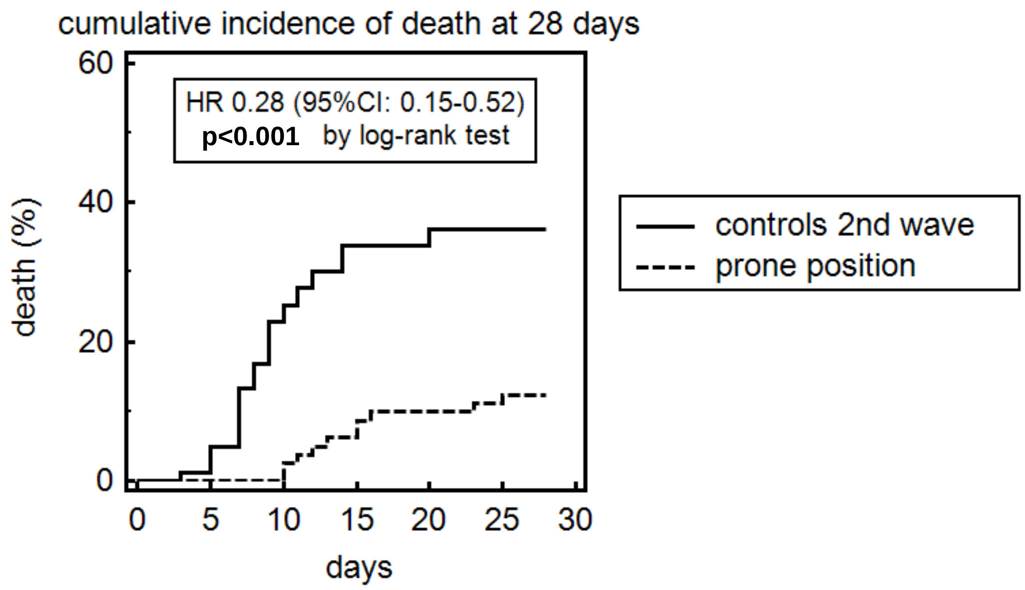

Number at risk

Group: controls 2nd wave	83	79	62	55	53	53	0
Group: prone position	81	81	79	74	73	71	0

Panel E: endotracheal intubation (controls from the 1st wave)

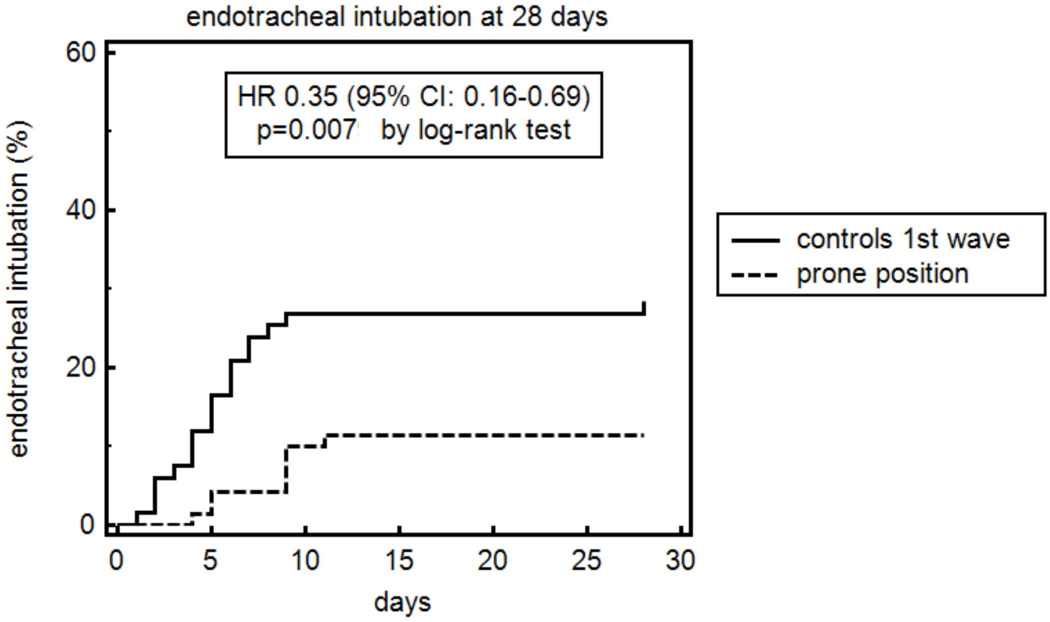

Number at risk
Group: controls 1st wave
67 56 49 49 49 49 0
Group: prone position
70 67 63 62 62 62 0

Panel F: endotracheal intubation (controls from the 2nd wave)

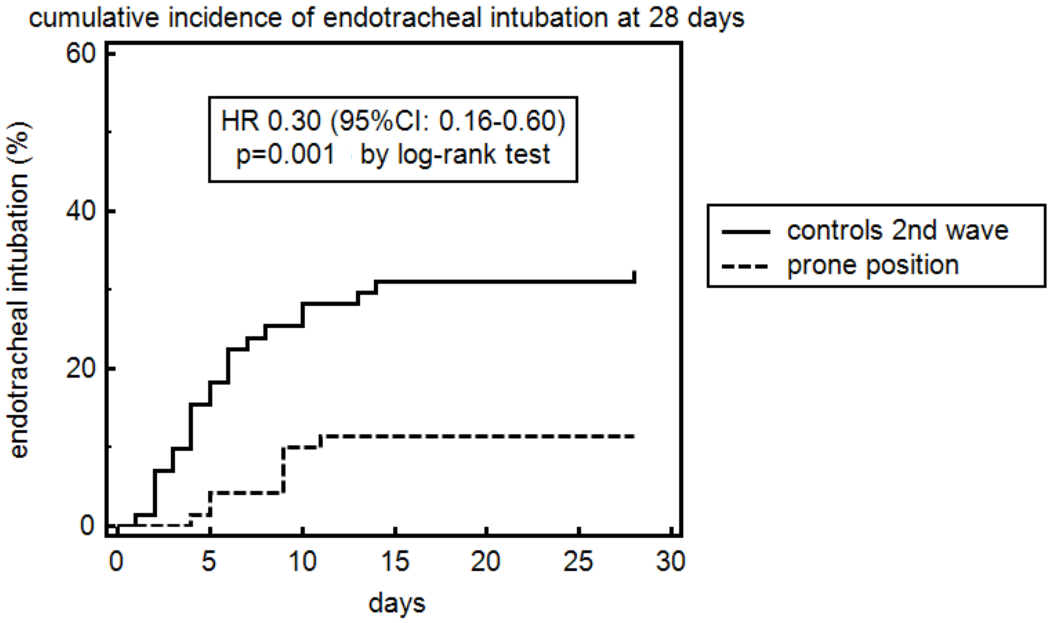

Number at risk
Group: controls 2nd wave
71 58 51 49 49 49 0
Group: prone position
70 67 63 62 62 62 0

Supplementary Figure 6: correlation between global lung ultrasound (LUS) score and global chest computed tomography (CT) severity score, as proposed by Salaffi et al. in 162 patients with SARS-CoV-2-related acute hypoxemic respiratory failure admitted to the Subintensive Care Unit. The median (IQR) CT severity score was 46(32, 60) and the median (IQR) LUS severity score was 25(21, 30). The Spearman correlation coefficient (r_s) with its 95%CI and statistical significance are shown in the Figure.

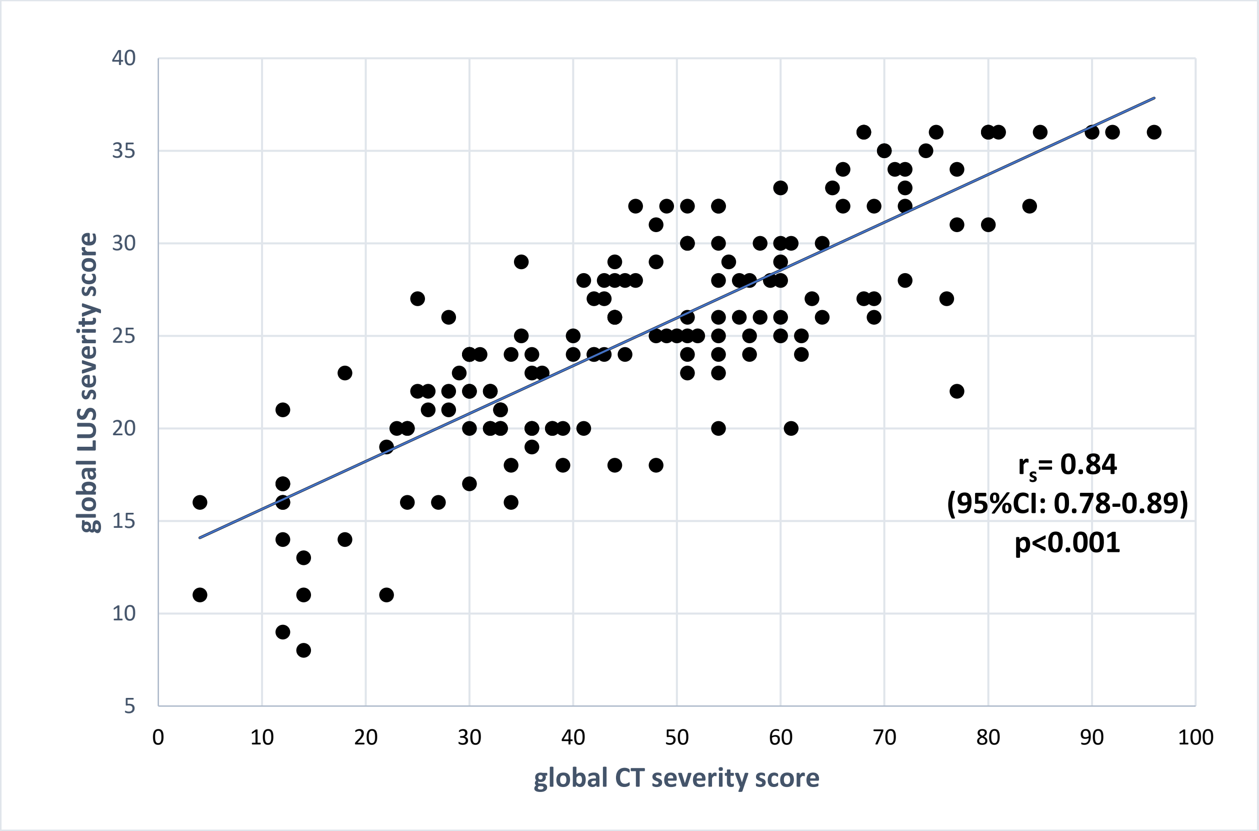

Supplementary Figure 7. Relationship between dead space index corrected Minute Ventilation (MVcorr)] and NIV failure, death and ETI within each paO2/FiO2 quartile at timepoint sp1(i.e. supine position at day 1, corresponding in the PP group to resupination after the first 8-hr overnight PP session)(n=182). paO2/FiO2 range within each quartile and PEEP (median, IQR) at which measurements were made are reported at the bottom of the panels.

* P<0.05 vs. treatment failure within quartile # P<0.001 vs. controls

Panel A: MVcorr and NIV failure

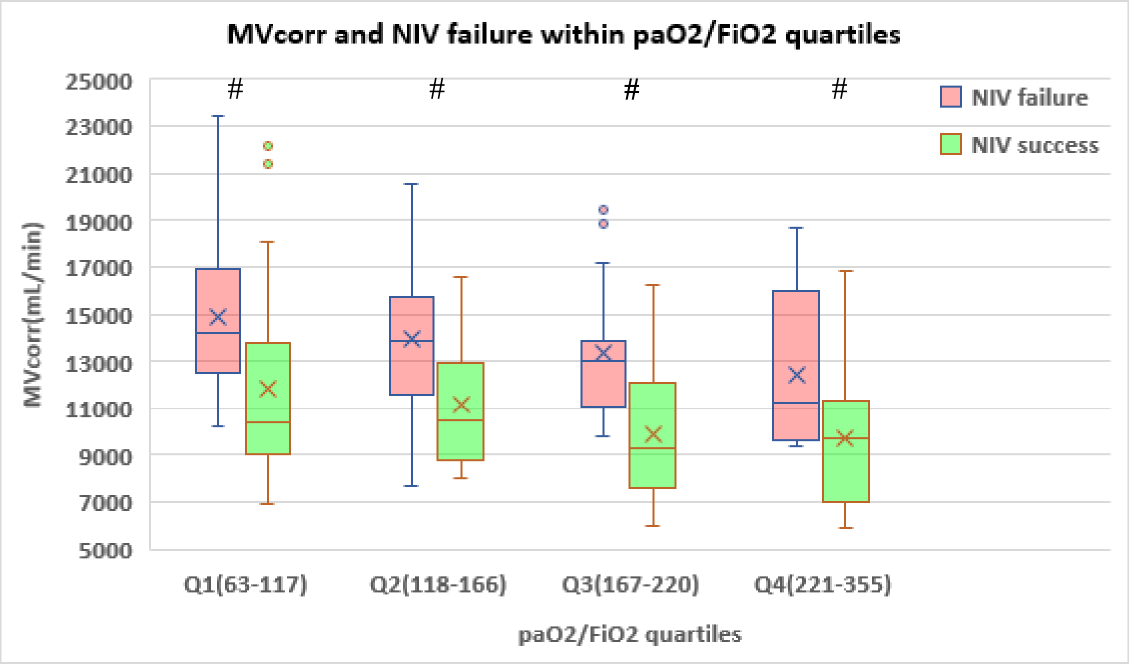

**PEEP (cmH2O)**	**8(7-10)**	**8(7-9)**	**8(7-9)**	**8(7-9)**

Panel B: MVcorr and death

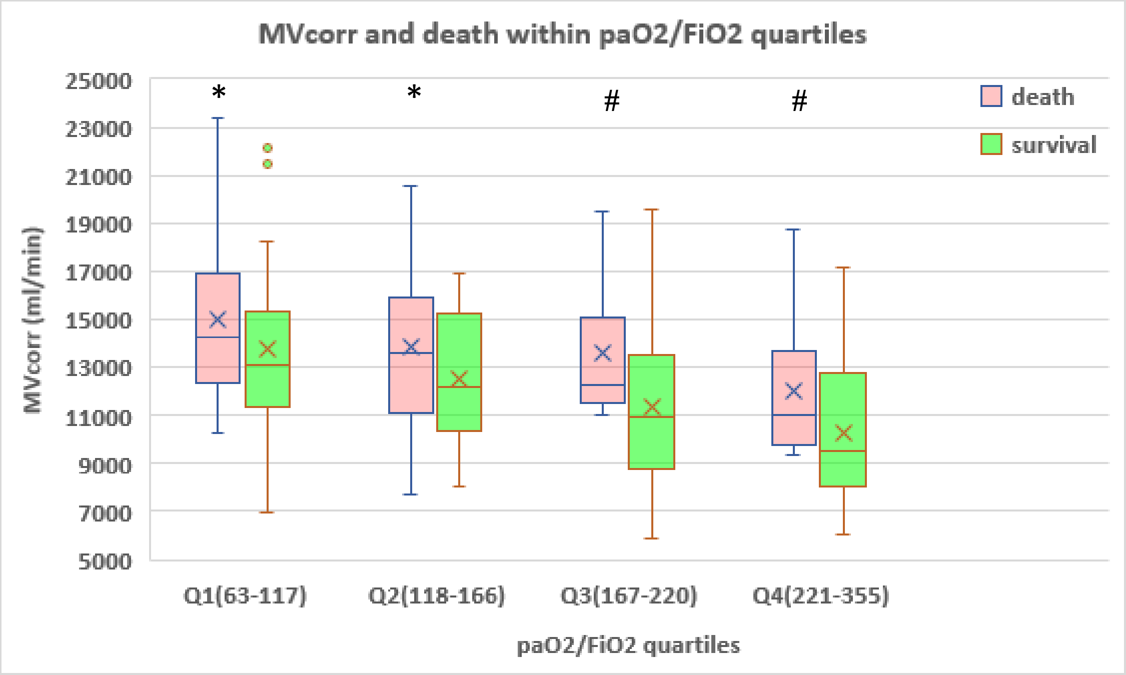

Panel C; MVcorr and ETI

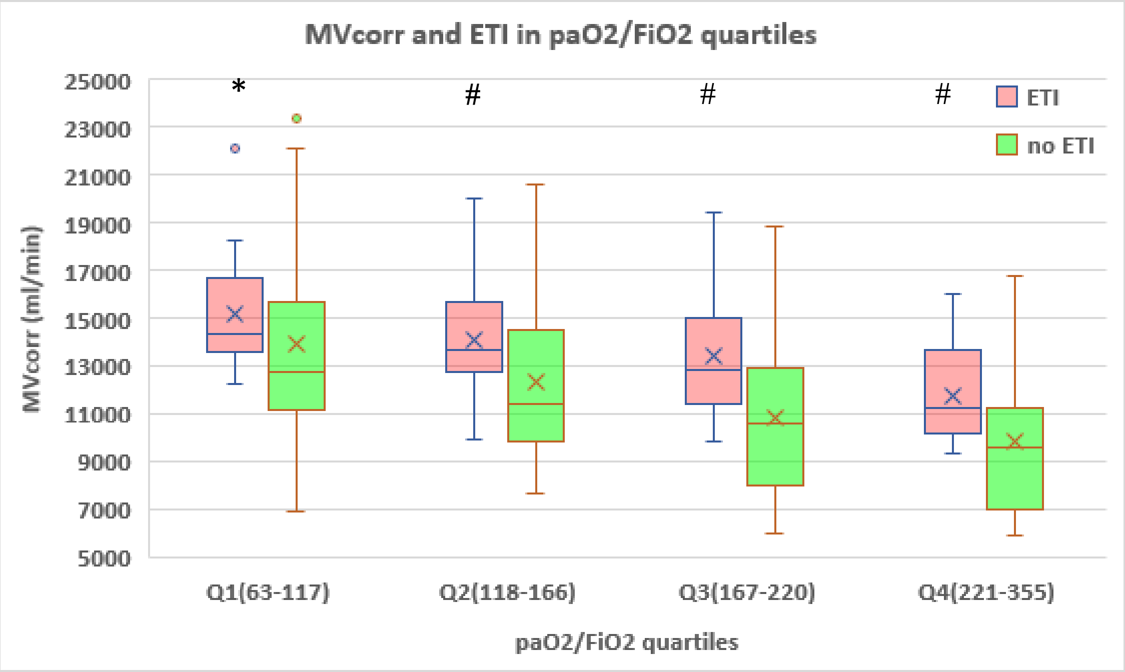

Supplementary Figure 8. Impact of O2-response and CO2-response on NIV failure, death and ETI. CO2-response was assessed via corrected Minute Ventilation(MVcorr).

# P<0.01 vs. other groups ¶ P<0.001 vs. other groups

Panel A

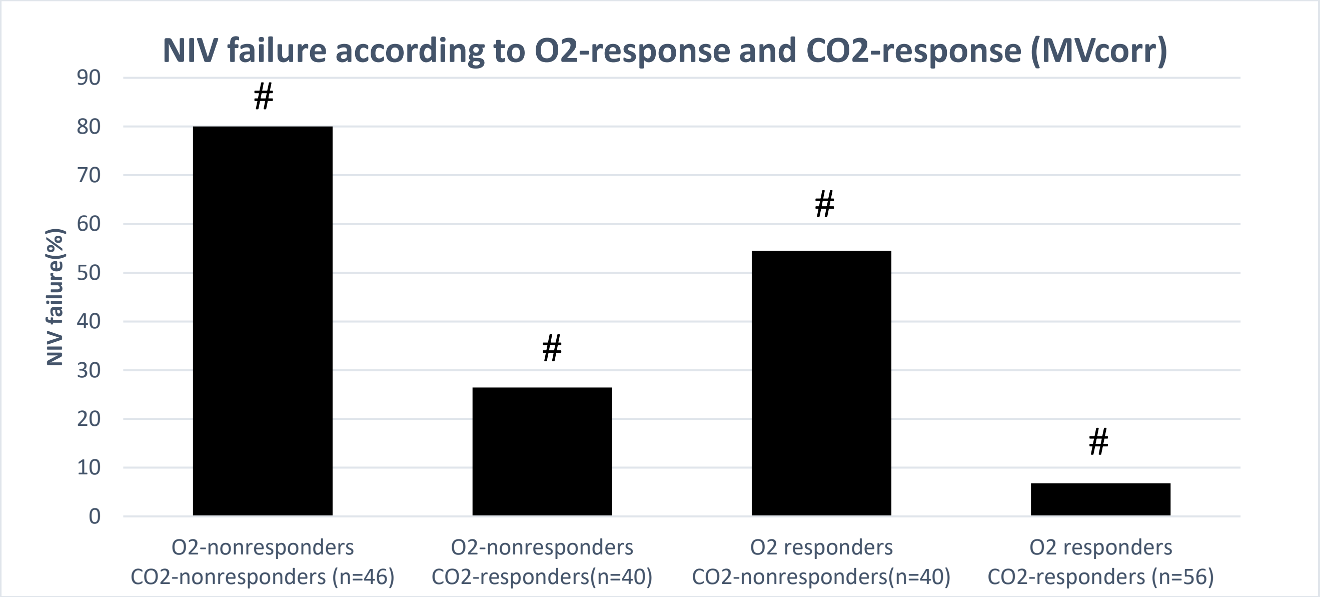

Panel B

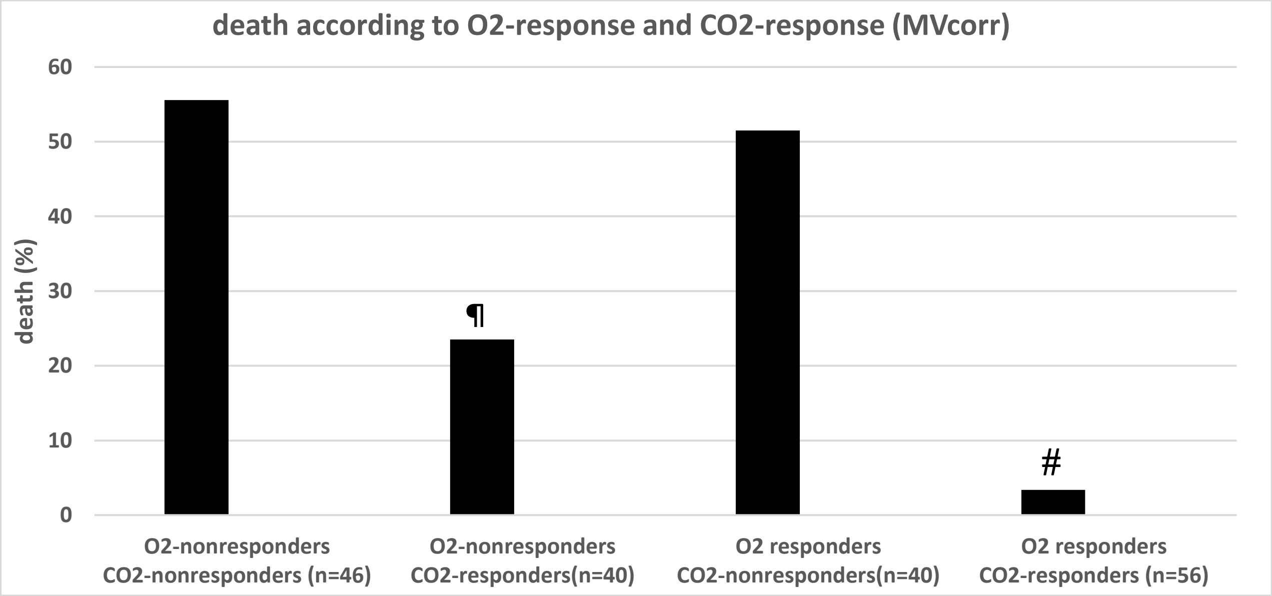

Panel C

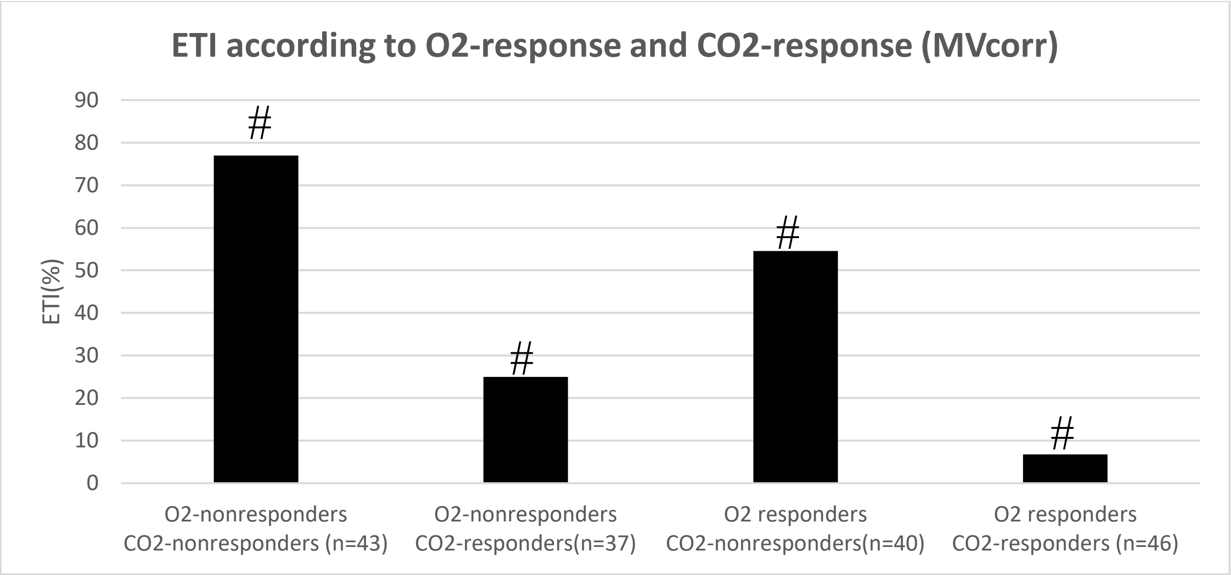
ETI according to O2-response and CO2-response (MVcorr)
90
80
70
60
50
40
30
20
10
0
ETI(%)
#
#
#
#
O2-nonresponders
CO2-nonresponders (n=43)
O2-nonresponders
CO2-responders(n=37)
O2 responders
CO2-nonresponders(n=40)
O2 responders
CO2-responders (n=46)

Supplementary Figure 9: serum procalcitonin in PP group and controls after excluding patients with documented (either at admission or after admission to Subintensive Care Unit) bacterial or mycotic infection (n=223)

# P<0.001 vs. controls

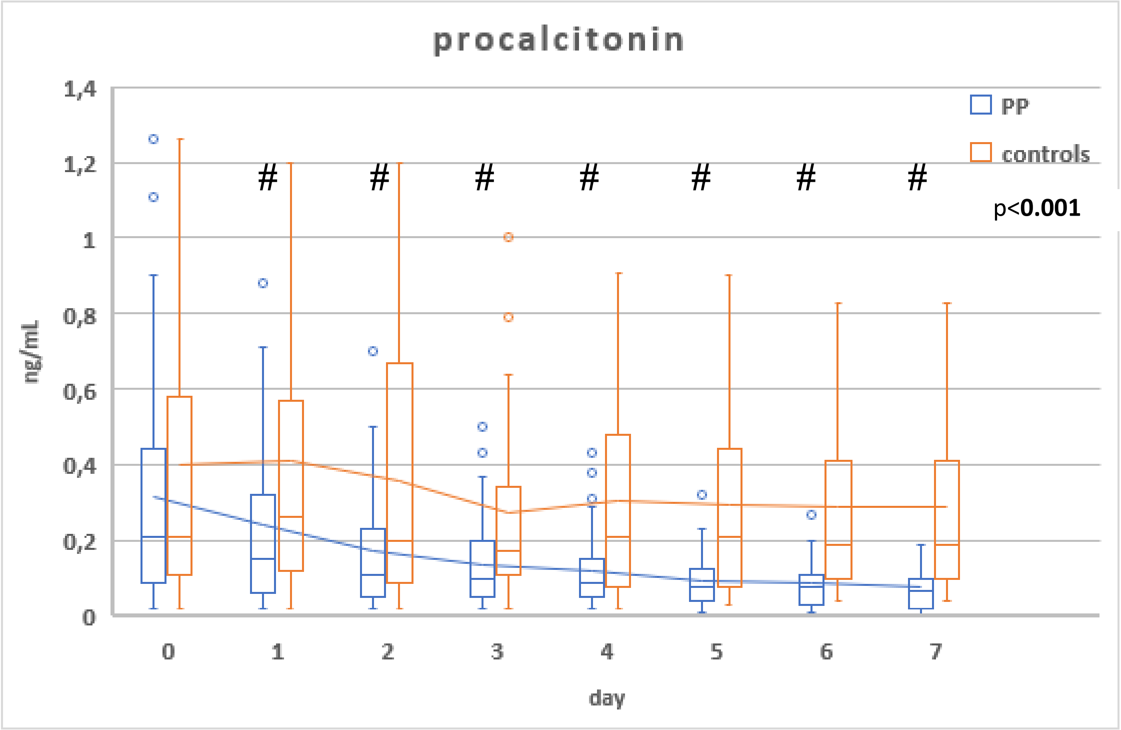

N at risk								
PP	71	71	71	70	70	68	68	68
controls	150	150	150	145	135	122	115	110
