# Supplementary material for: Early prolonged prone position in noninvasively ventilated patients with SARS-CoV-2-related moderate-to-severe hypoxemic respiratory failure: clinical outcomes and mechanisms for treatment response in the PRO-NIV study
Source: Crit Care. 2022 Apr 29;26:118. doi: 10.1186/s13054-022-03937-x (PMC9052189; doi:10.1186/s13054-022-03937-x)
Supplement: Supplementary file 2 — Additional file 2: Supplementary Tables. [file 13054_2022_3937_MOESM2_ESM.docx]

**Supplementary Tables**

**Early prolonged prone position in noninvasively ventilated patients with SARS-CoV-2-related moderate-to-severe hypoxemic respiratory failure: clinical outcomes and mechanisms for treatment response in the PRO-NIV study**

**Supplementary Table 1.** Initial ventilatory settings for all enrolled patients

|  | **Parameter** | **Initial setting** | **Target** | **adjustment** |
| --- | --- | --- | --- | --- |
| **Face-mask** | PEEP(cmH2O) | ≥5 | SpO2≥92% at a FiO2≤50% | increase by 2-3 up to 10-12 |
|  | Pressure support  (cmH2O) | ≥2 | -no patient discomfort  -no accessory muscle activity  -RR<30(min  -VTe≥0.6-0.8 ml/kg PBW  -No uncompensated respiratory acidosis (i.e. PaCO2>45 mmHg with pH<7.35). | increase by 2-3 |
|  | Inspiratory Flow trigger | 0.5-1 L/min | most sensible value to minimize inspiratory effort without visible auto-triggering |  |
|  | Inspiratory Pressure trigger | -1 cm H2O |  |  |
|  | Ti _max_ | 1.2 sec | Prevent inspiratory hang-up |  |
|  | Expiratory trigger | 10-50% of the peak inspiratory flow |  |  |
|  | FiO2 | ≤50%, if possible | SpO2≥92% |  |
|  | PEEP(cmH2O) | ≥10 cmH2O | SpO2≥92% at a FiO2≤50% | increase by 2-3 |
|  | Pressure support  (cmH2O) | ≥8-10 | peak inspiratory flow of 100 l/min |  |
| **Helmet** | Inspiratory Flow trigger | 1 l/min | 1 l/min to minimize inspiratory effort to minimize inspiratory effort without visible auto-triggering |  |
|  | pressurization time | 50 msec or less | Fastest to enable |  |
|  | Ti _max_ | 1.2 sec | Prevent inspiratory hang-up |  |
|  | Expiratory trigger | 10-50% of the peak inspiratory flow | Full support to respiratory muscles without agravating PEEPi | Set low if full support to respiratory muscles is needed  Set high is PEEPi is expected (i.e. COPD) |

**Abbreviations:** Ti _max_: maximum inspiratory time; SpO2: peripheral oxygen saturation; FiO2: fraction of inspired oxygen; PBW: predicted body weight;

**Supplementary Table 2.** Standardized Mean Differences (SMDs) in baseline features between PP and controls before matching and after matching(Panel A). Main clinical efficacy and safety outcomes before and after matching are also reported in panel B

|  | **Unmatched** | |  | **Matched** | |  |
| --- | --- | --- | --- | --- | --- | --- |
| **Panel A:**  **Baseline characteristics** | **Controls (n=174)** | **PP (n=82)** | **SMD** | **Controls**  **(n=162)** | **PP**  **(n=81)** | **SMD** |
| **Age(yr)** | 72(60-78) | 69(60-74) | 0.129 | 68(59-78) | 69(60-74) | 0.046 |
| **Male sex(n, %)** | 72% | 70% | 0.061 | 70% | 71% | 0.097 |
| **Race**  **White, non-hispanic**  **White, hispanic**  **Black** | 90  6  4 | 90  6  4 | 0.047  0.081  0.042 | 88  8  4 | 90  6  4 | 0.091  0.012  0.018 |
| **Time from symptom onset to hospital admission(d)** | 7(4-10) | 7(5-9) | 0.101 | 7(5-9) | 7(5-9) | 0.067 |
| **Time from hospital admission to NIV(hrs)** | 20(6-52) | 20(6-72) | 0.108 | 19(7-48) | 20(6-72) | 0.024 |
| **BMI(kg/m^2^)** | 27.9(25.7-31.2) | 28.3(25.5-33.2) | 0.197 | 28.3(25.6-30.9) | 28.3(26.2-30.1) | 0.072 |
| **Obesity(BMI≥30 kg/m^2^) n(%)** | 28% | 30% | 0.131 | 30% | 30% | 0.081 |
| **Type 2 diabetes mellitus n(%)** | 17% | 21% | 0.021 | 22% | 21% | 0.098 |
| **Hypertension n(%)** | 51% | 56% | 0.071 | 63% | 56% | 0.098 |
| **Chronic lung disease**  **COPD**  **Asthma** | 12%  2% | 19%  4% | 0.142  0.058 | 13%  3% | 19%  4% | 0.059  0.097 |
| **Coronary heart disease n(%)** | 6% | 9% | 0.108 | 8% | 9% | 0.071 |
| **Chronic atrial fibrillation n(%)** | 4% | 5% | 0.039 | 3% | 5% | 0.092 |
| **Chronic kidney disease n(%)** | 10% | 10% | 0.011 | 11% | 10% | -0.055 |
| **History of cancer n(%)** | 2% | 4% | 0.012 | 2% | 4% | 0.034 |
| **Immunocompromised state*** | 3% | 4% | 0.009 | 3% | 4% | 0.013 |
| **Smoking status:**  **former(%)**  **current(%)** | 18%  10% | 19%  7% | 0.021  0.045 | 15%  9% | 19%  7% | 0.084  0.071 |
| **ISARIC 4 C mortality score** | 13(10-15) | 14(11-15) | 0.108 | 14(11-15) | 14(11-15) | 0.067 |
| **SAPS II score** | 35(31-39) | 35(33-39) | 0.103 | 35(33-39) | 35(33-39) | -0.061 |
| **Temperature(°C)** | 36.4(36-36.9) | 36.4(36.1-36.9) | 0.031 | 36.6(36-36.9) | 36.6(36.1-36.9) | 0.037 |
| **Sys BP (mmHg)** | 130(120-140) | 130(120-140) | 0.022 | 130(120-140) | 130(120-140) | 0.097 |
| **Dia BP (mmHg)** | 74(65-80) | 74(65-80) | 0.079 | 74(65-80) | 74(65-80) | 0.061 |
| **Heart Rate (beats/min)** | 85(71-96) | 85(71-96) | 0.081 | 85(71-96) | 85(71-96) | 0.032 |
| **Respiratory rate(breaths/min)** | 28(22-30) | 28(24-30) | 0.086 | 28(22-30) | 28(24-30) | 0.071 |
| **PaO2/FiO2 ratio** | 106(82-139) | 103(80-134) | 0.101 | 103(79-133) | 103(78-130) | 0.068 |
| **PaO2/FiO2 ratio category % patients**  **150-199**  **100-149**  **<100** | 18%  30%  52% | 17%  33%  49% | -0.112  0.092  0.106 | 17%  33%  49% | 17%  33%  49% | 0.012  0.090  0.056 |
| **paCO2 (mmHg)** | 34(31-37) | 32(29-36) | -0.071 | 33(30-37) | 32(29-36) | 0.097 |
| **paCO2>45 mmHg at admission n(%)** | 2 | 2 | 0.042 | 2 | 2 | 0.058 |
| **Arterial pH** | 7.45(7.42-7.48) | 7.45(7.44-7.48) | 0.038 | 7.45(7.44-7.48) | 7.46(7.44-7.48) | 0.031 |
| **Adiuvant therapies** |  |  |  |  |  |  |
| **Dexamethasone** | 96% | 100% | 0.022 | 96% | 100% | 0.012 |
| **Antivirals**  **Remdesivir**  **Lopinavir/ritonavir** | 1  0 | 1  0 | 0.062 | 1  0 | 1  0 | 0.051 |
| **Hydroxychloroquine** | 0 | 0 | 0.024 | 0 | 0 | 0.038 |
| **Tocilizumab** | 0 | 0 | 0.021 | 0 | 0 | 0.012 |
| **Convalescent plasma** | 1 | 0 | 0.014 | 1 | 0 | 0.031 |
| **Enoxaparin** | 96% | 96% | 0.028 | 96% | 96% | 0.021 |
| **Warfarin/DOACs** | 4% | 4% | 0.059 | 4% | 4% | 0.041 |
| **Any SARS-CoV-2 vaccine** | 0(0%) | 0(0%) | 0.038 | 0(0%) | 0(0%) | 0.061 |
| **Ventilatory mode at baseline**  **CPAP**  **PSV** | 54%  46% | 52%  48% | 0.119  0.101 | 52%  48% | 52%  48% | 0.039  0.021 |
| **Interface at baseline**  **Face mask**  **Helmet** | 95%  5% | 93%  7% | 0.102  0.091 | 91%  9% | 93%  7% | 0.096  0.081 |
| **PEEP(cmH2O)**  **Face mask**  **Helmet** | 7(6-9)  10(10-10) | 7(6-8)  10(10-10) | 0.108  0.061 | 7 (6-7)  10(10-10) | 7 (6-7)  10(10-10) | 0.096  0.082 |
| **Pressure Support during PSV (cmH2O)**  **Face mask**  **Helmet** | 4(3-6)  8(7-9) | 4(3-6)  8(7-9) | 0.057  0.081 | 4(3-5)  8(7-9) | 4(3-5)  8(7-9) | 0.094  0.102 |
| **Balance diagnostics across baseline characteristics** | |  |  |  |  |  |
| **Mean SMDs** | | | 0.091 |  | | 0.054 |
| **Proportion of SMDs≥0.10(%)** | | | 30% |  | | 2% |
| **Proportion of SMDs≥0.20(%)** | | | 0% |  | | 0% |
| **Panel B:**  **main clinical efficacy and safety outcomes** | **Controls (n=174)** | **PP (n=82)** | **SMD** | **Controls**  **(n=162)** | **PP**  **(n=81)** | **SMD** |
| **NIV failure** | 46%¶ | 17%¶ | -0.881 | 45%¶ | 17%¶ | -0.873 |
| **Death** | 39%¶ | 12%¶ | -0.929 | 39%¶ | 12%¶ | -0.921 |
| **ETI** | 35%# | 11%# | -0.578 | 34%# | 11%# | -0.522 |
| **back pain** | 11% | 12% | 0.093 | 9% | 12% | 0.102 |
| **intravenous/arterial line dislodgement** | 8% | 6% | 0.118 | 6% | 6% | 0.031 |
| **haemodinamic instability** | 0% | 0% | 0.010 | 0% | 0% | 0.010 |
| **Barotrauma** | 3% | 4% | 0.074 | 3% | 4% | 0.021 |
| **Gastric distension and vomiting** | 0% | 0% | 0.010 | 0% | 0% | 0.010 |
| **device-related: nasal skin ulceration** | 2% | 2% | 0.091 | 2% | 2% | 0.091 |
| **Facial oedema** | 5% | 7% | 0.119 | 4% | 7% | 0.091 |
| **Thoraco-abdominal wall haematoma** | 2% | 3% | 0.127 | 2% | 3% | 0.079 |
| **Venous thrombosis**  **upper limb**  **lower limb** | 3%  0 | 5%  0 | 0.028  0.010 | 3%  0 | 5%  0 | 0.028  0.010 |
| **Subintensive Care Unit-acquired Infection** | 13% | 10% | 0.117 | 10% | 10% | 0.063 |
| **excessive sedation^§^** | 1% | 1% | 0.036 | 1% | 1% | 0.058 |
| **Acute kidney injury requiring renal replacement therapy** | 1% | 1% | 0.039 | 1% | 1% | 0.039 |
| **Liver failure** | 0% | 0% | 0.010 | 0% | 0% | 0.010 |
| **Need for emergency ETI** | 0% | 0% | 0.010 | 0% | 0% | 0.010 |
| **Time to NIV failure(d)** | 4(3, 7) | 9(5, 11) | 0.498 | 6(4, 9) | 9(5, 11) | **0.416** |
| **Time to death(d)** | 8(6, 12) | 14(10, 16) | 0.713 | 8(6, 11) | 14(10, 16) | **0.559** |
| **Time to ETI(d)** | 5(2,8) | 9(5, 11) | 0.617 | 6(4,8) | 9(5, 11) | **0.420** |

**Abbreviations:** ETI: endotracheal intubation; NIV: noninvasive ventilation; SMD: standardized Mean difference

**Supplementary Table 3.** Ventilatory parameters in included patients assessed at 7 days (n=243).

| **Parameter** | **Controls**  **(n=162)** | **Prone Positioning**  **(n=81)** | **P (between-groups)** |
| --- | --- | --- | --- |
| **Ventilatory mode at baseline**  **CPAP**  **PSV** | 84(52%)  78(48%) | 43(53%)  38(47%) | 0.964 |
| **Change in ventilatory mode**  **From CPAP to PSV**  **From PSV to CPAP** | 20(12%)  13(8%)  7(5%) | 11(13%)  4(5%)  7(8%) | 0.946  0.534  0.284 |
| **Timing of ventilator mode change**  **(days since enrollment)** | 6(5-8) | 5(5-7) | 0.713 |
| **Interface at baseline**  **Face Mask**  **Helmet** | 112(66%)  50(34%) | 70(80%)  11(21%) | 0.190 |
| **Change in interface**  **From mask to helmet**  **From helmet to mask** | 18(11%)  13  5 | 10(12%)  8  3 | 0.723  0.938  0.920 |
| **Timing of interface change**  **(days since enrollment)** | 5(4-7) | 6(5-7) | 0.345 |
| **Respiratory support** |  | | |
| **PEEP(cmH2O) ***  **Baseline**  **Face mask**  **Helmet**  **During the study**  **Face mask**  **Helmet**  **Change**  **Face mask**  **Helmet** | 7(6-8)  10(10-10)  9(7-10)  10(10-11)  2(1-3)  0(0-2) | 7 (6-7)  10(10-10)  7(6-7)  10(10-10)  0(-0.5, 0)  0(0-0) | 0.232  0.369  **0.005**  **0.002**  **0.002**  **0.004** |
| **Pressure Support (PS) cmH2O during PSV***  **Baseline**  **Face mask**  **Helmet**  **During the study**  **Face mask**  **Helmet**  **Change**  **Face mask**  **Helmet** | 4(3-6)  8(8,8)  5(4-6)  8(8, 8)  0/0,1)  0(0, 0) | 4(2-5)  8(8-8)  4(2-5)  8(8, 8)  0(0, 0)  0(0, 0) | 0.312  0.683  0.712  0.531  0.219  0.492 |
| **FiO2**  **Baseline**  **During the study**  **Change** | 0.50(0.50-0.70)  0.60(0.50, 0.75)  0.10(0.05, 0.15) | 0.50(0.45-0.70)  0.45(0.40, 0.55)  -0.05(-0.1, 0,00) | 0.323  **<0.0001**  **0.007** |
| **Dyspnoea (CPOT)**  **Baseline**  **During the study**  **Change** | 3(2,4)  2(1, 3)  0(-1, 1) | 3(2,4)  0(-4, 1)  -3(-4, 0) | 0.713  **0.001**  **0.0007** |
| **Device-related discomfort(NRS)**  **Baseline**  **During the study**  **Change** | 2(1, 2)  2(1,2)  0(0, 1) | 2(1, 2)  2(1,2)  0(0,1) | 0.461  0.716  0.812 |
| **RR(breaths/min) ***  **Baseline**  **During the study**  **Change** | 25(20, 30)  24(22-27)  0(-3, 1) | 26(22, 30)  22(20-24)  -4(-5, -1) | 0.328  **0.002**  **0.003** |
| **pH**  **Baseline**  **During the study**  **Change** | 7.45(7.43-7.47)  7.46(7.41, 7.47)  0.001(-0.005, 0.02) | 7.45(7.43-7.47)  7.46(7.44, 7.47)  0.01(-0.005, 0.03) | 0.865  0.139  0.216 |
| **paO2 mmHg**  **Baseline**  **During the study**  **Change** | 83(70-98)  83(72-94)  0(-10, 11) | 82(69-94)  84(77-93)  2(-5, 12) | 0,358  **0.0002**  **0.0002** |
| **paCO2 nnHg**  **Baseline**  **During the study**  **Change** | 37(33-39)  38(34-41)  2(0-4) | 37(32-39)  34(33-36)  -3(-4, -1) | 0.858  **0.0001**  **<0.0001** |
| **PaO2/FiO2 ratio***  **Baseline**  **During the study**  **Change** | 148(115, 200)  155(121, 175)  6(-12, 35) | 155(126, 200)  195(154, 228)  40(15, 67) | 0.336  **<0.0001**  **<0.0001** |
| **VTe (ml/kg prBW)** ^≠^  **Baseline**  **During the study**  **Change** | 7.3 (6.9, 8.1)  7.5 (7.1, 8.3)  0.3(0.0, 0.6) | 7.2(6.9, 8.2)  7.9 (7.1, 8.6)  0.7(0.2, 0.6) | 0.508  0.283  0.212 |
| **MV (L/min) ≠**  **Baseline**  **During the study**  **Change** | 12.9(11.3-15.1)  13.5(11.9-16.1)  0.6(0.3, 1.1) | 13.5(11.7-15.7)  13.9(12.1-15.9)  0.4(0.1, 1.2) | 0.815  0.389  0.478 |
| **VR^≠^**  **Baseline**  **During the study**  **Change** | 1.8(1.6-2.1)  2.1(1.8-2.5)  0.30(-0.05, 0.40) | 1.8(1.4-2.1)  1.3(0.9-1.7)  -0.50(-1.1, -0.1) | 0.567  **0.0001**  **<0.0001** |
| **MVcorr (L/min) ^≠^**  **Baseline**  **During the study**  **Change** | 11.4(9.9-12.1)  12.1(10.1-14.1)  0.7(-0.1, 2.5) | 11.9(10.3-12.6)  10.5(8.9-12.9)  -1.41(-3, 0) | 0.752  **<0.0001**  **<0.0001** |

*Median AUC of respiratory support from baseline to day 7. Baseline is ≥1 hour after NIV initiation in the supine position (in the PP group this means before the first PP session). All measures are made in the supine position: in the PP group, they are made daily in NIV ≥1 hour after terminating the overnight (lasting ≥ 8 hours) PP session.

^≠^for those patients ventilated with full-face mask(n=70 in the PP group and 112 in the controls)

Abbreviations: VR: ventilatory ratio; MV_corr_: corrected minute ventilation; CPOT: Critical-care Pain Observation Tool; NRS: numeric pain rating scale.

**Supplementary Table 4** Baseline and 7-day ventilatory parameters of included patients, grouped according to 28-d NIV failure (n=243).

| **Parameter** | **NIV failure**  **(n=84)** | **NIV success**  **(n=159)** | **P** |
| --- | --- | --- | --- |
| **Age(yr)** | 74(66-79) | 66(56-74) | **<0.001** |
| **Male sex(n, %)** | (73%) | (76%) | 0.512 |
| **Race**  **White, non-hispanic**  **White, hispanic**  **Black** | 155  6  1 | 75  4  2 | 0.401  0.738  0.613 |
| **Time from symptom onset to hospital admission(d)** | 7(5-10) | 7(5-10) | 0,676 |
| **Time from hospital admission to enrollment(d)** | 2(1-3) | 2(1-3) | 0.966 |
| **BMI(kg/m^2^)** | 27.5(25.1-31.7) | 27.3(25.0-31.2) | 0.505 |
| **Obesity(BMI≥30 kg/m^2^) n(%)** | 48(30%) | 23(29%) | 0.612 |
| **Type 2 diabetes mellitus n(%)** | 32(20%) | 17(21%) | 0.667 |
| **Hypertension n(%)** | 94(58%) | 45(56%) | 0.419 |
| **Chronic lung disease**  **COPD**  **Asthma** | 26(16%)  5(3%) | 16(19%)  3(4%) | 0.679  0.513 |
| **Coronary heart disease n(%)** | 13(8%) | 8(9%) | 0.815 |
| **Chronic atrial fibrillation n(%)** | 7(4%) | 4(5%) | 0.771 |
| **Chronic kidney disease n(%)** | 16(10%) | 8(10%) | 0.982 |
| **History of cancer n(%)** | 4(2%) | 3(4%) | 0.512 |
| **Immunocompromised state*** | 5(3%) | 3(4%) | 0.887 |
| **Smoking status:**  **former(%)**  **current(%)** | 23(14%)  14(8%) | 16(19%)  6(7%) | 0.513  0.395 |
| **ISARIC 4 C mortality score** | 14(10-15) | 14(11-15) | 0.536 |
| **SAPS II score** | 38(33-40) | 35(32-39) | **0.007** |
| **Temperature(°C)** | 36.5(36-36.9) | 36.4(36-36.8) | 0.821 |
| **Sys BP (mmHg)** | 130(120-140) | 130(116-140) | 0.767 |
| **Dia BP (mmHg)** | 74(65-80) | 75(68-80) | 0.839 |
| **Heart Rate (beats/min)** | 85(71-96) | 81(70-99) | 0.744 |
| **PP therapy** |  |  |  |
| **Respiratory rate(breaths/min)** | 30(26-32) | 26(20-30) | 0.005 |
| **PaO2/FiO2 ratio** | 90(73-112) | 113(83-142) | **0.006** |
| **paCO2 (mmHg)** | 34(31-37) | 35(31-39) | 0.484 |
| **paCO2>45 mmHg at admission n(%)** | 3 | 2 | 0.739 |
| **Arterial pH** | 7.47(7.43-7.49) | 7.46(7.44-7.49) | 0.680 |
| **Adiuvant therapies for COVID-19** |  |  |  |
| **Steroids** | 162 | 81 | 0.999 |
| **Remdesivir** | 1 | 1 | 0.818 |
| **Tocilizumab** | 4 | 0 | 0.897 |
| **Convalescent plasma** | 0 | 1 | 0.981 |
| **Low molecular weight heparin** | 156(96%) | 78(96%) | 0.912 |
| **Warfarin/DOACs** | 7(4%) | 4(5%) | 0.786 |
| **Dyspnoea (CPOT)**  **Baseline**  **During the study**  **Change** | 3(2,4)  4(3, 5)  1(0, 1) | 3(2,4)  1(0, 1)  -2(-3, -1) | 0.858  0.003  0.009 |
| **RR(breaths/min) ***  **Baseline**  **During the study**  **Change** | 25(20, 30)  25(22, 29)  0(-3, 2) | 26(22, 30)  21(20, 23)  -5(-8, -2) | 0.328  **<0.001**  **<0.001** |
| **paCO2 nnHg**  **Baseline**  **During the study**  **Change** | 36(32, 40)  37(35, 41)  1(0, 2) | 37(33, 39)  37(35, 40)  0(-1, 1) | 0.494  0.109  0.111 |
| **PaO2/FiO2 ratio***  **Baseline**  **During the study**  **Change** | 150(125, 211)  152(113, 196)  2(-10, 27) | 155(126, 200)  222(177, 262)  67(35, 97) | 0.478  **<0.001**  **<0.001** |
| **VTe (ml/kg prBW) ≠**  **Baseline**  **During the study**  **Change** | 7.3 (6.9, 8.1)  7.7 (7.2, 8.5)  0.4(0.2, 0.6) | 7.3(6.5, 8.4)  7.9 (7.1, 8,9)  0.6(0.3, 0.9) | 0.508  0.283  0.368 |
| **MV (L/min) ≠**  **Baseline**  **During the study**  **Change** | 12.6(10.9, 15.2)  13.3(12.2, 16.1)  0.7(0.2, 0.9) | 12.9(11.1-15.1)  12.8(10.9, 15.4)  -0.1(-2.9, -1.7) | 0.812  0.239  0.196 |
| **VR≠**  **Baseline**  **During the study**  **Change** | 1.8(1.6, 2.1)  2.2(1.9, 2.5)  0.4(0.0, 0.9) | 1.8(1.6, 2.2)  1.5(1.2, 1.8)  -0.4(-0.9, -0.1) | 0.496  **<0.001**  **<0.001** |
| **MVcorr (L/min) ≠**  **Baseline**  **During the study**  **Change** | 11.3(10.2, 13.1)  12.5(11.1, 14.1)  1.3(0.6, 2.3) | 11.9(10.6, 13.9)  10.5(8.9, 12.3)  -1.4(-2.5, -0.4) | 0.639  **<0.001**  **<0.001** |

For each parameter median (IQR) is indicated, unless otherwise specified.

The P values value refer to comparison between groups at baseline, at the end of follow-up and to comparison in changes during the follow-up, respectively (statistically significant p-values are written in bold chatacters). Data are expressed as mean±SEM.

**Abbreviations:** DOAC: direct oral anticoagulants; ISARIC: International Severe Acute Respiratory Infection Consortium; NIV: noninvasive ventilation; SAPSS: Simplified acute physiology score

*HIV, ongoing chemiotherapy, chronic immunosuppressor therapy

**Supplementary Table 5** Baseline and 7-day ventilatory parameters of included patients, grouped according to 28-d death (n=243).

| **Parameter** | **Death**  **(n=69)** | **Survive**  **(n=174)** | **P** |
| --- | --- | --- | --- |
| **Age(yr)** | 77(68-80) | 66(56-72) | **<0.001** |
| **Male sex(n, %)** | 116(72%) | 62(76%) | 0.401 |
| **Race**  **White, non-hispanic**  **White, hispanic**  **Black** | 155  6  1 | 75  4  2 | 0.401  0.738  0.613 |
| **Time from symptom onset to hospital admission(d)** | 7(5-10) | 7(5-10) | 0,676 |
| **Time from hospital admission to enrollment(d)** | 2(1-3) | 2(1-3) | 0.966 |
| **BMI(kg/m^2^)** | 27.5(25.1-31.7) | 27.3(25.0-31.2) | 0.505 |
| **Obesity(BMI≥30 kg/m^2^) n(%)** | 48(30%) | 23(29%) | 0.612 |
| **Type 2 diabetes mellitus n(%)** | 31% | 16% | **0.010** |
| **Hypertension n(%)** | 94(58%) | 45(56%) | 0.419 |
| **Chronic lung disease**  **COPD**  **Asthma** | 26(16%)  5(3%) | 16(19%)  3(4%) | 0.679  0.513 |
| **Coronary heart disease n(%)** | 13(8%) | 8(9%) | 0.815 |
| **Chronic atrial fibrillation n(%)** | 7(4%) | 4(5%) | 0.771 |
| **Chronic kidney disease n(%)** | 16(10%) | 8(10%) | 0.982 |
| **History of cancer n(%)** | 4(2%) | 3(4%) | 0.512 |
| **Immunocompromised state*** | 5(3%) | 3(4%) | 0.887 |
| **Smoking status:**  **former(%)**  **current(%)** | 23(14%)  14(8%) | 16(19%)  6(7%) | 0.513  0.395 |
| **ISARIC 4 C mortality score** | 14(12-16) | 12(10-15) | **<0.001** |
| **SAPS II score** | 36(31-39) | 35(32-40) | 0.727 |
| **Temperature(°C)** | 36.5(36-36.9) | 36.4(36-36.8) | 0.821 |
| **Sys BP (mmHg)** | 130(120-140) | 130(116-140) | 0.767 |
| **Dia BP (mmHg)** | 74(65-80) | 75(68-80) | 0.839 |
| **Heart Rate (beats/min)** | 85(71-96) | 81(70-99) | 0.744 |
| **PP therapy** |  |  |  |
| **Respiratory rate(breaths/min)** | 28(21-30) | 28(23-30) | 0.953 |
| **PaO2/FiO2 ratio** | 92(75-115) | 109(82-142) | **0.041** |
| **paCO2 (mmHg)** | 34(31-37) | 35(31-39) | 0.484 |
| **paCO2>45 mmHg at admission n(%)** | 3 | 2 | 0.739 |
| **Arterial pH** | 7.47(7.43-7.49) | 7.46(7.44-7.49) | 0.680 |
| **Adiuvant therapies for COVID-19** |  |  |  |
| **Steroids** | 162 | 81 | 0.999 |
| **Remdesivir** | 1 | 1 | 0.818 |
| **Tocilizumab** | 4 | 0 | 0.897 |
| **Convalescent plasma** | 0 | 1 | 0.981 |
| **Low molecular weight heparin** | 156(96%) | 78(96%) | 0.912 |
| **Warfarin/DOACs** | 7(4%) | 4(5%) | 0.786 |
| **Dyspnoea (CPOT)**  **Baseline**  **During the study**  **Change** | 3(2,4)  4(3, 6)  1(0, 2) | 3(2,4)  1(0, 1)  -2(-3, 0) | 0.858  0.002  0.008 |
| **RR(breaths/min) ***  **Baseline**  **During the study**  **Change** | 25(21, 28)  18(17, 20)  -7(-4, -2) | 26(21, 30)  17(15, 19)  -11(-6, -11) | 0.483  **0.04**  **0.003** |
| **paCO2 nnHg**  **Baseline**  **During the study**  **Change** | 35(31, 39)  37(34, 41)  2(1, 3) | 37(33, 39)  36(35, 38)  -1(-2, 0) | 0.419  0.622  **0.011** |
| **PaO2/FiO2 ratio***  **Baseline**  **During the study**  **Change** | 148(115, 200)  156(102, 221)  8(-13, 21) | 155(126, 200)  211(169, 257)  56(45, 57) | 0.336  **0.002**  **0.001** |
| **VTe (ml/kg prBW) ≠**  **Baseline**  **During the study**  **Change** | 7.4 (7.0, 8.0)  7.6 (7.1, 8.2)  0.2(0.0, 0.4) | 7.3(6.9, 8.0)  7.9 (7.2, 8.6)  0.6(0.3, 0.9) | 0.315  0.283  0.198 |
| **MV (L/min) ≠**  **Baseline**  **During the study**  **Change** | 13.5(12.7,15.5)  15.0(13.4,16.2)  1.5(0.8, 1.0) | 12.9(12.4-13.6)  12.2(8.9-13.9)  -0.7(-0.9, 0.3) | 0.812  0.239  0.144 |
| **VR≠**  **Baseline**  **During the study**  **Change** | 1.8(1.6-2.4)  2.3(1.9, 2.6)  0.5(0.3, 0.8) | 1.9(1.5-2.3)  1.5(1.1, 1.8)  -0.50(-1.1, -0.1) | 0.714  **<0.001**  **<0.001** |
| **MVcorr (L/min) ≠**  **Baseline**  **During the study**  **Change** | 11.8(11.1-14.4)  12.9(12.1-15.1)  1.1(0.8, 1.6) | 12.1(10.8-15.3)  11.0(8.8, 13.9)  -1.0(-3.0, -0.1) | 0.328  **<0.001**  **<0.001** |

For each parameter median (IQR) is indicated, unless otherwise specified.

The P values value refer to comparison between groups at baseline, at the end of follow-up and to comparison in changes during the follow-up, respectively (statistically significant p-values are written in bold chatacters). Data are expressed as mean±SEM.

**Abbreviations:** DOAC: direct oral anticoagulants; ISARIC: International Severe Acute Respiratory Infection Consortium; NIV: noninvasive ventilation; SAPSS: Simplified acute physiology score

*HIV, ongoing chemiotherapy, chronic immunosuppressor therapy

† **#**

**Supplementary Table 6** Baseline and 7-day ventilatory parameters of patients with a full treatment indication, grouped according to endotracheal intubation (ETI) at 28 days(n=208).

| **Parameter** | **ETI**  **(n=50)** | **No ETI**  **(n=158)** | **P** |
| --- | --- | --- | --- |
| **Age(yr)** | 69(69-78) | 68(60-75) | 0.498 |
| **Male sex(n, %)** | 116(72%) | 62(76%) | 0.401- |
| **Race**  **White, non-hispanic**  **White, hispanic**  **Black** | 155  6  1 | 75  4  2 | 0.401  0.738  0.613 |
| **Time from symptom onset to hospital admission(d)** | 7(5-10) | 7(5-10) | 0,676 |
| **Time from hospital admission to enrollment(d)** | 2(1-3) | 2(1-3) | 0.966 |
| **BMI(kg/m^2^)** | 27.5(25.1-31.7) | 27.3(25.0-31.2) | 0.505 |
| **Obesity(BMI≥30 kg/m^2^) n(%)** | 48(30%) | 23(29%) | 0.612 |
| **Type 2 diabetes mellitus n(%)** | 32(20%) | 17(21%) | 0.667 |
| **Hypertension n(%)** | 94(58%) | 45(56%) | 0.419 |
| **Chronic lung disease**  **COPD**  **Asthma** | 26(16%)  5(3%) | 16(19%)  3(4%) | 0.679  0.513 |
| **Coronary heart disease n(%)** | 13(8%) | 8(9%) | 0.815 |
| **Chronic atrial fibrillation n(%)** | 7(4%) | 4(5%) | 0.771 |
| **Chronic kidney disease n(%)** | 16(10%) | 8(10%) | 0.982 |
| **History of cancer n(%)** | 4(2%) | 3(4%) | 0.512 |
| **Immunocompromised state*** | 5(3%) | 3(4%) | 0.887 |
| **Smoking status:**  **former(%)**  **current(%)** | 23(14%)  14(8%) | 16(19%)  6(7%) | 0.513  0.395 |
| **ISARIC 4 C mortality score** | 14(10-15) | 14(11-15) | 0.536 |
| **SAPS II score** | 36(31-39) | 35(32-40) | 0.727 |
| **Temperature(°C)** | 36.5(36-36.9) | 36.4(36-36.8) | 0.821 |
| **Sys BP (mmHg)** | 130(120-140) | 130(116-140) | 0.767 |
| **Dia BP (mmHg)** | 74(65-80) | 75(68-80) | 0.839 |
| **Heart Rate (beats/min)** | 85(71-96) | 81(70-99) | 0.744 |
| **PP therapy** |  |  |  |
| **Respiratory rate(breaths/min)** | 30(26-34) | 26(22-30) | **0.002** |
| **PaO2/FiO2 ratio** | 90(75-108) | 109(80-142) | **0.013** |
| **paCO2 (mmHg)** | 34(31-37) | 35(31-39) | 0.484 |
| **paCO2>45 mmHg at admission n(%)** | 3 | 2 | 0.739 |
| **Arterial pH** | 7.47(7.43-7.49) | 7.46(7.44-7.49) | 0.680 |
| **Adiuvant therapies for COVID-19** |  |  |  |
| **Steroids** | 162 | 81 | 0.999 |
| **Remdesivir** | 1 | 1 | 0.818 |
| **Tocilizumab** | 4 | 0 | 0.897 |
| **Convalescent plasma** | 0 | 1 | 0.981 |
| **Low molecular weight heparin** | 156(96%) | 78(96%) | 0.912 |
| **Warfarin/DOACs** | 7(4%) | 4(5%) | 0.786 |
| **Dyspnoea (CPOT)**  **Baseline**  **During the study**  **Change** | 3(2,4)  4(3, 5)  1(0, 1) | 3(2,4)  1(0, 1)  -2(-3, -1) | 0.858  0.003  0.009 |
| **RR(breaths/min) ***  **Baseline**  **During the study**  **Change** | 25(20, 30)  26(22, 29)  0(-3, 2) | 26(22, 30)  21(20, 24)  -5(-8, -2) | 0.471  0.0002  0.0004 |
| **paCO2 nnHg**  **Baseline**  **During the study**  **Change** | 36(32, 40)  37(35, 41)  1(0, 2) | 37(33, 39)  37(35, 40)  0(-1, 1) | 0.413  0.108  0.124 |
| **PaO2/FiO2 ratio***  **Baseline**  **During the study**  **Change** | 150(125, 211)  152(113, 196)  2(-10, 27) | 155(126, 200)  222(177, 262)  67(35, 97) | 0.512  <0.0001  <0.0001 |
| **VTe (ml/kg prBW) ≠**  **Baseline**  **During the study**  **Change** | 7.3 (6.9, 8.1)  7.7 (7.2, 8.5)  0.4(0.2, 0.6) | 7.3(6.5, 8.4)  7.9 (7.1, 8,9)  0.6(0.3, 0.9) | 0.673  0.312  0.419 |
| **MV (L/min) ≠**  **Baseline**  **During the study**  **Change** | 12.7(10.8, 15.4)  13.1(12.3, 15.9)  0.5(0.2, 0.8) | 12.9(11.1, 15.1)  12.8(10.9, 15.4)  -0.1(-2.9, 1.7) | 0.913  0.281  0.249 |
| **VR≠**  **Baseline**  **During the study**  **Change** | 1.8(1.6, 2.1)  2.2(1.9, 2.5)  0.3(0.0, 0.9) | 1.8(1.6, 2.2)  1.4(1.1, 1.7)  -0.4(-0.9, -0.1) | 0.813  **<0.001**  **<0.001** |
| **MVcorr (L/min) ≠**  **Baseline**  **During the study**  **Change** | 11.4(10.3, 13.2)  12.5(11.1, 14.1)  1.3(0.6, 2.3) | 11.9(10.5, 13.8)  10.5(8.9, 12.3)  -1.4(-2.5, -0.4) | 0.641  **<0.001**  **<0.001** |

For each parameter median (IQR) is indicated, unless otherwise specified.

The P values value refer to comparison between groups at baseline, at the end of follow-up and to comparison in changes during the follow-up, respectively (statistically significant p-values are written in bold chatacters). Data are expressed as mean±SEM.

**Abbreviations:** DOAC: direct oral anticoagulants; ISARIC: International Severe Acute Respiratory Infection Consortium; NIV: noninvasive ventilation; SAPSS: Simplified acute physiology score

*HIV, ongoing chemiotherapy, chronic immunosuppressor therapy

**Supplementary Table 7.** Cox multivariate analysis of baseline predictors of NIV failure, death and endotracheal intubation (ETI) at 28 days in the whole study population (n=243).

| **NIV failure** | | | |
| --- | --- | --- | --- |
| **Parameter** | **HR** | **95%CI** | **P** |
| **Age** | 1.0465 | 1.0024 to 1.0926 | **0.039** |
| **SAPS II** | 1.0001 | 0.9199 to 1.0874 | 0.999 |
| **PP therapy** | 0.1948 | 0.1091 to 0.3481 | **<0.001** |
| **paO2/FiO2 at admission** | 0.9942 | 0.9873 to 1.0013 | 0.110 |
| **RR at admission** | 1.0736 | 1.0287 to 1.1203 | **0.001** |
| **Death** | | | |
| **Parameter** | **HR** | **95%CI** | **P** |
| **Age** | 1.0891 | 1.0327 to 1.1486 | **0.002** |
| **Type 2 DM** | 1.8941 | 1.0192 to 3.5202 | **0.044** |
| **ISARIC 4C score** | 1.0259 | 0.8612 to 1.2221 | 0.392 |
| **PP therapy** | 0.1413 | 0.0698 to 0.2859 | **<0.001** |
| **paO2/FiO2 at admission** | 0.9953 | 0.9870 to 1.0036 | 0.269 |
| **Endotracheal intubation*** | | | |
| **Parameter** | **HR** | **95%CI** | **P** |
| **PP therapy** | 0.1850 | 0.0867 to 0.3948 | **<0.001** |
| **pO2/FiO2 at admission** | 0.9946 | 0.9849 to 1.0044 | 0.284 |
| **RR at admission** | 1.0618 | 1.0160 to 1.1096 | **0.008** |

*for those with a full-treatment indication (n=208)

**Abbreviations:** ISARIC: International Severe Acute Respiratory Infection Consortium; NIV: noninvasive ventilation; RR: respiratory rate; SAPS: Simplified acute physiology score

**Supplementary Table 8**. Change in lung ultrasound (LUS) indices of lung disease severity in patients with baseline and 5-day LUS examination (n=187).

|  | | **PP**  **(n=81)** | | | | **Controls**  **(n=106)** | | | |  |
| --- | --- | --- | --- | --- | --- | --- | --- | --- | --- | --- |
| **Parameter** | | **Baseline** | **Day 5** | **Change** | **P**  **(change from baseline)** | **Baseline** | **Day 5** | **Change** | **P**  **(change from baseline)** | **P**  **(between groups)** |
| **LUS score** | **ventral** | 6  (4,8) | 5  (4, 7) | -1  (-3, 0) | 0.002 | 6  (5,8) | 5  (3, 7) | -1  (-2,-1) | 0.025 | 0.003 |
|  | **lateral** | 8  (7, 10) | 6  (5, 7) | -2  (-4, -1) | <0.001 | 8  (7, 10) | 8  (7, 10) | 0  (-1,0) | 0.883 | <0.001 |
|  | **dorsal** | 10  (8, 10) | 6  (5, 8) | -3  (-4,-2) | <0.001 | 10  (8, 10) | 10  (8, 11) | 0  (0, 0) | 0.378 | <0.001 |
|  | **global** | 24  (20, 27) | 18  (14, 21) | -6  (-10, -4) | <0.001 | 23  (20, 27) | 22  (19, 27) | -1  (-2, 0) | 0.259 | <0.001 |
| **N-consolidated regions** | **Ventral** | 0  (0,1) | 0  (0,0) | 0  (-1, 0) | 0.003 | 0  (0,1) | 0  (0,0) | 0  (0, 0) | 0.914 | <0.001 |
|  | **Lateral** | 1  (0,3) | 0  (0,1) | -1  (-2, 0) | <0.001 | 1(  0,3) | 1  (0,3) | 0  (0, 0) | 0.495 | <0.001 |
|  | **Dorsal** | 2  (1,3) | 0  (0,1) | -2  (-2, -1) | <0.001 | 2  (1,3) | 2  (1,3) | 0  (0, 1) | 0.108 | <0.001 |
|  | **global** | 4  (2,6) | 1  (0,2) | -3  (-4, -2) | <0.001 | 4  (2,6) | 4  (2,6) | 0  (0, 1) | 0.387 | <0.001 |
| **LUS reaeration score** | **ventral** | - | - | 1  (0,3) | - | - | - | 1  (0,1) | - | 0.003 |
|  | **lateral** | - | - | 3  (2,5) | - | - | - | 0  (0,1) | - | <0.001 |
|  | **dorsal** | - | - | 4  (3,6) | - | - | - | 0  (0,0) | - | <0.001 |
|  | **global** | - | - | 9  (6, 11) | - | - | - | 1  (0, 2) | - | <0.001 |

All data are expressed as median(IQR).

Legend:

**LUS score ventral**: the score is the sum of individual scores for region L1, L2, R1, R2

**LUS score lateral**: the score is the sum of individual scores for region L3, L4, R3, R4

**LUS score dorsal**: the score is the sum of individual scores for region L5, L6, R5, R6

**LUS score total**: the score is the sum of ventral, lateral and dorsal scores

**N-consolidated regions**: is the number of regions with LUS score 3

**LUS reaeration score**: is calculated for anterior, lateral and dorsal regions according to the method proposed and validated by Bouhemad et al

**Supplementary Table 9**. Cox multivariate analysis of predictors of 28-d NIV failure, death and endotracheal intubation (ETI), after inclusion of gas exchange responses and PEEP at day 1 in supine position (timepoint sp1). CO2 response was assessed via corrected Minute Ventilation(MVcorr)(n=182).

| **NIV failure** | | | |
| --- | --- | --- | --- |
| **Parameter** | **HR** | **95%CI** | **P** |
| **Age** | 1,0182 | 0,9932 to 1,0439 | 0.157 |
| **SAPS II** | 1,0337 | 0,9269 to 1,1528 | 0.553 |
| **PP therapy** | 0,2494 | 0,1317 to 0,4726 | **<0.001** |
| **paO2/FiO2 at admission** | 0,9926 | 0,9884 to 1.0996 | 0.127 |
| **RR at admission** | 1,0488 | 0.9816 to 1.1822 | 0.283 |
| **O2-response** | 05408 | 0.3638 to 0.9410 | **0.039** |
| **CO2-response(MVcorr)** | 0.3887 | 0.2365 to 0.6388 | **<0.001** |
| **PEEP (day 1)** | 0,9033 | 0,7880 to 1,0355 | 0.147 |
| **Death** | | | |
| **Age** | 1.0588 | 1.0267 to 1.0919 | **0.003** |
| **Type 2 DM** | 1.5400 | 0.8416 to 2.8179 | 0.164 |
| **ISARIC 4C score** | 1,0367 | 0,9079 to 1,1838 | 0.596 |
| **PP therapy** | 0,1942 | 0,0887 to 0,4253 | **<0.001** |
| **paO2/FiO2 at admission** | 0.9904 | 0.9712 to 1.2103 | 0.548 |
| **O2-responder** | 1.0287 | 0.9910 to 1.0679 | 0.191 |
| **CO2-responder(MVcorr)** | 0.3176 | 0.1146 to 0.4816 | **0.001** |
| **PEEP (day 1)** | 1.0586 | 0.9221 to 1.2152 | 0.421 |
| **Endotracheal intubation*** | | | |
| **PP therapy** | 0.2133 | 0.0905 to 0.5030 | **<0.001** |
| **pO2/FiO2 at admission** | 0,9916 | 0,9758 to 1.0974 | 0.117 |
| **RR at admission** | 0.9508 | 0.9145 to 1,1885 | 0.115 |
| **O2-responder** | 0,4420 | 0.2818 to 0.7432 | **0.019** |
| **CO2-responder(MVcorr)** | 0.3420 | 0.1818 to 0.6432 | **<0.001** |
| **PEEP (day 1)** | 0.8538 | 0.7143 to 1.0205 | 0.184 |

*for those with a full-treatment indication (n=166)

**Abbreviations:** ISARIC: International Severe Acute Respiratory Infection Consortium; NIV: noninvasive ventilation; SAPS: Simplified acute physiology score

**Supplementary Table 10.** Multivariable linear regression of predictors of changes in CRP, D-dimer and neutrophil-to-lymphocyte ratio (NLR)(n=182). Changes through days 1-7 were quantified via the trapezoid method and AUC calculation.

| **Changes in serum CRP** | | | | | | |
| --- | --- | --- | --- | --- | --- | --- |
| **Parameter** | **r** | **SE** | **P** | **VIF** | **R^2^** | **R^2^_adj_** |
| **CRP1** | 0.59 | 0.004 | **<0.001** | 1.65 | 0.65 | 0.64 |
| **LUS reaeration score** | -0.35 | 0006 | **0.002** | 1.53 |  |  |
| **CO2-responders(VR1)** | -0.37 | 0.011 | **0.019** | 1.13 |  |  |
| **Not included in the model** |  |  |  |  |  |  |
| **Obesity** | 0.29 | 0.249 | 0.138 | 1.12 |  |  |
| **O2-responder** | 0.21 | 0.318 | 0.213 | 1.32 |  |  |
| **PEEP (day 1-2)** | 0.191 | 0.219 | 0.513 | 1.29 |  |  |
| **Changes in plasma D-dimer** | | | | | | |
| **Parameter** | **r** | **SE** | **P** | **VIF** | **R^2^** | **R^2^_adj_** |
| **D-dimer (day 1)** | 0.60 | 0.012 | **<0.001** | 1.06 | 0.60 | 0.58 |
| **LUS reaeration score** | -0.36 | 0.005 | **0.009** | 1.81 |  |  |
| **CO2-responder (VRsp1)** | -0.34 | 0.011 | **0.020** | 1.32 |  |  |
| **Not included in the model** |  |  |  |  |  |  |
| **O2-responder (pO2/FiO2sp1)** | -0.21 | 0.231 | 0.380 | 0.09 |  |  |
| **PEEP (day 1)** | 0.15 | 0.118 | 0.501 | 1.20 |  |  |
| **Changes in neutrophil-to-lymphocyte ratio (NLR)** | | | | | | |
| **Parameter** | **r** | **SE** | **P** | **VIF** | **R^2^** | **R^2^_adj_** |
| **ISARIC 4C score** | 0.12 | 0.001 | **0.005** | 1.57 | 0.65 | 0.64 |
| **LUS reaeration score** | -0.39 | 0.001 | **<0.001** | 1.06 |  |  |
| **Initial NLR** | -0.65 | 0.001 | **<0.001** | 1,78 |  |  |
| **Not included in the model** |  |  |  |  |  |  |
| **O2-responder (pO2/FiO2sp1)** | -0.03 | 0.182 | 0.316 | 1.41 |  |  |
| **CO2-responder (VRsp1)** | -0.25 | 0.095 | 0.091 | 1.02 |  |  |
| **PEEP (day 1)** | 0.01 | 0.112 | 0.110 | 1.15 |  |  |
| **SAPS II** | 0.07 | 0.213 | 0.380 | 1.46 |  |  |

**Supplementary Table 11.** Per protocol analysis: efficacy and safety outcomes in patients included in the pre-specified per protocol analysis, after excluding those who failed PP therapy(in the PP group) and those who underwent rescue PP therapy (in controls) (n=232).

| **Outcome** | **Controls**  **(n=152)** | **Prone position**  **(n=80)** | **Absolute or mean difference (95% CI)** | **HR**  **(95% CI)** | **P** |
| --- | --- | --- | --- | --- | --- |
| **Primary outcome** | | | | | |
| **NIV failure at 28 d** | 62(41%) | 13(16%) | -25%  (-17%, -42%) | 0.32  (0.20, 0.51)   \|  \| \| --- \| \|  \| | <0.001 |
| **Secondary outcomes** | | | | | |
| **Death at 28 d** | 55(36%) | 9(11%) | -25%  (-15%, -40%) | \|  \| \| --- \| \|  \|   0.25  (0.15, 0.42) | <0.001 |
| **ETI at 28 d*** | 34(27%) | 8(10%) | -22%  (-13%, -39%) | 0.32(0.18-0.59)   \|  \| \| --- \| \|  \| | 0.002 |
| **Length of stay in**  **Subintensive Care Unit(d)** | 7(5,9) | 6(5,8) | -1(-2, 0) |  | **0.045** |
| **Days of invasive mechanical ventilation(d)** | 8(2, 15) | 6(2, 12) | -1(-2, 2) |  | 0.630 |
| **Death in invasively ventilated mechanically ventilated patients** | 25(74%) | 3(35%) | -39%(-18% to -84%) | 0.27(0.12-0.55) | **0.009** |
| **Length of hospital stay(d):**  **-whole study population**  **-hospital survivors** | 16(12,20)  19(15, 22) | 15(10,20)  15(10, 20) | 0(-1, 0)  -3(-5, -1) |  | 0.141  **0.039** |
| **Daily hours of NIV** | 20.3(15.9, 22.2) | 20.1(18.2, 22.4) | -1(0, -1) |  | **0**.812 |
| **Total days of NIV at 28 days** | 7(5,9) | 6(5,8) | -1(-2, 0) |  | **0.045** |
| **Daily hours of PP** | - | 12.2(10.1, 13.8) | - |  |  |
| **Duration of the longest PP session each day** | - | 10.9(9.1, 13.3) | - |  |  |
| **Number of PP sessions each day** | - | 2(1, 3) | - |  |  |
| **Total days of PP therapy at 28 days** | - | 6(5, 8) | - |  |  |
| **Device-related discomfort(NRS) at 28 days**  **Baseline**  **During the study** | 2(1, 2)  2(1,2) | 2(1, 2)  2(1,2) | 0(0, 1)  0(0,1) |  | 0.461  0.716  0.812 |
| **Dyspnoea (CPOT) at 28 days**  **Baseline**  **During the study** | 3(2,4)  1(0, 3) | 3(2,4)  0(-4, 1) | 0(0, 1)  -2(-3, 0) |  | 0.489  0.001 |
| **Safety endpoints** |  |  |  |  |  |
| **back pain** | 14(9%) | 10(12%) |  |  |  |
| **intravenous/arterial line dislodgement** | 8(5%) | 4(5%) |  |  |  |
| **haemodinamic instability** | 0(0%) | 0(0%) |  |  |  |
| **Barotrauma** †  **Pneumothorax/**  **Pneumomediasinum**  **Subcutaneous emphysema** | 4(3%)  1(1%)  4(3%)  4(2%) | 4(4%)  0(0%)  4(5%)  3(4%) |  |  |  |
| **Gastric distension and vomiting** | 0(0%) | 0(0%) |  |  |  |
| **device-related: nasal skin ulceration** | 3(2%) | 2(2%) |  |  |  |
| **Facial oedema** | 5(3%) | 6(7%) |  |  |  |
| **Thoraco-abdominal wall haematoma** | 2(1%) | 3(3%) |  |  |  |
| **Venous thrombosis**   - **upper limb**   **lower limb** | 5(3%)  0 | 4(5%)  0 |  |  |  |
| **Subintensive Care Unit-acquired Infection** | 12(7%) | 8(10%) |  |  |  |
| **excessive sedation^§^** | 1(1%) | 1(1%) |  |  |  |
| **Acute kidney injury requiring renal replacement therapy** | 1(1%) | 1(1%) |  |  |  |
| **Liver failure** | 0(0%) | 0(0%) |  |  |  |
| **Need for emergency ETI** | 0(0%) | 0(0%) |  |  | 0.999 |
| **Time to NIV failure(d)** | 4(2, 8) | 9(5, 13) |  |  | **0.020** |
| **Time to death(d)** | 8(6, 11) | 14(10, 16) |  |  | **0.013** |
| **Time to ETI(d)** | 4(2,8) | 9(4, 10) |  |  | **0.028** |
| **Reason for ETI**† |  |  |  |  |  |
| **Worsening or nonimproving hypoxemia** | 44(32%) | 8(10%) |  |  |  |
| **Respiratory muscle fatigue** | 22(16%) | 6(7%) |  |  |  |
| **Worsening or unbearable dyspnoea** | 40(25%) | 4(5%) |  |  |  |
| Intolerance to treatment | 4(2%) | 1(1%) |  |  |  |
| Altered mental status | 1(1%) | 0(0%) |  |  |  |
| Shock | 0(0%) | 0(0%) |  |  |  |
| Hypercapnia | 1(1%) | 0(0%) |  |  |  |
| Inability to clear secretions | 1(1%) | 0(0%) |  |  |  |
| **Extracorporeal membrane Oxygenation** | 1(1%) | 0(0%) |  |  |  |

**Abbreviations:** ETI: endotracheal intubation; NIV: noninvasive ventilation;

*Among patients with a full treatment indication(n=208)

† Subcategories are not mutually exclusive and may not necessarily sum to the category total

^§^Defined by a RASS<-3 for more than 30 minutes

^¶^ For those patients with a full treatment indication

**Supplementary Table 12.** Baseline and main efficacy and safety outcomes of included patients, grouped according to the initial interface used (n=243).

|  | **Face mask** | | | **Helmet** | | |
| --- | --- | --- | --- | --- | --- | --- |
| **Parameter** | **Controls**  **(n=102)** | **PP**  **(n=70)** | **P** | **Controls**  **(n=48)** | **PP**  **(n=13)** | **P** |
| **Age(yr)** | 72(68-71) | 70(64-77) | 0.814 | 69(69-78) | 68(60-75) | 0.481 |
| **Male sex(n, %)** | 79% | 72% | 0.516 | 72% | 76% | 0.829 |
| **Race**  **White, non-hispanic**  **White, hispanic**  **Black** | 91  6  3 | 95  3  2 | 0.815  0.717  0.629 | 155  6  1 | 75  4  2 | 0.498  0.717  0.626 |
| **Time from symptom onset to hospital admission(d)** | 7(5-10) | 7(5-10) | 0.348 | 7(5-10) | 7(5-10) | 0.538 |
| **Time from hospital admission to enrollment(d)** | 2(1-3) | 2(1-3) | 0.857 | 2(1-3) | 2(1-3) | 0.912 |
| **BMI(kg/m^2^)** | 27.6(25.4-31.5) | 27.5(25.2-31.3) | 0.315 | 27.5(25.1-31.7) | 27.3(25.0-31.2) | 0.513 |
| **Obesity(BMI≥30 kg/m^2^) n(%)** | 32% | 31% | 0.492 | 48(30%) | 23(28%) | 0.637 |
| **Type 2 diabetes mellitus n(%)** | 21%) | 21% | 0.899 | 20% | 20% | 0.713 |
| **Hypertension n(%)** | 58% | 56% | 0.921 | 55% | 52% | 0.815 |
| **Chronic lung disease**  **COPD**  **Asthma** | 15%  2% | 17%  3% | 0.497  0.581 | 15%  3% | 19%  4% | 0.359  0.416 |
| **Coronary heart disease n(%)** | 5% | 7% | 0.592 | 8% | 8% | 0.873 |
| **Chronic atrial fibrillation n(%)** | 3% | 4% | 0.839 | 3% | 4% | 0.693 |
| **Chronic kidney disease n(%)** | 10% | 9% | 0.763 | 10% | 8% | 0.735 |
| **History of cancer n(%)** | 2% | 3% | 0.516 | 2% | 4% | 0.731 |
| **Immunocompromised state*** | 1% | 4% | 0.812 | 3% | 4% | 0.813 |
| **Smoking status:**  **former(%)**  **current(%)** | 13%  8% | 16%  8% | 0.394  0.478 | 11%  8% | 15%  9% | 0.313  0.411 |
| **ISARIC 4 C mortality score** | 14(10-15) | 14(12-16) | 0.591 | 14(10-15) | 14(11-15) | 0.536 |
| **SAPS II score** | 35(31-39) | 35(32-39) | 0.839 | 36(31-39) | 35(32-40) | 0.727 |
| **Temperature(°C)** | 36.5(36-36.9) | 36.4(36-36.8) | 0.348 | 36.5(36-36.9) | 36.4(36-36.8) | 0.821 |
| **Sys BP (mmHg)** | 130(120-140) | 130(116-140) | 0.413 | 130(120-140) | 130(116-140) | 0.767 |
| **Dia BP (mmHg)** | 74(65-80) | 75(68-80) | 0.592 | 74(65-80) | 75(68-80) | 0.839 |
| **Heart Rate (beats/min)** | 85(71-96) | 81(70-99) | 0.637 | 85(71-96) | 81(70-99) | 0.744 |
| **Respiratory rate(breaths/min)** | 28(21-30) | 28(23-30) | 0.916 | 28(21-30) | 28(23-30) | 0.953 |
| **PaO2/FiO2 ratio** | 100(80-137) | 102(80-140) | 0.479 | 102(78-140) | 104(80-142) | 0.539 |
| **PaO2/FiO2 ratio category n(%) patients**  **150-199**  **100-149**  **<100** | 18%  32%  50% | 18%  32%  50% | 0.999  0.891  0.713 | 15%  35%  50% | 19%  31%  50% | 0.713  0.814  0.358 |
| **paCO2 (mmHg)** | 34(31-37) | 35(31-39) | 0.892 | 34(31-37) | 35(31-39) | 0.484 |
| **paCO2>45 mmHg at admission n(%)** | 2 | 2 | 0.892 | 3 | 2 | 0.713 |
| **Arterial pH** | 7.47(7.43-7.49) | 7.46(7.44-7.49) | 0.872 | 7.47(7.43-7.49) | 7.46(7.44-7.49) | 0.739 |
| **Main efficacy outcomes** |  | | | | | |
| **NIV failure** | 45% | 15% | 0.0002 | 48% | 7% | 0.001 |
| **Death** | 38% | 11% | 0.0001 | 36% | 6% | 0.010 |
| **ETI** | 30% | 10% | 0.004 | 36% | 2% | 0.030 |
| **Safety end-points** |  | | | | | |
| **back pain** | 9% | 10% | 0.416 | 7% | 10% | 0.418 |
| **intravenous/arterial line dislodgement** | 6% | 6% | 0.639 | 6% | 6% | 0.397 |
| **haemodinamic instability** | 0% | 0% | 0.791 | 0% | 0% | 0.498 |
| **Barotrauma †**  **Pneumothorax/**  **Pneumomediasinum**  **Subcutaneous emphysema** | 2%  1%  3%  2% | 3%  0%  4%  3% | 0.713  0.515  0.413  0.398 | 3%  1%  3%  2% | 4%  0%  5%  5% | 0.682  0.779  0.319  0.438 |
| **Gastric distension and vomiting** | 0% | 0% | 0.999 | 0% | 0% | 0.999 |
| **device-related: nasal skin ulceration** | 2% | 2% | 0.612 | 0% | 0% | 0.313 |
| **Facial oedema** | 3% | 6% | 0.498 | 2% | 5% | 0.513 |
| **Thoraco-abdominal wall haematoma** | 0%) | 0% | 0.999 | 1% | 3% | 0.291 |
| **Venous thrombosis**   - **upper limb**   **lower limb** | 1%  0 | 3%  0 | 0.698  0.713 | 4%  0 | 6%  0 | 0.713  0.912  0.898 |
| **Subintensive Care Unit-acquired Infection** | 7% | 9% | 0.445 | 6% | 7% | 0.398 |
| **excessive sedation^§^** | 1% | 1% | 0.999 | 1% | 0% | 0.781 |
| **Acute kidney injury requiring renal replacement therapy** | 1% | 1% | 0.999 | 1% | 1% | 0.818 |
| **Liver failure** | 0% | 0% | 0.999 | 0% | 0% | 0.981 |
| **Need for emergency ETI** | 0% | 0% | 0.999 | 0% | 0% | 0.999 |
| **Time to NIV failure(d)** | 5(3, 9) | 9(5, 12) | 0.879 | 5(3, 9) | 9(6, 12) | 0.489 |
| **Time to death(d)** | 8(6, 11) | 14(10, 16) | 0.398 | 8(6, 11) | 14(10, 16) | 0.312 |
| **Time to ETI(d)** | 5(2,8) | 9(4, 10) | 0.297 | 5(2,8) | 9(4, 10) | 0.298 |

**Supplementary Table 13.** Baseline and main efficacy and safety outcomes of included patients, grouped according to the initial ventilatory mode (n=243).

|  | **CPAP** | | | **PSV** | | |
| --- | --- | --- | --- | --- | --- | --- |
| **Parameter** | **Controls**  **(n=84)** | **PP**  **(n=43)** | **P** | **Controls**  **(n=78)** | **PP**  **(n=38)** | **P** |
| **Age(yr)** | 72(68-71) | 70(64-77) | 0.814 | 69(69-78) | 68(60-75) | 0.481 |
| **Male sex(n, %)** | 79% | 72% | 0.516 | 72% | 76% | 0.829 |
| **Race**  **White, non-hispanic**  **White, hispanic**  **Black** | 91  6  3 | 95  3  2 | 0.815  0.717  0.629 | 155  6  1 | 75  4  2 | 0.498  0.717  0.626 |
| **Time from symptom onset to hospital admission(d)** | 7(5-10) | 7(5-10) | 0.348 | 7(5-10) | 7(5-10) | 0.538 |
| **Time from hospital admission to enrollment(d)** | 2(1-3) | 2(1-3) | 0.857 | 2(1-3) | 2(1-3) | 0.912 |
| **BMI(kg/m^2^)** | 27.6(25.4-31.5) | 27.5(25.2-31.3) | 0.315 | 27.5(25.1-31.7) | 27.3(25.0-31.2) | 0.513 |
| **Obesity(BMI≥30 kg/m^2^) n(%)** | 32% | 31% | 0.492 | 48(30%) | 23(28%) | 0.637 |
| **Type 2 diabetes mellitus n(%)** | 21%) | 21% | 0.899 | 20% | 20% | 0.713 |
| **Hypertension n(%)** | 58% | 56% | 0.921 | 55% | 52% | 0.815 |
| **Chronic lung disease**  **COPD**  **Asthma** | 15%  2% | 17%  3% | 0.497  0.581 | 15%  3% | 19%  4% | 0.359  0.416 |
| **Coronary heart disease n(%)** | 5% | 7% | 0.592 | 8% | 8% | 0.873 |
| **Chronic atrial fibrillation n(%)** | 3% | 4% | 0.839 | 3% | 4% | 0.693 |
| **Chronic kidney disease n(%)** | 10% | 9% | 0.763 | 10% | 8% | 0.735 |
| **History of cancer n(%)** | 2% | 3% | 0.516 | 2% | 4% | 0.731 |
| **Immunocompromised state*** | 1% | 4% | 0.812 | 3% | 4% | 0.813 |
| **Smoking status:**  **former(%)**  **current(%)** | 13%  8% | 16%  8% | 0.394  0.478 | 11%  8% | 15%  9% | 0.313  0.411 |
| **ISARIC 4 C mortality score** | 14(10-15) | 14(12-16) | 0.591 | 14(10-15) | 14(11-15) | 0.536 |
| **SAPS II score** | 35(31-39) | 35(32-39) | 0.839 | 36(31-39) | 35(32-40) | 0.727 |
| **Temperature(°C)** | 36.5(36-36.9) | 36.4(36-36.8) | 0.348 | 36.5(36-36.9) | 36.4(36-36.8) | 0.821 |
| **Sys BP (mmHg)** | 130(120-140) | 130(116-140) | 0.413 | 130(120-140) | 130(116-140) | 0.767 |
| **Dia BP (mmHg)** | 74(65-80) | 75(68-80) | 0.592 | 74(65-80) | 75(68-80) | 0.839 |
| **Heart Rate (beats/min)** | 85(71-96) | 81(70-99) | 0.637 | 85(71-96) | 81(70-99) | 0.744 |
| **Respiratory rate(breaths/min)** | 28(21-30) | 28(23-30) | 0.916 | 28(21-30) | 28(23-30) | 0.953 |
| **PaO2/FiO2 ratio** | 100(80-137) | 102(80-140) | 0.479 | 102(78-140) | 104(80-142) | 0.539 |
| **PaO2/FiO2 ratio category n(%) patients**  **150-199**  **100-149**  **<100** | 18%  32%  50% | 18%  32%  50% | 0.999  0.891  0.713 | 15%  35%  50% | 19%  31%  50% | 0.713  0.814  0.358 |
| **paCO2 (mmHg)** | 34(31-37) | 35(31-39) | 0.892 | 34(31-37) | 35(31-39) | 0.484 |
| **paCO2>45 mmHg at admission n(%)** | 1 | 1 | 0.916 | 6 | 5 | 0.769 |
| **Arterial pH** | 7.47(7.43-7.49) | 7.46(7.44-7.49) | 0.872 | 7.47(7.43-7.49) | 7.46(7.44-7.49) | 0.739 |
| **Main efficacy outcomes** |  | | | | | |
| **NIV failure** | 45% | 15% | 0.0002 | 48% | 7% | 0.001 |
| **Death** | 38% | 11% | 0.0001 | 36% | 6% | 0.01 |
| **ETI** | 30% | 10% | 0.004 | 36% | 2% | 0.03 |
| **Safety end-points** |  | | | | | |
| **back pain** | 9% | 10% | 0.416 | 7% | 10% | 0.418 |
| **intravenous/arterial line dislodgement** | 6% | 6% | 0.639 | 6% | 6% | 0.397 |
| **haemodinamic instability** | 0% | 0% | 0.791 | 0% | 0% | 0.498 |
| **Barotrauma †**  **Pneumothorax/**  **Pneumomediasinum**  **Subcutaneous emphysema** | 2%  1%  3%  2% | 3%  0%  4%  3% | 0.713  0.515  0.413  0.398 | 3%  1%  3%  2% | 4%  0%  5%  5% | 0.682  0.779  0.319  0.438 |
| **Gastric distension and vomiting** | 0% | 0% | 0.999 | 0% | 0% | 0.999 |
| **device-related: nasal skin ulceration** | 2% | 2% | 0.612 | 0% | 0% | 0.313 |
| **Facial oedema** | 3% | 6% | 0.498 | 2% | 5% | 0.513 |
| **Thoraco-abdominal wall haematoma** | 0%) | 0% | 0.999 | 1% | 3% | 0.291 |
| **Venous thrombosis**   - **upper limb**   **lower limb** | 3%  0 | 4%  0 | 0.698  0.892  0.999 | 4%  0 | 5%  0 | 0.813  0.771 |
| **Subintensive Care Unit-acquired Infection** | 7% | 9% | 0.445 | 6% | 7% | 0.398 |
| **excessive sedation^§^** | 1% | 1% | 0.999 | 1% | 0% | 0.781 |
| **Acute kidney injury requiring renal replacement therapy** | 1% | 1% | 0.999 | 1% | 1% | 0.818 |
| **Liver failure** | 0% | 0% | 0.999 | 0% | 0% | 0.981 |
| **Need for emergency ETI** | 0% | 0% | 0.999 | 0% | 0% | 0.999 |
| **Time to NIV failure(d)** | 5(3, 9) | 9(5, 12) | 0.879 | 5(3, 9) | 9(6, 12) | 0.489 |
| **Time to death(d)** | 8(6, 11) | 14(10, 16) | 0.398 | 8(6, 11) | 14(10, 16) | 0.312 |
| **Time to ETI(d)** | 5(2,8) | 9(4, 10) | 0.297 | 5(2,8) | 9(4, 10) | 0.298 |

**Supplementary Table 14.** Baseline and main efficacy and safety outcomes of included patients, grouped according to the use of sedation with dexmedetomidine (n=243).

|  | **Sedation** | | | **No sedation** | | |
| --- | --- | --- | --- | --- | --- | --- |
| **Parameter** | **Controls**  **(n=56)** | **PP**  **(n=55)** | **P** | **Controls**  **(n=106)** | **PP**  **(n=26)** | **P** |
| **Age(yr)** | 72(68-71) | 74(66-79) | 0.715 | 69(69-78) | 68(60-75) | 0.813 |
| **Male sex(n, %)** | 79% | 72% | 0.516 | 72% | 76% | 0.829 |
| **Race**  **White, non-hispanic**  **White, hispanic**  **Black** | 91  6  3 | 95  3  2 | 0.815  0.717  0.629 | 155  6  1 | 75  4  2 | 0.498  0.717  0.626 |
| **Time from symptom onset to hospital admission(d)** | 7(5-10) | 7(5-10) | 0.348 | 7(5-10) | 7(5-10) | 0.538 |
| **Time from hospital admission to enrollment(d)** | 2(1-3) | 2(1-3) | 0.857 | 2(1-3) | 2(1-3) | 0.912 |
| **BMI(kg/m^2^)** | 27.6(25.4-31.5) | 27.5(25.2-31.3) | 0.315 | 27.5(25.1-31.7) | 27.3(25.0-31.2) | 0.513 |
| **Obesity(BMI≥30 kg/m^2^) n(%)** | 32% | 31% | 0.492 | 48(30%) | 23(28%) | 0.637 |
| **Type 2 diabetes mellitus n(%)** | 21%) | 21% | 0.899 | 20% | 20% | 0.713 |
| **Hypertension n(%)** | 58% | 56% | 0.921 | 55% | 52% | 0.815 |
| **Chronic lung disease**  **COPD**  **Asthma** | 15%  2% | 17%  3% | 0.497  0.581 | 15%  3% | 19%  4% | 0.359  0.416 |
| **Coronary heart disease n(%)** | 5% | 7% | 0.592 | 8% | 8% | 0.873 |
| **Chronic atrial fibrillation n(%)** | 3% | 4% | 0.839 | 3% | 4% | 0.693 |
| **Chronic kidney disease n(%)** | 10% | 9% | 0.763 | 10% | 8% | 0.735 |
| **History of cancer n(%)** | 2% | 3% | 0.516 | 2% | 4% | 0.731 |
| **Immunocompromised state*** | 1% | 4% | 0.812 | 3% | 4% | 0.813 |
| **Smoking status:**  **former(%)**  **current(%)** | 13%  8% | 16%  8% | 0.394  0.478 | 11%  8% | 15%  9% | 0.313  0.411 |
| **ISARIC 4 C mortality score** | 14(10-15) | 14(12-16) | 0.591 | 14(10-15) | 14(11-15) | 0.536 |
| **SAPS II score** | 35(31-39) | 35(32-39) | 0.839 | 36(31-39) | 35(32-40) | 0.727 |
| **Temperature(°C)** | 36.5(36-36.9) | 36.4(36-36.8) | 0.348 | 36.5(36-36.9) | 36.4(36-36.8) | 0.821 |
| **Sys BP (mmHg)** | 130(120-140) | 130(116-140) | 0.413 | 130(120-140) | 130(116-140) | 0.767 |
| **Dia BP (mmHg)** | 74(65-80) | 75(68-80) | 0.592 | 74(65-80) | 75(68-80) | 0.839 |
| **Heart Rate (beats/min)** | 85(71-96) | 81(70-99) | 0.637 | 85(71-96) | 81(70-99) | 0.744 |
| **Respiratory rate(breaths/min)** | 28(21-30) | 28(23-30) | 0.916 | 28(21-30) | 28(23-30) | 0.953 |
| **PaO2/FiO2 ratio** | 100(80-137) | 102(80-140) | 0.479 | 102(78-140) | 104(80-142) | 0.539 |
| **PaO2/FiO2 ratio category n(%) patients**  **150-199**  **100-149**  **<100** | 18%  32%  50% | 18%  32%  50% | 0.999  0.891  0.713 | 15%  35%  50% | 19%  31%  50% | 0.713  0.814  0.358 |
| **paCO2 (mmHg)** | 34(31-37) | 35(31-39) | 0.892 | 34(31-37) | 35(31-39) | 0.484 |
| **paCO2>45 mmHg at admission n(%)** | 1 | 1 | 0.916 | 6 | 5 | 0.769 |
| **Arterial pH** | 7.47(7.43-7.49) | 7.46(7.44-7.49) | 0.872 | 7.47(7.43-7.49) | 7.46(7.44-7.49) | 0.739 |
| **Main efficacy outcomes** |  | | | | | |
| **NIV failure** | 45% | 15% | 0.0002 | 48% | 7% | 0.001 |
| **Death** | 38% | 11% | 0.0001 | 36% | 6% | 0.01 |
| **ETI** | 30% | 10% | 0.004 | 36% | 2% | 0.03 |
| **Safety end-points** |  | | | | | |
| **back pain** | 9% | 10% | 0.416 | 7% | 10% | 0.418 |
| **intravenous/arterial line dislodgement** | 6% | 6% | 0.639 | 6% | 6% | 0.397 |
| **haemodinamic instability** | 0% | 0% | 0.791 | 0% | 0% | 0.498 |
| **Barotrauma †**  **Pneumothorax/**  **Pneumomediasinum**  **Subcutaneous emphysema** | 2%  1%  3%  2% | 3%  0%  4%  3% | 0.713  0.515  0.413  0.398 | 3%  1%  3%  2% | 4%  0%  5%  5% | 0.682  0.779  0.319  0.438 |
| **Gastric distension and vomiting** | 0% | 0% | 0.999 | 0% | 0% | 0.999 |
| **device-related: nasal skin ulceration** | 2% | 2% | 0.612 | 0% | 0% | 0.313 |
| **Facial oedema** | 3% | 6% | 0.498 | 2% | 5% | 0.513 |
| **Thoraco-abdominal wall haematoma** | 0%) | 0% | 0.999 | 1% | 3% | 0.291 |
| **Venous thrombosis**   - **upper limb**   **lower limb** | 3%  0 | 4%  0 | 0.698  0.892  0.999 | 4%  0 | 5%  0 | 0.813  0.771 |
| **Subintensive Care Unit-acquired Infection** | 7% | 9% | 0.445 | 6% | 7% | 0.398 |
| **excessive sedation^§^** | 1% | 1% | 0.999 | 1% | 0% | 0.781 |
| **Acute kidney injury requiring renal replacement therapy** | 1% | 1% | 0.999 | 1% | 1% | 0.818 |
| **Liver failure** | 0% | 0% | 0.999 | 0% | 0% | 0.981 |
| **Need for emergency ETI** | 0% | 0% | 0.999 | 0% | 0% | 0.999 |
| **Time to NIV failure(d)** | 5(3, 9) | 9(5, 12) | 0.879 | 5(3, 9) | 9(6, 12) | 0.489 |
| **Time to death(d)** | 8(6, 11) | 14(10, 16) | 0.398 | 8(6, 11) | 14(10, 16) | 0.312 |
| **Time to ETI(d)** | 5(2,8) | 9(4, 10) | 0.297 | 5(2,8) | 9(4, 10) | 0.298 |

**Supplementary Table 15.** Baseline features of controls at admission, grouped according to the presence/absence of complete LUS examination (n=162).

| **Parameter** | **Controls LUS not available (n=56)** | **Controls LUS available**  **(n=106)** | **P** |
| --- | --- | --- | --- |
| **Age(yr)** | 72(68-71) | 69(69-78) | 0.481 |
| **Male sex(n, %)** | 79% | 72% | 0.829 |
| **Race**  **White, non-hispanic**  **White, hispanic**  **Black** | 91  6  3 | 155  6  1 | 0.498  0.717  0.626 |
| **Time from symptom onset to hospital admission(d)** | 7(5-10) | 7(5-10) | 0.538 |
| **Time from hospital admission to enrollment(d)** | 2(1-3) | 2(1-3) | 0.912 |
| **BMI(kg/m^2^)** | 27.6(25.4-31.5) | 27.5(25.1-31.7) | 0.513 |
| **Obesity(BMI≥30 kg/m^2^) n(%)** | 32% | 48(30%) | 0.637 |
| **Type 2 diabetes mellitus n(%)** | 21%) | 20% | 0.713 |
| **Hypertension n(%)** | 58% | 55% | 0.815 |
| **Chronic lung disease**  **COPD**  **Asthma** | 15%  2% | 15%  3% | 0.359  0.416 |
| **Coronary heart disease n(%)** | 5% | 8% | 0.873 |
| **Chronic atrial fibrillation n(%)** | 3% | 3% | 0.693 |
| **Chronic kidney disease n(%)** | 10% | 10% | 0.735 |
| **History of cancer n(%)** | 2% | 2% | 0.731 |
| **Immunocompromised state*** | 1% | 3% | 0.813 |
| **Smoking status:**  **former(%)**  **current(%)** | 13%  8% | 11%  8% | 0.313  0.411 |
| **ISARIC 4 C mortality score** | 14(10-15) | 14(10-15) | 0.536 |
| **SAPS II score** | 35(31-39) | 36(31-39) | 0.727 |
| **Temperature(°C)** | 36.5(36-36.9) | 36.5(36-36.9) | 0.821 |
| **Sys BP (mmHg)** | 130(120-140) | 130(120-140) | 0.767 |
| **Dia BP (mmHg)** | 74(65-80) | 74(65-80) | 0.839 |
| **Heart Rate (beats/min)** | 85(71-96) | 85(71-96) | 0.744 |
| **Respiratory rate(breaths/min)** | 28(21-30) | 28(21-30) | 0.953 |
| **PaO2/FiO2 ratio** | 100(80-137) | 102(78-140) | 0.539 |
| **PaO2/FiO2 ratio category n(%) patients**  **150-199**  **100-149**  **<100** | 18%  32%  50% | 15%  35%  50% | 0.713  0.814  0.358 |
| **paCO2 (mmHg)** | 34(31-37) | 34(31-37) | 0.484 |
| **paCO2>45 mmHg at admission n(%)** | 2 | 3 | 0.713 |
| **Arterial pH** | 7.47(7.43-7.49) | 7.47(7.43-7.49) | 0.739 |
| **Main efficacy outcomes** |  |  |  |
| **NIV failure** | 45% | 48% | 0.001 |
| **Death** | 38% | 36% | 0.010 |
| **ETI** | 30% | 36% | 0.030 |
| **Safety end-points** |  |  |  |
| **back pain** | 9% | 7% | 0.418 |
| **intravenous/arterial line dislodgement** | 6% | 6% | 0.397 |
| **haemodinamic instability** | 0% | 0% | 0.498 |
| **Barotrauma †**  **Pneumothorax/**  **Pneumomediasinum**  **Subcutaneous emphysema** | 2%  1%  3%  2% | 3%  1%  3%  2% | 0.682  0.779  0.319  0.438 |
| **Gastric distension and vomiting** | 0% | 0% | 0.999 |
| **device-related: nasal skin ulceration** | 2% | 0% | 0.313 |
| **Facial oedema** | 3% | 2% | 0.513 |
| **Thoraco-abdominal wall haematoma** | 0%) | 1% | 0.291 |
| **Venous thrombosis**   - **upper limb**   **lower limb** | 1%  0 | 4%  0 | 0.912  0.898 |
| **Subintensive Care Unit-acquired Infection** | 7% | 6% | 0.398 |
| **excessive sedation^§^** | 1% | 1% | 0.781 |
| **Acute kidney injury requiring renal replacement therapy** | 1% | 1% | 0.818 |
| **Liver failure** | 0% | 0% | 0.981 |
| **Need for emergency ETI** | 0% | 0% | 0.999 |
| **Time to NIV failure(d)** | 5(3, 9) | 5(3, 9) | 0.489 |
| **Time to death(d)** | 8(6, 11) | 8(6, 11) | 0.312 |
| **Time to ETI(d)** | 5(2,8) | 5(2,8) | 0.298 |

**Supplementary Table 16.** Baseline features of controls at admission, grouped according to the date of enrolment: during the 1^st^ pandemic wave (i.e. from March 1^st^ to June 30^th^, 2020) or the 2^nd^ pandemic wave (i.e., during July 1^st^-Dec 15^th^, 2020) (n=162).

| **Parameter** | **Controls enrolled during April 1^st^-June 30^th^, 2020 (n=79)** | **Controls enrolled during July 1^st^-Dec 15^th^, 2020**  **(n=83)** | **P between control groups** |
| --- | --- | --- | --- |
| **Age(yr)** | 73(59-78) | 67(59-77) | 0.394 |
| **Male sex(n, %)** | 75% | 70% | 0.829 |
| **Race**  **White, non-hispanic**  **White, hispanic**  **Black** | 90  6  4 | 88  8  4 | 0.498  0.717  0.626 |
| **Time from symptom onset to hospital admission(d)** | 7(5-10) | 6(5-9) | 0.538 |
| **Time from hospital admission to enrollment(d)** | 2(1-3) | 2(1-3) | 0.912 |
| **BMI(kg/m^2^)** | 27.7(25.7-31.2) | 28.3(25.5-33.2) | 0.128 |
| **Obesity(BMI≥30 kg/m^2^) n(%)** | 29% | 38% | 0.396 |
| **Type 2 diabetes mellitus n(%)** | 17%) | 25% | 0.328 |
| **Hypertension n(%)** | 58% | 63% | 0.729 |
| **Chronic lung disease**  **COPD**  **Asthma** | 12%  2% | 13%  3% | 0.792  0.368 |
| **Coronary heart disease n(%)** | 6% | 9% | 0.713 |
| **Chronic atrial fibrillation n(%)** | 3% | 3% | 0.693 |
| **Chronic kidney disease n(%)** | 9% | 18% | 0.232 |
| **History of cancer n(%)** | 2% | 2% | 0.731 |
| **Immunocompromised state*** | 1% | 2% | 0.713 |
| **Smoking status:**  **former(%)**  **current(%)** | 18%  10% | 12%  9% | 0.313  0.411 |
| **ISARIC 4 C mortality score** | 13(10-15) | 13(11-15) | 0.629 |
| **SAPS II score** | 35(31-39) | 35(33-39) | 0.637 |
| **Temperature(°C)** | 36.4(36-36.9) | 36.6(36-36.9) | 0.821 |
| **Sys BP (mmHg)** | 130(120-140) | 130(120-140) | 0.682 |
| **Dia BP (mmHg)** | 74(65-80) | 74(65-80) | 0.839 |
| **Heart Rate (beats/min)** | 85(71-96) | 85(71-96) | 0.744 |
| **Respiratory rate(breaths/min)** | 26(20-30) | 28(22-30) | 0.496 |
| **PaO2/FiO2 ratio** | 103(80-134) | 97(78-130) | 0.318 |
| **PaO2/FiO2 ratio category % patients**  **150-199**  **100-149**  **<100** | 18%  31%  51% | 11%  31%  58% | 0.639  0.713  0.218 |
| **paCO2 (mmHg)** | 34(31-37) | 32(29-36) | 0.313 |
| **paCO2>45 mmHg at admission n(%)** | 2 | 2 | 0.713 |
| **Arterial pH** | 7.46(7.42-7.49) | 7.45(7.44-7.48) | 0.613 |
| **Adiuvant therapies** |  |  |  |
| **Steroids**  **Dexamethasone**  **-10 mg/d**  **-6 mg**  **Methylprednisolone 40 mg** | 100%  76(96%)  22 (28%)  44(56%)  3(4%) | 100%  81(97%)  16(19%)  74(89%)  2(2%) | 0.999  0.813  0.238  0.113  0.739 |
| **Remdesivir** | 0 | 1 | 0.7820. |
| **Tocilizumab** | 4 | 0 | 0.178 |
| **Convalescent plasma** | 0 | 1 | 0.981 |
| **Enoxaparin**  **Prophylactic dose**  **Intermediate dose***  **Anticoagulant dose** | 96%  33 (42%)  9 (11%)  33(43%) | 96%  35 (42%)  7 (8%)  38(46%) | 0.912  0.813  0.629  0.711 |
| **Warfarin/DOACs** | 4% | 4% | 0.713 |
| **Antibiotics**  **Azithromycin**  **Beta-lactams**  **Others** | 7(9%)  49(62%)  3(4%) | 4(5%)  51(61%)  2(2%) | 0.415  0.803  0.769 |
| **Antifungal** | 2(1%) | 1(1%) | 0.694 |
| **Any SARS-CoV-2 vaccine** | 0(0%) | 0(0%) | 0.999 |
| **Ventilatory mode at baseline**  **CPAP**  **PSV** | 54%  46% | 52%  48% | 0.912 |
| **Change in ventilatory mode**  **From CPAP to PSV**  **From PSV to CPAP** | 11%  8%  3% | 13%  5%  8% | 0.911  0.526  0.395 |
| **Interface at baseline**  **Face mask**  **Helmet** | 65%  35% | 67%  33% | 0.315  0.591 |
| **PEEP(cmH2O)**  **Baseline**  **Face mask**  **Helmet**  **During the study**  **Face mask**  **Helmet**  **Change**  **Face mask**  **Helmet** | 7(6-8)  10(10-10)  9(7-10)  10(10-11)  2(1-3)  0(0-2) | 7 (6-7)  10(10-10)  9(8-10)  10(10-10)  2(1-3)  0(0-2) | 0.483  0.417  0.394  0.312  0.713  0.496 |
| **Pressure Support (PS) cmH2O during PSV***  **Baseline**  **Face mask**  **Helmet**  **During the study**  **Face mask**  **Helmet**  **Change**  **Face mask**  **Helmet** | 4(3-6)  8(7-9)  5(4-6)  8(8-8)  0/0-1)  0(0-1) | 4(3-5)  8(7-9)  4(3-5)  8(8-8)  0(0-1)  0(0-2) | 0.394  0.789  0.394  0.595  0.375  0.291 |
| **Efficacy outcomes** |  |  |  |
| **NIV failure** | 33 (42%¶) | 37 (45%¶) | 0.412 |
| **Death** | 27 (34%)¶ | 32 (38%)¶ | 0.296 |
| **ETI** | 19 (28%)# | 23 (32%)# | 0.472 |
| **Days of invasive mechanical ventilation (d)** | 9(3, 15) | 5((2, 14) | 0.318 |
| **Death in invasively ventilated patients** | 13(68%) | 14(61%) | 0.438 |
| **Safety end-points** |  |  |  |
| **back pain** | 7% | 9% | 0.392 |
| **intravenous/arterial line dislodgement** | 8% | 5% | 0.491 |
| **haemodinamic instability** | 0% | 0% | 0.713 |
| **Barotrauma †**  **Pneumothorax/**  **Pneumomediasinum**  **Subcutaneous emphysema** | 1%  0%  0%  1% | 3%  1%  3%  2% | 0.312  0.591  0.369  0.397 |
| **Gastric distension and vomiting** | 0% | 0% | 0.999 |
| **device-related: nasal skin ulceration** | 2% | 1% | 0.391 |
| **Facial oedema** | 2% | 2% | 0.713 |
| **Thoraco-abdominal wall haematoma** | 0%) | 1% | 0.291 |
| **Venous thrombosis**   - **upper limb**   **lower limb** | 2%  0 | 3%  0 | 0.712  0.313 |
| **Subintensive Care Unit-acquired Infection** | 5% | 8% | 0.394 |
| **excessive sedation^§^** | 1% | 1% | 0.913 |
| **Acute kidney injury requiring renal replacement therapy** | 1% | 1% | 0.818 |
| **Liver failure** | 0% | 0% | 0.981 |
| **Need for emergency ETI** | 0% | 0% | 0.999 |
| **Time to NIV failure(d)** | 4(3, 7) # | 5(3, 9) * | 0.314 |
| **Time to death(d)** | 8(6, 12) # | 8(6, 10) # | 0.312 |
| **Time to ETI(d)** | 5(2,8) * | 6(3,8) * | 0.149 |

**Abbreviations:** ETI: endotracheal intubation; NIV: noninvasive ventilation;

*prophylactic dose twice daily

**Supplementary Table 17.** O2-responders, CO2-responders and NIV failure, death and ETI (%) in the PP group according to different definitions(n=70).

| **O2 response** | | | | |
| --- | --- | --- | --- | --- |
| **Definition** | **% responders**  **(PP group)** | **NIV failure**  **(% responders in the PP group)** | **ETI**  **(% responders in the PP group)** | **Death**  **(% responders in the PP group)** |
| **paO2/FiO2sp0-1>0** | 69%* | 16%# | 14%# | 13%# |
| **paO2/FiO2(pp1-sp0)≥20 mmHg** | 82%¶ | 21* | 20# | 18# |
| **paO2/FiO2(pp1-sp0)>20%** | 73%* | 16# | 14# | 13# |
| **paO2/FiO2(pp1-sp0)>10%** | 83% | 21* | 20# | 18# |
| **CO2 response** | | | | |
| **Definition** | **% responders**  **(PP group)** | **NIV failure**  **(% responders in the PP group)** | **ETI**  **(% responders in the PP group)** | **Death**  **(% responders in the PP group)** |
| **VRsp0-1>0** | 66%¶ | 11%¶ | 10%¶ | 9%¶ |
| **VRpp1-sp0 >0** | 70% | 13 | 11 | 10 |
| **MV_corr_sp0-1>0** | 66%¶ | 11%¶ | 10%¶ | 9%¶ |
| **MV_corr_pp1-sp0 >0** | 70%¶ | 13¶ | 11¶ | 10¶ |

The reference population is the control group; the reference timepoints are sp1-sp0 values for both O2 response and CO2 response (as in the main analysis).

* P<0.05 vs. treatment failure within quartile # P<0.001 vs. controls ¶ P<0.0001 vs. controls

**Supplementary Table 18.** Baseline and main efficacy and safety outcomes of included patients, after exclusion of patients with paCO2>45 mmHg at admission (n=225).

| **Outcome** | **Controls**  **(n=147)** | **Prone position**  **(n=78)** | **Absolute or mean difference (95% CI)** | **HR**  **(95% CI)** | **P** |
| --- | --- | --- | --- | --- | --- |
| **Primary outcome** | | | | | |
| **NIV failure at 28 d** | 62(41%) | 13(16%) | -25%  (-17%, -42%) | 0.32  (0.20, 0.51)   \|  \| \| --- \| \|  \| | <0.001 |
| **Secondary outcomes** | | | | | |
| **Death at 28 d** | 55(36%) | 9(11%) | -25%  (-15%, -40%) | \|  \| \| --- \| \|  \|   0.25  (0.15, 0.42) | <0.001 |
| **ETI at 28 d*** | 34(27%) | 8(10%) | -22%  (-13%, -39%) | 0.32(0.18-0.59)   \|  \| \| --- \| \|  \| | 0.002 |
| **Length of stay in**  **Subintensive Care Unit(d)** | 7(5,9) | 6(5,8) | -1(-2, 0) |  | **0.045** |
| **Days of invasive mechanical ventilation(d)** | 8(2, 15) | 6(2, 12) | -1(-2, 2) |  | 0.630 |
| **Death in invasively ventilated mechanically ventilated patients** | 25(74%) | 3(35%) | -39%(-18% to -84%) | 0.27(0.12-0.55) | **0.009** |
| **Length of hospital stay(d):**  **-whole study population**  **-hospital survivors** | 16(12,20)  19(15, 22) | 15(10,20)  15(10, 20) | 0(-1, 0)  -3(-5, -1) |  | 0.141  **0.039** |
| **Daily hours of NIV** | 20.3(15.9, 22.2) | 20.1(18.2, 22.4) | -1(0, -1) |  | **0**.812 |
| **Total days of NIV at 28 days** | 7(5,9) | 6(5,8) | -1(-2, 0) |  | **0.045** |
| **Daily hours of PP** | - | 12.2(10.1, 13.8) | - |  |  |
| **Duration of the longest PP session each day** | - | 10.9(9.1, 13.3) | - |  |  |
| **Number of PP sessions each day** | - | 2(1, 3) | - |  |  |
| **Total days of PP therapy at 28 days** | - | 6(5, 8) | - |  |  |
| **Device-related discomfort(NRS) at 28 days**  **Baseline**  **During the study** | 2(1, 2)  2(1,2) | 2(1, 2)  2(1,2) | 0(0, 1)  0(0,1) |  | 0.461  0.716  0.812 |
| **Dyspnoea (CPOT) at 28 days**  **Baseline**  **During the study** | 3(2,4)  1(0, 3) | 3(2,4)  0(-4, 1) | 0(0, 1)  -2(-3, 0) |  | 0.489  **0.009** |
| **Safety endpoints** |  |  |  |  |  |
| **back pain** | 14(9%) | 10(12%) |  |  | 0.479 |
| **intravenous/arterial line dislodgement** | 8(5%) | 4(5%) |  |  | 0.812 |
| **haemodinamic instability** | 0(0%) | 0(0%) |  |  | 0.919 |
| **Barotrauma** †  **Pneumothorax/**  **Pneumomediasinum**  **Subcutaneous emphysema** | 4(3%)  1(1%)  4(3%)  4(2%) | 4(4%)  0(0%)  4(5%)  3(4%) |  |  | 0.883 |
| **Gastric distension and vomiting** | 0(0%) | 0(0%) |  |  | 0.791 |
| **device-related: nasal skin ulceration** | 3(2%) | 2(2%) |  |  | 0.735 |
| **Facial oedema** | 5(3%) | 6(7%) |  |  | 0.771 |
| **Thoraco-abdominal wall haematoma** | 2(1%) | 3(3%) |  |  | 0.819 |
| **Venous thrombosis**   - **upper limb**   **lower limb** | 5(3%)  0 | 4(5%)  0 |  |  | 0.591 |
| **Subintensive Care Unit-acquired Infection** | 12(7%) | 8(10%) |  |  | 0.539 |
| **excessive sedation^§^** | 1(1%) | 1(1%) |  |  | 0.998 |
| **Acute kidney injury requiring renal replacement therapy** | 1(1%) | 1(1%) |  |  | 0.896 |
| **Liver failure** | 0(0%) | 0(0%) |  |  | 0.999 |
| **Need for emergency ETI** | 0(0%) | 0(0%) |  |  | 0.999 |
| **Time to NIV failure(d)** | 4(2, 8) | 9(5, 13) |  |  | **0.020** |
| **Time to death(d)** | 8(6, 11) | 14(10, 16) |  |  | **0.013** |
| **Time to ETI(d)** | 4(2,8) | 9(4, 10) |  |  | **0.028** |
| **Reason for ETI**† |  |  |  |  |  |
| **Worsening or nonimproving hypoxemia** | 44(32%) | 8(10%) |  |  | **0.017** |
| **Respiratory muscle fatigue** | 22(16%) | 6(7%) |  |  | **0.030** |
| **Worsening or unbearable dyspnoea** | 40(25%) | 4(5%) |  |  | **0.028** |
| Intolerance to treatment | 4(2%) | 1(1%) |  |  | 0.314 |
| Altered mental status | 1(1%) | 0(0%) |  |  | 0.813 |
| Shock | 0(0%) | 0(0%) |  |  | 0.999 |
| Hypercapnia | 1(1%) | 0(0%) |  |  | 0.914 |
| Inability to clear secretions | 1(1%) | 0(0%) |  |  | 0.892 |
| **Extracorporeal membrane Oxygenation** | 1(1%) | 0(0%) |  |  | 0.713 |

|  |  |  |  |  |  |  |  |
| --- | --- | --- | --- | --- | --- | --- | --- |
|  |  |  |  |  |  |  |  |
|  |  |  |  |  |  |  |  |
|  |  |  |  |  |  |  |  |
|  |  |  |  |  |  |  |  |
|  |  |  |  |  |  |  |  |
|  |  |  |  |  |  |  |  |
|  |  |  |  |  |  |  |  |
|  |  |  |  |  |  |  |  |
|  |  |  |  |  |  |  |  |
|  |  |  |  |  |  |  |  |
|  |  |  |  |  |  |  |  |
|  |  |  |  |  |  |  |  |
|  |  |  |  |  |  |  |  |
|  |  |  |  |  |  |  |  |
|  |  |  |  |  |  |  |  |
|  |  |  |  |  |  |  |  |
