# Supplementary material for: Early prolonged prone position in noninvasively ventilated patients with SARS-CoV-2-related moderate-to-severe hypoxemic respiratory failure: clinical outcomes and mechanisms for treatment response in the PRO-NIV study
Source: Crit Care. 2022 Apr 29;26:118. doi: 10.1186/s13054-022-03937-x (PMC9052189; doi:10.1186/s13054-022-03937-x)
Supplement: Supplementary file 3 — Additional file 3: Supplementary protocol. [file 13054_2022_3937_MOESM3_ESM.docx]

**Supplementary protocol**

**Early prolonged prone position in noninvasively ventilated patients with SARS-CoV-2-related moderate-to-severe hypoxemic respiratory failure: clinical outcomes and mechanisms for treatment response in the PRO-NIV study**

**Protocol version 1.2 Dec 15th, 2020**

**1.Background and Rationale**

**1.1 Prone position: physiological effects and clinical benefits in invasively ventilated**

**patients with acute respiratory distress syndrome (ARDS).**

**1.2 Prone position: rationale for COVID-19 patients with severe pneumonia and**

**preliminary case series**

**2. Research Hypothesis and Aims**

**3. Study Design setting and timeline**

**4. Methods**

**4.1 Eligibility criteria**

**4.2 Inclusion criteria**

**4.3 Exclusion criteria**

**4.4 Recruitment**

**4.5 Intervention**

**4.5.1 Experimental arm (prolonged prone position and NIV)**

**4.5.2 Matching controls (NIV delivered in supine position)**

**4.6 Equipment**

**4.7 Initial ventilator settings**

**4.8 Weaning criteria and procedure**

**4.9 Treatment failure criteria**

**5. Standard management:**

**5.1 Staff**

**5.2 Monitoring**

**5.3 Strategies to improve adherence to NIV and to prone position**

**5.4 Hemodynamic management**

**5.5 Nutrition**

**6. Study outcome measures: primary and secondary outcomes**

**7. Statistical analyses:**

**7.1 Power analysis**

**7.2 Methods to analyze primary and secondary outcomes**

**7.3 Methods to handle missing data**

**7.4 Methods to handle protocol non-adherence**

**7.5 Prespecified additional subgroup and sensitivity analyses**

**8. Ethics and Dissemination**

**8.1 Informed consent procedure**

**8.2 Blinding**

**8.3 Data collection and patient confidentiality,**

**8.4 Sponsorship**

**8.5 Regulatory and Ethics approval,**

**8.6 Record retention,**

**8.7 Competing interests,**

**8.8 Dissemination**

1. **Background and Rationale**

Severe Acute Respiratory Syndrome Corona Virus 2 (SARS‐CoV‐2) first appeared in Wuhan China in December 2019. It has since spread and was declared a worldwide pandemic by the World Health Organization in March 2020^[[1]](#endnote-1)^. Its main route of infection are respiratory droplets and contact transmission. Many infections are asymptomatic or mild, but 30% of Coronavirus disease 2019 (COVID-19) patients may develop severe pneumonia, with 60% of them requiring admission to Intensive Care Unit (ICU) and invasive mechanical ventilation.

Despite ongoing trials of antivirals and immunomodulatory therapies against COVID-19, the treatment of moderate/severe disease remains mainly supportive and mortality among invasively ventilated COVID-19 patients with severe respiratory failure remains overwhelmingly high, ranging 40-80%^[[2]](#endnote-2),^^[[3]](#endnote-3),^ ^[[4]](#endnote-4)^.

**1.1 Prone position: physiological effects and clinical benefits in invasively ventilated patients with acute respiratory distress syndrome (ARDS).**

Prone positioning (PP) therapy is a non-pharmacological treatment which has been shown to improve oxygenation through several mechanisms: improved ventilation/perfusion matching, relief of the compression of dependent lung regions from mediastinum's weight, and change in chest wall elastance^[[5]](#endnote-5),^^[[6]](#endnote-6)^.

Physiological studies have shown differences between prone and supine position in ventilation pressures in distinct regions of the chest: while breathing in a supine position, the ventral chest wall is lifted by a driving pressure driven by the difference between pleural pressure and atmospheric pressure (Ppl -Patm): the diaphragm moves caudally (Ppl-Pabdomen), and the dorsal chest wall moves minimally as lying in contact with a rigid surface. In the supine position, there is a reduction in alveolar size from sternum to vertebra at the end of the expiration.

This phenomenon, which has been clearly identified with CT scans leads to a greater expansion

of the nondependent regions and lower expansion of the dependent parenchyma^[[7]](#endnote-7),^^[[8]](#endnote-8)^.

On the opposite, while prone, the dorsal chest wall lifts, the diaphragm shifts similarly to supine position, and the ventral chest wall, now in contact with the firm surface of the bed, is impeded from expanding^8^. In the prone position the gravitational forces compress the ventral region, but this effect is damped by regional expansion due to shape matching between lung parenchyma and vertebrae. As the lung mass is anatomically greater in dorsal regions (nondependent when prone) than in ventral region (dependent when prone), the increased aeration and recruitment of the dorsal region tends to exceed the decreased aeration and de-recruitment of the ventral regions. This generates a more homogenous ventilation^[[9]](#endnote-9)^ across the entire lung, resulting in a reduction in transpulmonary pressure (Ptp, defined as the difference between the airway pressure, Paw, and pleural pressure, Ppl: Ptp = Paw–Ppl).

Furthermore, when an individual is supine the heart compresses the medial posterior lung parenchyma and the diaphragm compresses the posterior‐caudal lung parenchyma, with the abdominal contents displacing the diaphragm cranially^[[10]](#endnote-10)^ . Compression by either the heart and/or the diaphragm aggravates dependent lung collapse in the supine position . During prone ventilation,

the heart becomes dependent, lying on the sternum, and the diaphragm is displaced caudally, both phenomena relieving medial‐posterior lung parenchyma compression^5,10^.

Further effects of prone position are both an improvement of ventilation/perfusion match and an increase in cardiac output: the latter is thought to be due to the effect of increased lung

recruitment and reduction in hypoxic pulmonary vasoconstriction, resulting in increased right

ventricular preload and decreased pulmonary vascular resistance and right ventricular afterload

^[[11]](#endnote-11),^^[[12]](#endnote-12)^. Furthermore, current evidence suggests PP may be of value even if there is no improvement in gas exchange^11,^^[[13]](#endnote-13),^^[[14]](#endnote-14),^^[[15]](#endnote-15)^ and PP applied for prolonged periods (i.e., at least 16 hours per day) has been shown to half 28‐day mortality in patients with severe ARDS^[[16]](#endnote-16),^.

On this basis, current guidelines recommend prolonged PP for all invasively ventilated patients with severe ARDS^[[17]](#endnote-17),^^[[18]](#endnote-18)^.

**1.2 Prone position: rationale for COVID-19 patients with severe pneumonia and preliminary case series**

Although PP is a low cost, low risk and widely available therapy, it is not a standard of care for awake patients with respiratory failure and is currently recommended only for invasively ventilated patients with moderate to severe ARDS of varying aetiology^17,^ ^[[19]](#endnote-19),^^[[20]](#endnote-20),^^[[21]](#endnote-21)^

Data regarding PP therapy in awake patients before COVID-19 pandemic are scarce. A retrospective study of 15 patients, showed this therapy combined with non-invasive ventilation was safe and significantly improved oxygenation, however this improvement was transient, as oxygenation returned to baseline at 6 h after prone sessions^[[22]](#endnote-22)^. Another prospective observational study from China including 20 patients with moderate to severe ARDS found that PP combined with high-flow nasal cannula oxygen (HFNO) or non-invasive ventilation, improved significantly the pO2/FIO2 ratio, and half of the patients avoided intubation. However, due to the design and low sample size of the study, this can only be taken as hypothesis-generating data^[[23]](#endnote-23)^.

Regarding the effect of PP therapy in SARS‐CoV‐2 pneumonia , several case series, and small observational studies^[[24]](#endnote-24),^^[[25]](#endnote-25)^ showed PP for short periods of time (i.e. < 3 hours/day) improves oxygenation in awake COVID-19 patients with acute respiratory failure due to SARS‐CoV‐2 pneumonia receiving supplemental oxygen or noninvasive continuous positive airway pressure (CPAP), but the durability of this effect after resupination was incostant and there was no evidence for a clinical benefit on hard outcomes, including intubation and mortality. Furthermore, the impact of the timeliness and different duration of prone positioning on oxygenation and clinical outcomes is unknown^[[26]](#endnote-26)^.

Therefore the question if PP therapy can yield clinical benefits and lower the requirement of invasive mechanical ventilation or mortality in acute respiratory failure due to severe SARS‐CoV‐2 pneumonia lies unanswered.

1. **Research Hypothesis and Aims**

We hypothesize that in patients with moderate-to-severe acute respiratory failure due to SARS‐CoV‐2 pneumonia who require noninvasive ventilation (NIV), the early use of prolonged (i.e., at least 8 consecutive hours/day) PP combined with NIV can reduce NIV failure, the need for intubation, and overall mortality.

Key secondary hypotheses include tolerability and safety of PP and the potential for PP to improve ultrasonographic severity of lung disease and systemic inflammatory and coagulative cascade activation, as compared with NIV delivered by conventional methods (i.e., with patients in a supine or lateral decubitus position).

1. **Study design, setting and timeline**

This single-center non-randomized controlled trial will compare consecutive patients with moderate-to-severe acute hypoxemic respiratory failure due to SARS‐CoV‐2 pneumonia treated with NIV (CPAP or Pressure Support Ventilation, PSV) and prolonged PP (experimental group) prospectively enrolled from Jan 1^st^, 2021 to June 30th, 2021 with a group of matched historical controls, constituted by consecutive patients with moderate-to-severe acute hypoxemic respiratory failure due to SARS‐CoV‐2 pneumonia treated with NIV (CPAP or PSV) delivered in the conventional way (i.e., in the supine or lateral decubitus), from April 1^st^, 2020 to Dec 15^th^ , 2020.

All consecutive patients are treated by Intensivists (Emergency Physicians and Anesthesiologists) and nurses at the HUMANITAS Gradenigo COVID Subintensive Care Unit.

This trial will be approved by local Ethic Committee and registered with ISRCTN registry.

**End of study**

Study will continue until 28 days after the last enrolled patient or in any case for 6 months until June 2022, depending on levels of enrolment.

1. **Methods**

**4.1 Eligibility criteria**

All consecutive adult patients with microbiologically proven SARS‐CoV‐2 pneumonia and moderate to severe hypoxemic respiratory failure (defined by a paO2/FiO2 ratio <200) admitted to HUMANITAS Gradenigo Subintensive Care Unit who require NIV are eligible for this trial. Eligibility criteria for potential participants will be the same for both experimental and control group and are described below.

**4.2 Inclusion criteria**

**1.** Presence of acute (i.e. symptom onset <14 days of hospital access) hypoxemic respiratory failure.

2. Confirmed severe SARS‐CoV‐2 pneumonia based on the Center for Disease Control guidelines: SARS‐CoV‐2 infection confirmed by PCR AND bilateral opacities on chest X-ray or CT scan not fully explained by effusions, lobar or lung collapse, or nodules, with sO2<90% in room air on pulse oxymetry.

4. Cardiac failure not the primary cause of acute respiratory failure.

5. Moderate to severe hypoxemia, defined by a PaO2/FiO2 ratio <200 mm Hg while receiving oxygen therapy through either a Venturi mask with FiO2 50% or a non-rebreather reservoir bag mask, with FiO2 estimated as 0.21+ oxygen flow rate in L/min×3^[[27]](#endnote-27)^. To this aim, patients are categorized into 3 mutually exclusive classes of respiratory failure severity using previous definitions based on degree of hypoxemia

-mild: 200 mmHg ≤ PaO2/FIO2 < 300 mmHg;

-moderate: 100 mmHg ≤ PaO2/FIO2 < 200 mmHg;

-severe: PaO2/FIO2 ≤ 100 mmHg.

6. Able to provide informed consent

**4.3 Exclusion criteria**

1. Age less than 18 years-old

2. Pregnancy

3. Immediate need of invasive mechanical ventilation (altered mental status, fatigue, hemodynamic instability).

4. Contraindications for prone positioning therapy (recent abdominal or thoracic surgery

or wound; facial, pelvic or spine fracture.

5. Vomiting or bowel obstruction

6. Palliative care

7. Multiorgan failure

8. Pneumothorax

9. Inability of the patient to provide informed consent or uncooperativeness

10. Cardiac failure (history of chronic moderate to severe cardiac insufficiency (New York Heart Association class >II or left ventricular ejection fraction <50%), or acute exacerbation of chronic pulmonary disease as the primary cause of acute respiratory failure.

11. Prior treatment with NIV in the preceding month.

**4.4 Recruitment**

All consecutive patients with acute respiratory failure due to confirmed SARS‐CoV‐2 pneumonia needing noninvasive ventilation admitted to HUMANITAS Gradenigo Subintensive Care Unit since April 2020 will be screened by on-site critical care physicians.

The experimental and control arm will consist of:

-control arm: consecutive patients treated with noninvasive ventilation delivered in a conventional way (i.e., in supine or lateral decubitus position) from April 1^st^, 2020 to Dec 15^th^ , 2020 will be eligible to be included in the control arm

-experimental arm: all consecutive patients with acute respiratory failure due to SARS‐CoV‐2 pneumonia treated with NIV delivered with prolonged PP from Dec 16^th^ , 2020 to May 30^th^ , 2021, will be eligible for the experimental treatment in case of absence of exclusion criteria. Physicians will approach eligible patients within 24 hours of admission to Subintensive Care Unit and obtain written informed consent before treatment. Recruitment will end in any case on December 31^st^, 2021.

**4.5 Interventions**

In both arms, the duration of noninvasive ventilation is based on available literature and follows consolidated clinical practice^[[28]](#endnote-28),^^[[29]](#endnote-29)^ . These settings, along with other standards of care including timing and modalities for radiological and ultrasonographic imaging, nutrition, monitoring, treatment failure criteria, et other) were set early at the beginning of the pandemia in March 2020 and followed throughtout the study.

In both arms, patients receive NIV for at least 16 hours/day during at least the first 2 calendar days. Continuous NIV without interruptions in the first 24 hours of treatment is recommended. and

only brief interruptions are allowed for eventual adjustments, if needed, lasting no more than few minutes, immediately restarting NIV. During NIV interruption, patients will receive low flow oxygen therapy or nasal high flow oxygen therapy, according to physician’s decision.

Subsequently, NIV is reduced progressively in accordance with the degree of clinical improvement. Each patient is then evaluated daily without ventilatory support while breathing supplemental oxygen by a Venturi mask with a FIO2≤ 0.5 or nasal prongs with 10 l/m oxygen flow, for up to 2 hours. If the patients’ clinical status deteriorates, NIV is reapplied. Criteria for weaning and discontinuing NIV are described below.

- - 1. **Experimental arm (prolonged prone position and NIV)**

Patients of the experimental (prone position, PP) group will be treated with NIV (CPAP or Pressure Support Ventilation, PSV).

PP will be initiated early after admission to the Unit, after a brief (lasting 1-8 hours) period of NIV in the supine position, considered as baseline and lasting 1-to-8 hours. During this period an arterial line and eventually a central venous line and a nasogastric tube will be placed.

If NIV in supine position is not feasible for important desaturation, PP will be initiated immediately. In any case, PP therapy will be initiated within 24 hrs of admission to the Subintensive Care Unit.

Patients will be asked to remain in prone position (i.e. lying straight on the stomach, with the chest or the ventral side downwards and the back upwards; lateral decubitus or lying on their flank will not be considered prone position) throughout the day as long as possible, with at least 1 PP session lasting at least 8 hours each day. The mandatory 8-hr PP was scheduled overnight, could be extended daytime and/or integrated by additional daytime sessions according to patient tolerance and clinical judgement **(Figure 1).**

**Figure 1.** Overall study design with timing of prone position sessions, arterial blood gas analysis, blood chemistry and lung ultrasound.


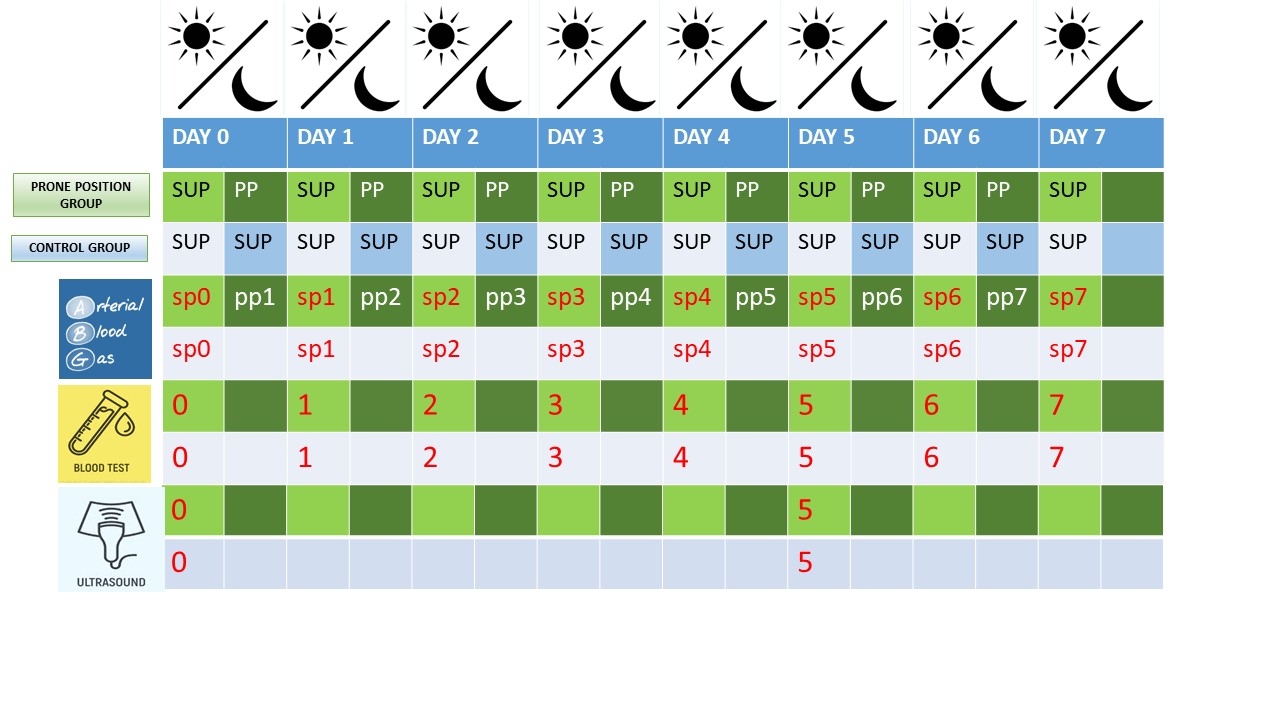


**Explanation.** The PP group had a mandatory night PP session of at least 8 hours; this session could be extended though the daytime or additional daytime sessions could be delivered according to clinical status and patient compliance. All ABGs were drawn during NIV, at least 1 hour after assuming PP or supine position

**Abbreviations:** ABG: arterial blood gas analysis; BC; blood chenistry: LUS: lung ultrasound; PP: prone position; SUP: supine position. The timepoints are indicated as follows:

sp0: supine position timepoint 0 (baseline, after NIV initiation)

pp1: during the first PP session in the PP group;

sp1: supine position timepoint 1 (after the initial 24 hrs of NIV; in the PP group this also corresponded to the resupination after the 1^st^ PP session)

pp2: during the 2^nd^ PP session

sp2: supine position timepoint 2 (after the 2^nd^ day of NIV, in the PP group this also corresponded to the resupination after the 2^nd^ PP session. Similar meaning for pp3, sp3,…

Patients completing at least one 8-hour proning session per day during the first 2 calendar days will be considered to have successfully completed PP therapy.

When considering a patient for PP treatment, the following guidance will be provided^[[30]](#endnote-30)^:

1. Preparation. At the time of consent, the procedure will be explained to the patient. Patients will be informed of the aim and potential benefits of PP and will be offered mild sedation and analgesia (see below) to enhance compliance to NIV and PP. At least 2 pillows, towels and blankets will be gathered, and at least 2 people beside the attending physicians will have to be present for assisance if required.
2. Position. First, the bed will be laid flat, then the patient will be asked to turn themselves onto their belly. A first pillow will be positioned under their chest or abdomen and a second pillow or a rolled towel under their forehead, leaving a gap to accommodate the eventual face mask. The patient will be asked to orient their head in whatever position they find most comfortable.
3. Oxygen and air supply and interface. There is no evidence as to which interface is best is patients receiving NIV in PP, so the choice will be left to the attending physician and to patient tolerability. In any case, the ventilator tubing need to be adjusted to be free at sight, and the interface (face mask or helmet) need not to be pushed against the patient face. This adjustments may require additional padding.
4. Position optimization. If required, additional pillows/bedding will be positioned to minimize pressure on body parts and to maximize patient comfort. The knees should be slightly flexed and the upper arm and shoulder should be kept in horizontal line. The arms will keep a comfortable angle, with the shoulder of the raised arm around 80° abduction and the elbow at an 80 degree flexion angle. Patient will be encouraged to reposition themselves when required or to call for help if they feel uncomfortable. A nurse call bell will be provided to each patient and kept within reach throughout the Subintensive Care Unit stay.
5. Monitor. In both arms, patient comfort pain and dyspnea (using the CPOT and NRS scale), GCS, SpO2, RR, HR, blood pressure, body temperature, ECG are continuously monitored by nurse staff and recorded on a predefined form (**Appendix A**)

Daily breaks, lasting no more than 2 hours, in PP will be allowed for meals and nursing care, according to patient tolerance.

In both arms, NIV in the original position will be resumed 1 hour after oral feeding in the supine position.

Termination of PP procedure will be considered whether the patient mantains all of the following conditions in the supine position, for at least 2 hours after terminating the last PP session:

-PaO2/FiO2 >300 with FiO2≤40%, and respiratory rate≤24/min during NIV.

-SpO2≥92% with FiO2≤40% via Venturi mask or with oxygen 10 l/m via nasal cannula and RR ≤24/min and no signs of altered respiratory mechanics.

In case of patients with severe COPD on long-term oxygen therapy, a lower oxygenation threshold

(PaO2/FIO2 >200 mmHg or SpO2≥90% with FiO2≤50% via Venturi mask or with oxygem 12 l/m via nasal cannula), together with the absence of decompensated respiratory acidosis (pH<7,35 on ABG), will be allowed to attempt weaning.

Proning procedure will be resumed if patient’s clinical or oxygenation status deteriorate.

**4.5.2 Matching controls (NIV delivered in a supine position)**

The control patients will be selected among consecutive patients with acute respiratory failure due to severe SARS‐CoV‐2 pneumonia treated in the HUMANITAS Gradenigo Subintensive Care Unit with NIV (CPAP or PSV) delivered in the conventional way (supine position or lateral decubitus) from April 1^st^, 2020 to Dec 16th, 2020.

The ratio will be 2 controls-to-1 experimental treatment patient.

All controls had the same enrollment criteria described for the experimental arm. The

physician who will make the selection will not know the results of the study and will not be informed about the evolution of the treatment.

For each patient with severe SARS‐CoV‐2 pneumonia treated with NIV and prolonged PP, two patients with moderate-to-severe acute hypoxemic respiratory failure due to SARS-CoV-2 pneumonia treated with NIV delivered in a conventional posture (supine or lateral decubitus) will be selected

To reduce the risk of bias due to unbalanced groups, propensity score (PS) analysis was performed to match PP and control group for the following baseline and treatment-related variables (see Statistics section):

- paO2/FiO2 ratio while receiving inspired oxygen by Venturi or reservoir mask on admission to the Subintensive Care Unit within the same category (paO2/FIO2 ratio of 150-199 or 100-149 or <100) of the PP patients and arterial pH within 0.04 of the values of the PP treatment patients.

-age

-BMI

-severity of illness on admission as assessed by the Simplified Acute Physiology Score (SAPS) II

-in-hospital mortality risk as assessed by the International Severe Acute Respiratory and Emerging Infections Consortium Coronavirus Clinical Characterization Consortium (ISARIC 4C) score^[[31]](#endnote-31)^,

-time from symptom onset to hospital admission

-time from hospital admission to NIV initiation

-pharmacological treatment with steroids, enoxaparin, remdesivir, tocilizumab

-ventilatory mode(CPAP vs. PSV)

In the control group, prone positioning could be considered as a rescue therapy after failure of ≥ 2 days of NIV delivered in the supine posture: in this case, the day of initiation and duration are recorded; these patients will remain in the control group for the main analysis (intention-to-treat analysis), but a sensitivity analysis after excluding patients who underwent rescue PP is planned (see Statistical analysis section).

**4.6 Equipment**

Pressure Support Ventilation is delivered by standard compressed gas ICU ventilators (Dräger Savina® 300 by Draeger, BPL Elisa 600 by Lowenstein Medical, Kronsaalsweg, Hamburg, Germany ) or turbine-powered air source ventilators (iVent 201 GE, Versamed Medical Systems. Haifa, Israel)

CPAP is delivered by ICU ventilator (Elisa 600 by Lowenstein Medical, Kronsaalsweg, Hamburg, Germany).

Interfaces will be full-face masks (PerfomrMax SAU W/SE, Philips Respironics, **Pennsylvania, United States)** and dedicated helmets for CPAP (CaStar, Starmed Intersurgical, UK) or for NIV (Dimar, Modena, Italy).

The mask will be secured with head straps, avoiding a tight fit, and the head of the bed is kept elevated at a 45° angle while the patient lies supine. A protective hydrocolloid sheet is applied over the forefront and chin, as needed. For patients with a nasogastric tube, a seal connector in the dome of the mask is used to minimize air leakage.

Helmet size is chosen according to neck circumference, as suggested by Antonelli et al.^28^  or according to manufacturer recommendations, if present.

The choice of the interface is left at the discretion of the attending physician and also depends on contingent device availability during COVID-19 pandemic.

**4.7 Initial ventilatory settings**

The choice of CPAP or PSV as initial ventilator mode, is left at the discretion of the attending physicians. Initial ventilator settings are chosen in the supine position and kept unaltered after proning the patient. The following initial settings are suggested:

**Face mask ventilation**

PEEP or CPAP was initially set at 5 cmH2O and increased in increments of 2–3 cm H2O up to 10-12 cm H2O to assure a peripheral oxygen saturation ≥92% at an inspired oxygen fraction (FIO2) of 50% or less, if possible. If CPAP was delivered by a ventilator, both flow and pressure triggers were adjusted at the most sensible value with no visible autotriggering (initial settings: pressure trigger set at -1 cm H2O and flow trigger at 0.5 L/sec).

Pressure support was applied and increased in increments of 2 to 3 cmH2O to obtain disappearance of signs of accessory muscle activity(as evaluated by palpating the sternocleidomastoid

muscle), a VTe≥0.6-0.8 ml/kg PBW, a respiratory rate of less than 30/min, and patient comfort. Ps \was also applied if in the presence of hypercapnia with respiratory acidosis (i.e. PaCO2>45 mmHg with pH<7.35). A maximum inspiratory time of 1.2 sec was set to prevent that unavoidable air leaks could interfere with flow-base cycling-off criteria, determining an unwarranted prolongation of mechanical insufflation during patient’s neural inspiration (inspiratory hang-up)^[[32]](#endnote-32)^.

**Helmet ventilation**

Patients ventilated with helmet also received pressure support ventilation or continuous positive airway pressure

**Helmet Pressure Support Ventilation.**

PSV is delivered through a dedicated helmet (Dimar, Modena, Italy)e via an ICU ventilator

The helmet, made of transparent latex-free polyvinyl chloride, was secured by padded armpit braces attached to 2 hooks on the front and back of a plastic ring connecting the helmet to a latex-free neck seal, thus producing a breathing circuit closed from the outside environment.

The helmet was connected to the ventilator by conventional respiratory circuitry joining 2 port sites to allow inspiratory and expiratory flow.

The ventilator will be set in PSV with the following initial suggested settings^[[33]](#endnote-33),^ ^[[34]](#endnote-34),^^[[35]](#endnote-35)^

1. initial pressure support≥8-10 cmH2O and adequate to permit a peak inspiratory flow of 100 l/min so as to avoid carbon dioxide rebreathing

2. positive end-expiratory pressure ≥10 cmH2O and increased to achieve the oxygenation target according to the choice of the attending physician.

3. FiO2 will be titrated to obtain an SpO2≥92%

4. Inspiratory flow trigger = 1 l/min to minimize inspiratory effort

5. fastest pressurization time(50 msec);

6. expiratory trigger: 10-50% of the peak inspiratory flow

7. maximum inspiratory time: 1.2 second

The points 5,6.7. are chosen to optimize patient-ventilator synchrony

The use of earplugs to mitigate noise-related discomfort will be allowed according to the decision of the attending physician and will be encouraged especially overnight.

**Helmet Continuous positive airway pressure (CPAP)**

CPAP was also delivered via through a dedicated latex-free polyvinyl chloride transparent helmet (CaStar, Starmed, Mirandola, Italy) with an initial PEEP of 10 cmH2O with a spring-loaded expiratory pressure valve (Vital Signs Inc, Totoma, NJ) applied.

The air flow was generated by an ICU ventilator delivering a continuous air flow of at least 50 L/min to minimize CO2 rebreathing^35^ or, when a standard ventilator was not available, through a flow generator with an adjustable inspiratory oxygen fraction set to deliver a flow of up to 140 L/min (Whisperflow, Caradyne, Ireland). using a Actual CPAP inside the helmet was measured through a dedicated manometer.

For NIV delivered by helmet, ventilatory pressure titration follows the same protocol suggested for mask ventilation.

Any modification in the ventilator settings and in the interface set-up to optimize comfort and patient-ventilator interaction will be allowed at the discretion of the attending physicians.

However, maintenance of PEEP ≥10 with helmet and ≥5 with face mask during the treatment is mandatory

**4.8 Weaning criteria and procedure**

Weaning from NIV within the first 48 hours of treatment will be discouraged.

In any case, weaning a patient from NIV at any time will be attempted only whether PaO2/FIO2 > 250 mmHg with FiO2≤50%, and respiratory rate≤24/min in the supine position for two consecutive days. In case of patients with severe COPD requiring long-term oxygen therapy, a lower PaO2/FIO2 threshold (PaO2/FIO2 > 200 mmHg with FiO2≤50%) is allowed, together with the absence of decompensated respiratory acidosis (pH<7,35 on ABG) was set to attempt weaning.

PEEP and Ps will then be lowered by 2 cmH2O at a time, keeping FiO2 unchanged. If the patient maintains SpO2≥95% with FiO2≤50% and respiratory rate≤24 during the following 60 minutes, NIV weaning will proceed by down-titrating PEEP and Ps by 2 cm H2O at a time every 60 minutes till reaching a PEEP of 5 cm H2O (with face mask) and 8 cmH2O (with helmet) and a Ps=2 cm H2O with face mask and 8 cm H2O with helmet.

If the patient maintains SpO2≥95% with FiO2≤50% and a respiratory rate≤24 in absence of dyspnea, activation of accessory muscles and paradoxic abdominal motion during the following 60 minutes, weaning will be considered successful.

After successful weaning from NIV and between two NIV sessions, patients will undergo low-flow or high flow nasal cannula oxygen set according to the choice of the attending physician to obtain the same SpO2 target.

NIV will be resumed at any time if the patient is experiencing respiratory distress, if the respiratory rate is more than 25/min and SpO2 is < 95% with FiO2 50% with and/or anytime deemed necessary by the attending physician.

All enrolled patients will be discharged from the Subintensive Care Unit while undergoing nasal prongs oxygen with an oxygen flow ≤10 l/min and a respiratory rate ≤ 24/min for at least 12 hours.

**4.9 Treatment failure criteria**

The ultimate decision to terminate NIV support and possibly intubate the patient will be made by 2 intensivists: the attending physician in conjunction with an experienced Anesthesiologist who is unaware of the study results. However, in order to avoid any delay in intubation, the following guidance was provided, based on available literature^28,29^ .

NIV termination and treatment failure should be considered in the presence of any of the following criteria:

(1) Signs of persisting or worsening respiratory failure, defined by at least two of the

following criteria:

- Respiratory rate above 40/min

-Lack of improvement of signs of respiratory-muscle fatigue

-Development of copious tracheal secretions

-Respiratory acidosis with a pH below 7.35

-SpO2 below 90% at FIO2 ≥ 0.8 for more than 1 hr or paO2/FiO2<100 for 1 hour

(2)intolerance to devices (helmet and full face mask)

(3) Hemodynamic instability defined by a SBP below 90 mmHg, MBP below 65 mmHg or

requirement for vasopressor;

(4) Deterioration of neurologic status (with a Glasgow coma scale below 12 points).

An Adjudication Committee constituted by two physicians with expertise in the field, not directly involved in the study and blinded to patients’ allocation, will verify whether the decision to terminate NIV support was unbiased and in compliance with the required criteria.

1. **Standard Management**

In both groups, standard care (monitoring, diagnostic tests, antibiotics administration, fluid therapy, haemodynamic management) will be applied according to the clinical practice at our Institution.

All patients will have a peripheral venous and arterial line inserted at entry in Subintensive Care Unit. The placement of a central venous line is advised to allow parenteral nutrition in case of continuous NIV: should a central venous line deemed necessary, the placement of a [peripherally inserted central catheter (PICC)](https://pubmed.ncbi.nlm.nih.gov/24811591/) by trained staff is recommended.

**5.1 Staff**

Nurse’s and physician’s ratio are staffed 1: 5 patients with a nurse assistant support. One another nurse in the shift supports the care of the patients, during the prone position patients and the hygiene care and therapy time. Nurses and physicians define together the better device for the patient and monitoring the compliance during the non-invasive ventilation.

**5.2 Monitoring**

-Continuous: spO2, electrocardiography, HR, RR. BP.

-vital signs, urine output, Richmond Agitation-Sedation Scale (RASS), ventilatory parameters(RR, FiO2, VTe), as well as patient comfort on the Numeric Rating Scale (NRS) for pain and CPOT for dyspnea are continuously monitored by nursing staff and recorded every 2-6 hrs. Patient’s posture (supine, lateral decubitus or prone) is continuously monitored each day and recorded hourly on a predefined form by nursing staff. Continuous diuresis monitoring will be at the discretion of the attending physicians. The form to record vital parameters is attached at the bottom of this document (Appendix A).

**5.3 Strategies to improve adherence to NIV and to prone position**

At the time of consent, patients eligible for the study will be informed of the rationale and aims of the prone position therapy and asked the permission to use mild pharmacological sedation to enable their compliance to prolonged PP therapy. In general, however, in both study groups, before considering sedation-analgesia to improve patient comfort to NIV, all factors known to improve NIV tolerance will be checked and optimized^[[36]](#endnote-36)^, including

1)ventilator settings

2)interface (type, size, and fit)

3)air leak control and patient-ventilator asynchrony containment

In either control or experimental group, should sedation-analgesia be required after NIV initiation to relieve patient discomfort and pain and enhance tolerance to NIV and/or to prolonged PP, the following guidance is provided.

| The short-acting alpha-2 adrenoreceptor agonist dexmedetomidine will be the sedative of choide for its minimal respiratory depression, its rapid onset and waining of action, and its anxiolytic and mild analgesic properties^[[37]](#endnote-37),^^[[38]](#endnote-38),^^[[39]](#endnote-39),^ ^[[40]](#endnote-40)^  Dexmedetomidine will be started at an velocoty of 0,2 ug/kg/h, without a loading dose, and gradually uptitrated by 0,2 ug/kg/h at a time every 30 min (till a maximum dose of 1,4 ug/kg/h) to keep the patient calm, arousable and comfortable and mantain a RASS score between 0 and -3.  Dexmedetominie will be stopped or downtitrated in case of profound bradycaria (HR≤40 bpm), hypotension (sysBP<90 mmHg) or deep sedation (RASS< -3). Dexmedetomidine will be downtitrated or interrupted between each proning session, and resumed at the beginning of the following PP session, if required.  The concomitant use of benzodiazepines with dexmedetomidine is discouraged^[[41]](#endnote-41)^, unless benzodiazepines were already part of patient’s home therapy; in these cases, the decision of adding short-acting benzodiazepines to dexmedetomidine can be considered.  For pain relief, acetaminophen ( 1 g bolus every 6-8 hours, up to 3-4 g/day) will be the first-line drug, followed by morhpine sulphate (3-5 mg bolus intravenous or subcutaneous every 6-8 hours up to 3-4 times/day) to obtain a NRS/CPOT score<3.  For dyspnea relief, morphine sulphate (3-5 mg bolus intravenous or subcutaneous every 6-8 hours) will be the drug of choice. The choice of an alternative opioid, i.e. fentanyl or remifentanyl continuous infusion, is left to the attending physicians^[[42]](#endnote-42)^. |
| --- |

If despite these measures the patient does not maintain PP or adverse events occur, PP therapy failure occurs.

**5.4 Hemodynamic management**

Fluid overload will be discouraged. If appropriate fluid challenge fails to restore adequate blood pressure and organ perfusion and if the patient is deemed not to be fluid-responsive, therapy with vasopressor agents will be started.

Severe haemodynamic instability, cardiac arrest, pneumothorax or any other adverse event possibly related or worsened by the assigned treatments will be recorded in the CRF.

Norepinephrine will be the first-choice vasopressor agent to correct fluid-resistant hypotension in septic shock.

Vasopressin use may be considered in patients with refractory shock despite adequate fluid resuscitation and high-dose conventional vasopressors

In patients developing documented cardiac failure, dobutamine, if not contraindicated, will be the first line agent to increase cardiac output. If used in the presence of low blood pressure and adequate preload(as estimated by central venous pressure) , it will be combined with vasopressor therapy.

**5.5 Nutrition**

All enrolled patients will be allowed and encouraged to drink and continue enteral feeding.

Daily caloric and protein requirements will be calculated following ESPEN practical guidance and patients managed accordingly^[[43]](#endnote-43)^

1. **Study outcome measures**

**Primary outcome measure**

The primary outcome will be the occurrence of NIV failure within 28 days of enrolment, defined as the occurrence of endotracheal intubation (for those patients with a full treatment indication) OR death for those patients with a Do-Not-Intubate (DNI) order.

**Secondary outcome measures**

The secondary outcomes, all censored at 28 days after enrolment will be:

**1)** Mortality

**2)** Need for endotracheal intubation among patients with a full treatment indication.

**3)** Time to NIV failure/intubation/death;

**4)** Patient device-related discomfort and dyspnoea. Device-related pain will be assessed via the Numeric Pain Rating Scale(NRS), dyspnoea will e assessed via the Critical-Care Pain Observation tool(CPOT). These will be assessed daily in both arms and additionally in the PP group 1 hour after assuming PP.

**5)**Daily hours of prone positioning

**6)** Hours of the longest prone session each day

**7)** Total number of prone sessions each day

**8)** Daily hours of NIV

**9)** Total days of prone positioning therapy

**10)** Total days of NIV

**11)**N-patients discharged from hospital

**12)** Length of stay in Subintensive Care Unit

**13)** Length of hospital stay (whole study population and hospital survivors) at 28 days

**14)** Days of invasive mechanical ventilation

**15)** Death in invasively mechanically ventilated patients;

**15)** Safety endpoints: in patients meeting the primary endpoint, the time and reason for NIV failure and for ETI and the proportion of patients requiring emergency ETI; the proportion of patients with a full treatment indication who died without ETI; the time to death; adverse effects of NIV/prone positioning therapy (skin ulceration/decubiti, intravenous/arterial lines dislodgement), barotrauma (pneumothorax, pneumomediastinum, subcutaneous emphysema), infections (at admission and Subintensive Care Unit-acquired), septic shock, liver failure, acute kidney injury requiring renal replacement therapy, upper limb thrombosis.

Additionally, we planned to assess the following outcomes during the initial 7 days after enrollment to explore physiological response to PP:

**1)** Change in oxygenation parameters: the main oxygenation parameter will be the paO2/FiO2 ratio, which will be obtained from ABG 1-hour after starting NIV in supine position and then at least daily in all patients. Additionally, in the PP group ABG will be obtained and the paO2/FiO2 ratio calculated, 1-hour after starting each prone session and 1-hour after resuming supine position.

Respiratory rate, VTe and MV will be recorded at the time of each ABG.

**2)** Change in indices of physiological dead space. We explored the effect of treatment on Ventilatory Ratio(VR) and on corrected Minute Ventilation (MV_corr_), two surrogate indices of physiological dead space.

VR strongly correlated with direct measures of dead space in patients receiving either mandatory or spontaneous invasive mechanical ventilation^[[44]](#endnote-44)^ and predicts adverse outcomes in ARDS independently of indexes of oxygenation^[[45]](#endnote-45)^. In invasively ventilated patients with SARS-CoV-2-related ARDS, increased VR has been related to mortality^[[46]](#endnote-46)^^[[47]](#endnote-47)^. MV_corr_ has been retrospectively associated with mortality in invasively ventilated patients with SARS-CoV-2-related ARDS^[[48]](#endnote-48)^. The validity of both these indices outside invasive lung-protective mechanical ventilation has not been assessed.

The hypothesis is that PP improves oxygenation by recruiting posterior consolidated lung regions rather than overdistending already aerated lung regions. To assess the impact of NIV delivered in supine or PP on lung anatomy, VR and MV_corr_ in supine and PP will be related to ultrasonographic lung imaging data (see below).

Ventilatory Ratio(VR)=measured minute ventilation(ml/min) × measured PaCO2(mmHg)/predicted minute ventilation(ml/min) × predicted PaCO2(mmHg), where minute ventilation=expiratory tidal volume(VTe) × respiratory rate.

Predicted minute ventilation is calculated as predicted body weight in kg × 100 (ml/min).

Predicted PaCO2 is the expected PaCO2 (37.5 mmHg) if the patient is ventilated with the predicted minute ventilation.

MV_corr_ (L/min)= MV  x PaCO_2_/(physiological PaCO_2_). Physiological PaCO_2_ is set at 40 mmHg.

Due to the inaccuracy of measured VTe with helmet, VR will be only calculated in patients ventilated with face mask and standard ICU ventilators. VR will be measured after each postural change at the time of ABG, once ventilatory stability is achieved (defined by a ≤10% variation in RR and VTe and air leaks <10% for at least 30 minutes).

Although VR and MV_corr_ were originally derived from invasively ventilated patients, it has been clearly demonstrated in computerized lung models and *in vivo* studies clearly demonstrated that even with full face masks the dynamic dead space is negligible during ventilation, due to the streaming effect of gas flow during NIV, making the issue of CO2 rebreathing negligible^[[49]](#endnote-49),^^[[50]](#endnote-50)^. VR is unitless; values greater than 1 suggest increased dead space.

**3)** Change in lung ultrasound (LUS) score assessed within 24 hrs of enrollment and at day 5 since enrollemnt.

Lung ultrasound will be performed within 24 hrs of enrollment before the initiation of NIV by three intensivists with expertise in lung and cardiac recording and interpretation (each operator having performed at least 50 supervised procedures and at least 200 non-supervised procedures^[[51]](#endnote-51)^) using the same equipment (HM70A Samsung, Seoul, Korea), the same convex-array probe and the same lung US setting. Each LUS will last between 10-15 min. In each subject, anterior and lateral lung regions will be scanned with the subject in the supine position, and the posterior region with the subject in a lateral or sitting position.

The severity and extent of parenchimal involvement of the 6 regions (2 anterior, 2 lateral, 2 dorsal) for each lung will be scored according to predefined criteria^[[52]](#endnote-52)^^[[53]](#endnote-53)^ and noted on a predefined form.

All adjacent intercostal spaces will be explored parallel and perpendicular to ribs. For each explored region, the worst finding and the LUS score will be rated as follows: 0=presence of lung sliding with A-lines or <3 isolated B-lines; 1=at least 3 well-separated B-lines; 2=multiple coalescent B-lines, ; 3= consolidation

Details about LUS examination are provided in the panel below

| **Lung ultrasound**. Examinations were performed by three intensivists with expertise in LUS recording and interpretation using the same equipment (CX 50, Philips Medical Systems, Bothell, WA), with the same cuvilinear probe and occasionally linear high-frequency probe. Each LUS lasted between 10-15 min, with the patient supine or semi-supine and sitting, omitting the need for position change during the examination.  A point scoring system was employed for each region and ultrasound pattern: A-lines (normal reverberation artifacts of the pleural line that when accompanied by lung sliding correspond to normal aeration of the lung) were equal to 0 point; B-lines (hyperechoic lines vertical to the pleura line, arising from it and reaching the edge of the screen erasing A-lines, which represent reverberation artifact through edematous interlobular septa or alveoli) were divided to B1 (separated B-lines that correspond to moderate lung aeration loss) that was equal to 1 point, and B2 (coalescent B-lines that correspond to severe lung aeration loss) that was equal to 2 points; Lung  consolidation that was equal to 3 points. Within each region, the probe had to be oriented both between intecostal spaces and transversally.  We also documented the presence of pleural thickening and defined a homogenous vs. patchy pattern of each examination. Pleural thickening was qualitatively determined, indicating  irregular pleural line either in cases of sub-pleural consolidations or in cases of B-lines accompanied by irregular pleural line. In accordance to present guidelines^[[54]](#endnote-54)^, the following measures were undertaken to minimize the risk of inadvertent infection: all studies were  performed bedside at the designated COVID-19 wards using dedicated scanners that were tagged and set aside in each ward. Full personal protection equipment was used and LUS measurements were performed offline to reduce exposure time.  In the end, an LUS score of 0 was normal, and 36 was worst.     |
| --- |

Global LUS score will correspond to the sum of the 12 regions’ score (range 0-36).

Additionally, the anterior LUS score (sum of the scores from the 2 anterior regions of each lung, ranging 0-12), lateral LUS score (sum of the scores of the 2 lateral regions of each lung, ranging 0-12) and posterior LUS score (sum of the scores of the 2 dorsal regions of each lung, ranging 0-12) will be calculated.

This lung scoring system has been shown to correlate with computed tomography (CT)-assessed severity of lung involvement and to predict clinical course and outcomes in COVID-19 patients^[[55]](#endnote-55),^^[[56]](#endnote-56),^ ^[[57]](#endnote-57)^.

The accuracy of LUS score in quantifying the extent and nature of lung disease will be internally assessed against a sample of at least 100 chest CT scans, performed the same day of LUS examination in patients with SARS-CoV-2 pneumonia-related acute respiratory failure admitted to the Subintensive Care Unit. All CT scans will be performed using a 128-slice CT (OPTIMA CT660, GE Healthcare, Chicago, Illinois, US) and will be contrast-enhanced, unless otherwise agreed between the attending physician and the radiologist on the basis of specific clinical reasons. The CT severity scoring system proposed and validated by Salaffi et al.^[[58]](#endnote-58)^, which takes into account both the extension and the nature of parenchymal involvement, will be used. Both the radiologist who scores the CT scans and the intensivist who performs the LUS exam will be blinded to the results of each other examination. The correlation between global LUS score and CT score will be evaluated with the Spearman correlation coefficient, with a 2-tailed P value < .05 considered statistically significant.

Follow-up LUS. There are no longitudinal data on the optimal timing for repeating lung ultrasound to monitor disease course and treatment effect in COVID-19-related pneumonia. Based on our preliminary experience made through daily LUS repetition in COVID-19 pneumonia patients treated with NIV, we will repeat lung ultrasound at day 5 since enrollment and their regional and global LUS score will be calculated. Additionally, the single region changes n LUS scores will be used to determine the LUS reaeration score as validated by Bouhemad and colleagues^[[59]](#endnote-59)^. The LUS reaeration score has been related to lung recruitment in invasively ventilated patients with COVID-19-related and COVID-19-unrelated ARDS^[[60]](#endnote-60),^ ^[[61]](#endnote-61)^.

The PEEP at which measurements will be made will be noted.

Cardiac ultrasound using the phased-array probe will be also performed by the 3 intensivists in all patients within 24 hr of enrollment and abnormalities in ventricle global kinesis and size are recorded. Lung and cardiac ultrasound will be repeated in case of clinical deterioration.

**4)** Change in 18 laboratory parameters recorded daily from admission to COVID Subintensive Care Unit until discharge. The laboratory parameters will include: serum C-reactive protein (CRP) and procalcitonin, total and differential blood cell counts, coagulative parameters (PT, PTT and plasma D-dimer measured by latex test), AST, ALT, lactate dehydrogenase (LDH), total and fractionated bilirubin, serum creatinine and BUN, fasting plasma glucose, CPK and hs-Tn I, Na, K, Mg.

1. **Statistical analyses**

**7.1 Power analysis**

Data from the Intensive Care National Audit & Research Centre (ICNARC) COVID-19 database^[[62]](#endnote-62)^ and from the largest literature series on noninvasively ventilated patients with hypoxemic respiratory failure due to severe SARS‐CoV‐2 pneumonia report a NIV failure of 50-60%^[[63]](#endnote-63)^, an intubation rate ranging 33-60%^[[64]](#endnote-64),^^[[65]](#endnote-65),^ ^[[66]](#endnote-66),^ ^[[67]](#endnote-67)^, and a mortality rate ranging 40-77%^21,^^[[68]](#endnote-68),^^[[69]](#endnote-69)^.

Assuming a 50% decrease in NIV failure, intubation rate and mortality to be clinically relevant, in a 1:2 ratio of experimental-to-control arm trial design and a minimal (i.e.<5%) loss at follow-up, a total of 180 patients (60 in experimental arm and 120 in conventional arm) would be needed to detect a statistically significant (p<0.05) difference between groups in NIV failure, with a beta error of 0.2, and alpha error of 0.05.

**7.2 Methods for primary and secondary outcomes**

Data will be analyzed at HUMANITAS Gradenigo by researchers with statistical expertise.

To reduce the risk of bias due to unbalanced groups, PS analysis will be performed through a logistic regression model adjusted for the baseline and treatment-related variables previously specified. We used a greedy nearest-neighbour matching algorithm with a 1:2 matching ratio and a caliper width≤0.2*SD^^[[70]](#endnote-70)^^.

Standardized mean differences (SMDs) between groups were calculated to assess balance in each baseline covariate, with absolute standardized differences<0.1 indicating adequate balance between groups.

All inferential analyses will be performed for all patients in the original cohort and for the propensity score-matched cohorts.

Definition of the two groups: The patients receiving NIV and the prone positioning are classified

as PP group. The patients receiving NIV in the conventional (supine or lateral decubitus) position are classified as control group.

Comparisons between the two groups: quantitative continuous variables are presented medians (with inter-quartile ranges) and are compared using the unpaired Student’s t test or the Mann-Whitney test. Normality was evaluated with the Shapiro-Wilk test. Qualitative or categorical variables are compared with the chi square test or the Fisher’s exact test, as appropriate.

To compare continuous variables collected at different time points(i.e., respiratory and biochemical parameters) we used repeated measures two-factor (within subject and between group) ANOVA for continuous variables assessed at multiple timepoints, after log-transformation of non-normal variables.

The cumulative probability of NIV failure, endotracheal intubation and of death at 28 days between the two groups will be compared with the Kaplan-Meier estimate of survival and the log-rank test.

We plan to use Cox proportional multivariable regression analysis to adjust for imbalanced variables between the two groups when assessing the effect of the interventions on NIV failure, endotracheal intubation and death in the whole study population at 28 days. Covariates or explanatory factors included in multivariate models were the variables associated to the binary response outcomes in the univariate analysis, with the maximum number of covariates to be included in the models set at (event rate x N)/10^[[71]](#endnote-71),^^[[72]](#endnote-72)^. Interactions between time and covariates will be also included.

We will run 2 Cox models: in the first, baseline predictors of clinical outcomes will be included.

In the second model, we will also include ventilatory and gas exchange parameters during the need the initial 2 days from enrollment to explore potential role of early pathophysiological predictors of clinical outcomes(NIV failure, death, ETI). We limited the timeframe to the initial 2 days for the following reasons:

-the need to investigate early predictors of treatment failure at early stages of treatment to avoid intubation delays

-for homogeneity of treatment of patients in the PP arm, who will all receive at least 2 mandatory sessions of PP therapy in the initial 2 days since enrollment;

- for predictability of treatment in both arms during the initial 2 days, during which patients will be ventilated almost uninterruptedly using preset ventilatory settings and interfaces, while thereafter setting and interfaces will be allowed to change on an individual patient setting.

In the pathophysiological model we used the following definitions:

-O2-responders: patients who increased the paO2/FiO2 ratio during NIV in supine position at day 1 as compared with NIV supine at day 0(i.e., pO2/FiO2sp2-pO2/FiO2sp1>0 or ΔpaO2/FiO2sp1-sp0>0).

-CO2-responders: an increase in CO2 clearance was defined by a reduction in dead space indices (VR and MVcorr, tested separately) during sp1 as compared with sp0) [ΔVR (sp1-sp0)<0); ΔMVcorr (PP1-sp0)<0)].

This allowed comparing gas exchange response between PP group and controls after 1 day of NIV, in the same (supine) position, and takes into account the effect of the first PP session in the PP group (as paO2/FiO2sp1 ABG will be drawn following resupination after the first PP session)25 (**Figure 1**).

As there is no uniformity in the literature as to the timepoint(whether during PP or after resupination) and the threshold to consider to define gas exchange response to PP ^15,^ ^[[73]](#endnote-73)^, we tested sensitivity of our results to different definitions of O2-responders and CO2-responders in a predefined sensitivity analysis(see Sensitivity analyses).

Predictors of continuous outcome variables, including ultrasonographic and blood-based parameters will be analyzed using linear univariable and multivariable regression analysis. Time change in continuous variables will be assessed by computing the area under the curve(AUC) was computed by the trapezoid method^[[74]](#endnote-74)^

In all multivariable models we will use a combination of backward procedure and exclusion of highly collinear variables through model-dependent Variance Inflation Factor(VIF) cut-off values to select covariates^[[75]](#endnote-75)^.

We will first calculate the VIFs for each effect in the usual manner by fitting the full model with all effects. Then, we will implement stepwise routine to remove variables causing loss of precision in parameter estimates by starting with the variable having the largest VIF. The first model-dependent cutoff value will thus set the limit by VIF(1)= 1/(1-R_1_^2^).

where R_1_^2^ is the coefficient of determination calculated by regressing the response on just that independent variable having the largest VIF in the full model.

If the VIF for that variable is less than VIF(1), then the collinearity among the independent variables will be considered small enough to be ignored and all of the variables will be kept in the model. If the VIF for that variable is greater than or equal to VIF(1), then the other independent variables in the model have a better explanatory power for this variable than this variable has for the response and the variable will be removed from the model.

We also plan to explore dose-response relationship between PP therapy and respiratory, utrasonographic and biochemical parameters in the PP group by univariable and multivariable regression analysis, after log transformation of skewed parameters.

We will search the best model fit among four predictive models (linear, exponential, logarithmic, binomial) using R-squared values.

All tests were performed at two-tails with significance set at a p value <0.05.

Data will be analyzed on an intention to treat basis with all data for patients who consented to PP (regardless of whether they successfully completed PP therapy or not) included in baseline data analysis. We planned a further per protocol analysis will be carried out on all patients who tolerated prone position for at least 8 hours/day for the first 2 days (successful PP therapy).

**7.3 Methods in analysis to handle missing data**

Missing data at random will be completed using last observation carried forward (LOCF) and the percentage of datasets with full or missing data will be reported. Missing data not-at random will not be imputed and excluded from the analysis if they miss due to the consequence of treatment effect(i.e. those patients who will be intubated or discharged from the Unit after successful weaning)

Sensitivity analyses will be made for the whole cohort and after excluding cases where LOCF data had to be imputed.

**7.4 Methods in analysis to handle protocol non-adherence**

In the prone position group (experimental group), PP failure will be defined by the inability to keep PP for at least 8 hours/day during the initial 2 study days. These patients will remain in the PP group, as all data will be analyzed on an intention-to-treat basis in the primary analysis.

A subsequent sensitivity analysis after excluding patients with PP failure is planned (see below)

In the control group, PP could be considered as a rescue therapy after failure of ≥2 days of NIV delivered in the supine position: these patients will remain in the control group, as all data will be analyzed on an intention-to-treat basis in the primary analysis. In this case, the day of initiation and duration are recorded for these patients and a sensitivity analysis is planned after excluding patients with rescue PP therapy.

**7.5 Prespecified subgroup and sensitivity analyses**

We planned the following subgroup analyses to assess the effect of the following characteristics on main clinical efficacy(NIV failure, death, ETI) and safety outcomes:

-severe (paO2/FiO2 at admission: <100) vs. moderate hypoxemic respiratory falure (paO2/FiO2 at admission: 100-199).

-per protocol analysis (patients with PP failure and rescue PP excluded),

-interface: helmet vs. face mask as initial ventilatory interface

-ventilatory mode: CPAP vs. Pressure Support Ventilation(PSV) as initial ventilatory mode

-sedation(yes/no),

-complete (pre/post) LUS examination available (yes/no)

--controls enrolled during the 1^st^ pandemic wave (March 1^st^-June 30^th^, 2020) vs. those enrolled during the 2^nd^ pandemic wave (after September 1^st^, 2020).

-different definition of O2-response and CO2-response in the PP group, based on gas exchange values during the first PP session (timepoint pp1) as compared with sp0 and on different thresholds for gas exchange responses^13,^ ^[[76]](#endnote-76)^:

•O2-responders: patients with an increase in the paO2/FiO2 ratio by ≥20 mmHg OR by >10% OR by >20% during the first PP session (timepoint pp1) as compared with the paO2/FiO2 ratio supine before the first PP session (ΔpaO2/FiO2pp1-sp0 ≥20 mmHg OR >10% OR >20%).

•CO2-responders: patients with a reduction in their VR during the first PP session as compared with the VR supine before the first PP session (ΔVRpp1-sp0 <0).

-after excluding patients with LOCF

-after excluding patients with PaCO2>45 mmHg at study inclusion

-for inflammatory and coagulative markers: after excluding patients treated with tocilizumab, with documented microbial infection and with venous thromboembolism, respectively, which may act as confounders on inflammatory and coagulative markers, respectively.

Furthermore, as we do not know a priori if death occurred prior to intubation in patients with a full treatment indication, we also plan to compare the risk of endotracheal intubation estimated with Cox proportional regression model with that estimated with the Fine-Gray model analyses for competing risk, with death prior to intubation for patients with a full treatment indication as a competing risk^[[77]](#endnote-77)^.

All analyses will be carried out with MedCalc 19.7 (MedCalc Software Ltd Ostend, Belgium) and STATISTICA 5.1 statistical software package(Statsoft, Tulsa, OK).

1. **Ethics and Dissemination**

**8.1 Informed consent procedure**

As patients will be self‐ventilating, Subintensive Care Unit physicians will obtain written informed consent or witnessed consent to reduce fomite transmission will be obtained for each patient enrolled in the study. A patient information leaflet will be given to all patients screened as eligible. After a period of time to read and consider the information leaflet time will be given for questions, and then if the patient consents to be involved, written consent will be obtained. Due to the risk of fomite transmission of SARS‐CoV‐2 a photo will be taken of the signature pages of the consent, and stored in a password protected encrypted database, stored under the title “PRONIV STUDY_CONSENT”. The original consent form will be included in the patients’ sanitary document.

**8.2 Blinding**

It is not feasible to blind staff or patients as to the procedure. Study data will be blinded for the purposes of analyses, assigned as group 1 or group 2 rather than prone / not proned.

**8.3 Data collection and patient confidentiality**

Data will be collected from patient paper sanitary record into an electronic case report form (CRF) consisting of an excel datasheet file, where each patient will be deidentified and coded by a unique, computer-generated number.

The CRF is enclosed with the protocol. No patient identifiers will leave hospital unit. Patient confidentiality will be maintained by keeping data collected in the study coded. The key will be at the HUMANITAS Gradenigo Hospital, Emergency Medicine and Surgery Department, and will be managed by the principal investigator. No Personal detail or identifying data will be transferred from the patient sanitary file to the CRF which will be available for data analysis.

**8.4 Sponsorship**

This study has no sponsors.

**8.5 Regulatory and ethics approval**

The trial was approved by the **Comitato Etico Interaziendale A.O.U.**Città della Salute **e** della Scienza di Torino and is registered with ISRCTN clinical trial registry(study ID: ISRCTN23016116).

**8.6 Record retention**

Participants are requested to give consent to store their data for 5 years (without this permission, patient cannot participate).

**8.7 Competing interest**

All participating investigators have no conflict of interest related to this study.

**8.8 Dissemination**

We planned to publish the results of this controlled trial to an international journal following peer review.

**Appendix A**: Subintensive Care Unit daily monitoring sheet


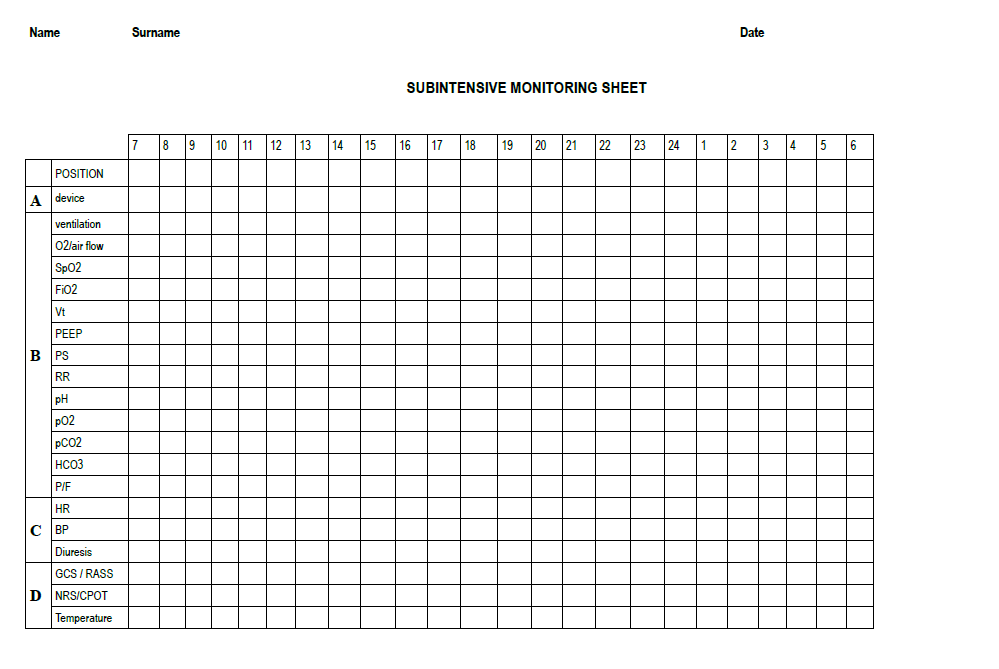

1. Lu R, Zhao X, Li J, Niu P, Yang B, Wu H, et al. Genomic characterisation and epidemiology of 2019 novel coronavirus: implications for virus origins and receptor binding. Lancet 2020;395:565-74 [↑](#endnote-ref-1)
2. Zhou F, Yu T, Du R, et al. Clinical course and risk factors for mortality

   of adult inpatients with COVID-19 in Wuhan, China: a retrospective

   cohort study. Lancet 2020;395:1054–62. [↑](#endnote-ref-2)
3. [Clinical course and predictors of 60-day mortality in 239 critically ill patients with COVID-19: a multicenter retrospective study from Wuhan, China](https://www.ncbi.nlm.nih.gov/pmc/articles/PMC7336107/)

   Jiqian Xu, Xiaobo Yang, Luyu Yang, Xiaojing Zou, Yaxin Wang, Yongran Wu, Ting Zhou, Yin Yuan, Hong Qi, Shouzhi Fu, Hong Liu, Jia’an Xia, Zhengqin Xu, Yuan Yu, Ruiting Li, Yaqi Ouyang, Rui Wang, Lehao Ren, Yingying Hu, Dan Xu, Xin Zhao, Shiying Yuan, Dingyu Zhang, You Shang

   Crit Care. 2020; 24: 394 [↑](#endnote-ref-3)
4. Zheng Jie Lim, Ashwin Subramaniam, Mallikarjuna Ponnapa Reddy, Gabriel Blecher, Umesh Kadam, Afsana Afroz, Baki Billah, Sushma Ashwin, Mark Kubicki, Federico Bilotta, J. Randall Curtis, Francesca Rubulotta[Case Fatality Rates for Patients with COVID-19 Requiring Invasive Mechanical Ventilation. A Meta-analysis](https://www.ncbi.nlm.nih.gov/pmc/articles/PMC7781141/). Am J Respir Crit Care Med. 2021 Jan 1; 203(1): 54–66. [↑](#endnote-ref-4)
5. Gattinoni L, Busana M, Giosa L, Macrì MM, Quintel M [Prone Positioning in Acute Respiratory Distress Syndrome.](https://pubmed.ncbi.nlm.nih.gov/31060091/) Semin Respir Crit Care Med. 2019 ;40:94-100. [↑](#endnote-ref-5)
6. Pelosi P, Tubiolo D, Mascheroni D, et al. Effects of the prone position on respiratory mechanics and gas exchange during acute lung injury. Am J Respir Crit Care Med 1998;157(02):387–393 [↑](#endnote-ref-6)
7. Gattinoni L, D’Andrea L, Pelosi P, Vitale G, Pesenti A, Fumagalli R. “Regional effects and mechanism of positive end‐expiratory pressure in early adult respiratory distress syndrome”. JAMA

   1993;269:2122–2127. [↑](#endnote-ref-7)
8. Lai‐Fook SJ, Rodarte JR. “Pleural pressure distribution and its relationship to lung volume and

   interstitial pressure”. J Appl Physiol 1991;70(3):967. [↑](#endnote-ref-8)
9. Gattinoni L, Pelosi P, Vitale G, Pesenti A, D’Andrea L, Mascheroni D. “Body position changes

   redistribute lung computed‐tomographic density in patients with acute respiratory failure”.

   Anesthesiology 1991;74:15–23. [↑](#endnote-ref-9)
10. RK, Hubmayr RD. “The prone position eliminates compression of the lungs by the heart”. Am J

    Respir Crit Care Med 2000;161:1660–1665. [↑](#endnote-ref-10)
11. Puybasset L, Cluzel P, Chao N, Slutsky AS, Coriat P, Rouby JJ. “A computed tomography scan

    assessment of regional lung volume in acute lung injury. The CT Scan ARDS Study Group”. Am J

    Respir Crit Care Med 1998;158(5 Pt 1):1644. [↑](#endnote-ref-11)
12. Malbouisson LM, Busch CJ, Puybasset L, Lu Q, Cluzel P, Rouby JJ. “Role of the heart in the loss of aeration characterizing lower lobes in acute respiratory distress syndrome. CT Scan ARDS Study

    Group”. Am J Respir Crit Care Med 2000;161(6):2005. [↑](#endnote-ref-12)
13. Malbouisson LM, Busch CJ, Puybasset L, Lu Q, Cluzel P, Rouby JJ. “Role of the heart in the loss of aeration characterizing lower lobes in acute respiratory distress syndrome. CT Scan ARDS Study

    Group”. Am J Respir Crit Care Med 2000;161(6):2005. [↑](#endnote-ref-13)
14. Jozwiak M, Teboul JL, Anguel N, Persichini R, Silva S, Chemla D, Richard C, Monnet X. “Beneficial hemodynamic effects of prone positioning in patients with acute respiratory distress syndrome”. Am J Respir Crit Care Med. 2013;188(12):1428. [↑](#endnote-ref-14)
15. Nyrén S, Radell P, Lindahl SG, Mure M, Petersson J, Larsson SA, Jacobsson H, Sánchez‐Crespo A.“Lung ventilation and perfusion in prone and supine postures with reference to anesthetized and

    mechanically ventilated healthy volunteers”. Anesthesiology. 2010 Mar;112(3):682‐7. [↑](#endnote-ref-15)
16. Guerin C, Reignier J, Richard JC, Beuret P, Gacouin A, Boulain T, et al. Prone positioning in severe acute respiratory distress syndrome. N Engl J Med. 2013;368:2159-68. [↑](#endnote-ref-16)
17. Fan E, Del Sorbo L, Goligher EC, Hodgson CL, Munshi L, Walkey AJ, et al. [An Official American Thoracic Society/European Society of Intensive Care Medicine/Society of Critical Care Medicine Clinical Practice Guideline: Mechanical Ventilation in Adult Patients with Acute Respiratory Distress Syndrome.](https://pubmed.ncbi.nlm.nih.gov/28459336/) Am J Respir Crit Care Med. 2017;195:1253-1263 [↑](#endnote-ref-17)
18. **Fan E**, Brodie D, Slutsky AS. [Acute Respiratory Distress Syndrome: Advances in Diagnosis and Treatment.](https://pubmed.ncbi.nlm.nih.gov/29466596/) JAMA. 2018;319: 698-710. [↑](#endnote-ref-18)
19. ### Srinivas Murthy, MD, CM, MHSc; Charles D. Gomersall, MBBS; Robert A. Fowler, MD, CM, MSc. Care for Critically Ill patients With COVID-19. JAMA. 2020; 323(15):1499-1500.

    [↑](#endnote-ref-19)
20. Jason T. Poston, MD; Bhakti K. Patel, MD; Andrew M. Davis, MD, MPH. May 12, 2020 [Management of Critically Ill Adults with COVID-19.](https://jamanetwork.com/journals/jama/fullarticle/2763879?resultClick=1) JAMA. 2020; 323(18):1839-1841. doi: 10.1001/jama.2020.4914 [↑](#endnote-ref-20)
21. Rosanna Vaschetto, Francesco Barone-Adesi, Fabrizio Racca, Claudio Pissaia, Carlo Maestrone, Davide Colombo, Carlo Olivieri, Nello De Vita, Erminio Santangelo, Lorenza Scotti, Luigi Castello, Tiziana Cena, Martina Taverna, Luca Grillenzoni, Maria Adele Moschella, Gianluca Airoldi, Silvio Borrè, Francesco Mojoli, Francesco Della Corte, Paolo Navalesi, Gianmaria Cammarota, Marta Baggiani, Sara Baino, Piero Balbo, Simona Bazzano, Valeria Bonato, Sara Carbonati, Federico Crimaldi, Veronica Daffara, Luca De Col, Matteo Maestrone, Mario Malerba, Federica Moroni, Raffaella Perucca, Mario Pirisi, Valentina Rondi, Daniela Rosalba, Letizia Vanni, Francesca Vigone. [Outcomes of COVID-19 patients treated with continuous positive airway pressure outside ICU](https://www.ncbi.nlm.nih.gov/pmc/articles/PMC7607967/). ERJ Open Res. 2020 Oct 30 : 00541-2020. doi: 10.1183/23120541.00541-2020 [Epub ahead of print] [↑](#endnote-ref-21)
22. Scaravilli V, Grasselli G, Castagna L, Zanella A, Isgrò S, Lucchini A, Patroniti N, Bellani G, Pesenti A. Prone positioning improves oxygenation in spontaneously breathing nonintubated patients with hypoxemic acute respiratory failure: A retrospective study. J Crit Care. 2015 ;30:1390-4.. [↑](#endnote-ref-22)
23. Ding L, Wang L, Ma W, He H. Efficacy and safety of early prone positioning combined with

    HFNO or NIV in moderate to severe ARDS: a multi-center prospective cohort study. Crit

    Care. 2020;24:28. [↑](#endnote-ref-23)
24. Weatherald J, Solverson K, Zuege DJ, Loroff N, Fiest KM, Parhar KKS. [Awake prone positioning for COVID-19 hypoxemic respiratory failure: A rapid review](https://www.ncbi.nlm.nih.gov/pmc/articles/PMC7450241/) J Crit Care. 2021; 61: 63–70 [↑](#endnote-ref-24)
25. Anna Coppo, Giacomo Bellani, Dario Winterton, Michela Di Pierro, Alessandro Soria, Paola Faverio, Matteo Cairo, Silvia Mori, Grazia Messinesi, Ernesto Contro, Paolo Bonfanti, Annalisa Benini, Maria Grazia Valsecchi, Laura Antolini, Giuseppe Foti. [Feasibility and physiological effects of prone positioning in non-intubated patients with acute respiratory failure due to COVID-19 (PRON-COVID): a prospective cohort study](https://www.ncbi.nlm.nih.gov/pmc/articles/PMC7304954/). Lancet Respir Med. 2020 Aug; 8(8): 765–774 [↑](#endnote-ref-25)
26. Ponnapa Reddy M, Subramaniam A, Afroz A, et al. Prone Positioning of Nonintubated Patients With Coronavirus Disease 2019-A Systematic Review and Meta-Analysis. Critical Care Medicine. 2021 Apr. DOI: 10.1097/ccm.0000000000005086. [↑](#endnote-ref-26)
27. Coudroy R, Frat J-P, Girault C, Thille AW: Reliability of methods to estimate the fraction of inspired oxygen in patients with acute respiratory failure breathing through non-rebreather reservoir bag oxygen mask. Thorax 2020, doi:10.1136/thoraxjnl-2020-214863. [↑](#endnote-ref-27)
28. Antonelli M, Conti G, Rocco M, Bufi M, De Blasi RA, Vivino G, Gasparetto A, Meduri GU: A comparison of noninvasive positive-pressure ventilation and conventional mechanical ventilation in patients with acute respiratory failure. N Engl J Med 1998, 339:429–35. [↑](#endnote-ref-28)
29. Patel BK, Wolfe KS, Pohlman AS, Hall JB, Kress JP: Effect of Noninvasive Ventilation Delivered by Helmet vs Face Mask on the Rate of Endotracheal Intubation in Patients With Acute Respiratory Distress Syndrome: A Randomized Clinical Trial. JAMA 2016, 635 315:2435–41. [↑](#endnote-ref-29)
30. Stilma W, Åkerman E, Artigas A, Bentley A, Bos LD, Bosman TJC, de Bruin H, Brummaier T, Buiteman-Kruizinga LA, Carcò F, Chesney G, Chu C, Dark P, Dondorp AM, Gijsbers HJH, Gilder ME, Grieco DL, Inglis R, Laffey JG, Landoni G, Lu W, Maduro LMN, McGready R, McNicholas B, de Mendoza D, Morales-Quinteros L, Nosten F, Papali A, Paternoster G, Paulus F, Pisani L, Prud'homme E, Ricard JD, Roca O, Sartini C, Scaravilli V, Schultz MJ, Sivakorn C, Spronk PE, Sztajnbok J, Trigui Y, Vollman KM, van der Woude MCE. Awake Proning as an Adjunctive Therapy for Refractory Hypoxemia in Non-Intubated Patients with COVID-19 Acute Respiratory Failure: Guidance from an International Group of Healthcare Workers. Am J Trop Med Hyg. 2021 ;104:1676–86. [↑](#endnote-ref-30)
31. Knight SR, Ho A, Pius R, et al. Risk stratification of patients admitted to hospital with covid-19 using the ISARIC WHO Clinical Characterisation Protocol: development and validation of the 4C Mortality Score. BMJ 2020; 370: m3339. [↑](#endnote-ref-31)
32. Patient-ventilator asynchrony during noninvasive ventilation: the role of expiratory trigger.

    Calderini E, Confalonieri M, Puccio PG, Francavilla N, Stella L, Gregoretti C.

    Intensive Care Med. 1999 Jul;25(7):662-7 [↑](#endnote-ref-32)
33. Mojoli F, Iotti G a, Currò I, Pozzi M, Via G, Venti A, Braschi A: An optimized set-up for 654 helmet noninvasive ventilation improves pressure support delivery and patient-655 ventilator interaction. Intensive Care Med 2013, 39:38–44. [↑](#endnote-ref-33)
34. Taccone P, Hess D, Caironi P, Bigatello LM: Continuous positive airway pressure delivered with a “helmet”: effects on carbon dioxide rebreathing. Crit Care Med 2004, 661 32:2090–2096. [↑](#endnote-ref-34)
35. Antonelli M, Conti G, Pelosi P, Gregoretti C, Pennisi MA, Costa R, Severgnini P, Chiaranda M, Proietti R. New treatment of acute hypoxemic respiratory failure: noninvasive pressure support ventilation delivered by helmet--a pilot controlled trial. Crit Care Med. 2002 Mar;30(3):602-8. [↑](#endnote-ref-35)
36. Hilbert G, Navalesi P, Girault C. Is sedation safe and beneficial in patients receiving NIV?Yes. Int Care Med 2015; 41: 1688-1691 [↑](#endnote-ref-36)
37. John W. Devlin , PharmD ; Nada S. Al-Qadheeb , PharmD, FCCP ; Amy Chi , MD ;

    Russel J. Roberts , PharmD ; Imrana Qawi , MD ; Erik Garpestad , MD , FCCP ;

    and Nicholas S. Hill , MD , FCCP Efficacy and Safety of Early Dexmedetomidine

    During Noninvasive Ventilation for Patients

    With Acute Respiratory Failure. CHEST 2014; 145(6):1204 –1212 [↑](#endnote-ref-37)
38. Baptiste Deletombe a, Thibaut Trouve-Buisson a, Alexandre Godon a, Dominique Falcon a, Lise Giorgis-Allemand b,c, Pierre Bouzat a,d,e, Jean-Luc Bosson b,c,f, Jean-Francois Payen Dexmedetomidine to facilitate non-invasive ventilation after blunt chest trauma: A randomised, double-blind, crossover, placebo-controlled pilot study. Anaesth Crit Care Pain Med 38 (2019) 477–483 [↑](#endnote-ref-38)
39. Longrois et al.: Sedation in non-invasive ventilation:

    do we know what to do (and why)? Multidisciplinary Respiratory Medicine 2014 9:56. [↑](#endnote-ref-39)
40. Zhao Huang, Yu-sheng Chen, Zi-li Yang and Ji-yun Liu Dexmedetomidine Versus Midazolam for the Sedation of Patients with Non-invasive Ventilation Failure Intern Med 51: 2299-2305, 2012) [↑](#endnote-ref-40)
41. Muriel A, Penuelas O, Frutos-Vivar F, et al. Impact of sedation and analgesia during noninvasive positive pressure ventilation on outcome: a marginal structural model causal analysis. Int Care Med 2015; 41: 1586-1600 [↑](#endnote-ref-41)
42. Hao, G. W., Luo, J. C., Xue, Y., Ma, G. G., Su, Y., Hou, J. Y., Yu, S. J., Liu, K., Zheng, J. L., Tu, G. W., & Luo, Z. (2020). Remifentanil versus dexmedetomidine for treatment of cardiac surgery patients with moderate to severe noninvasive ventilation intolerance (REDNIVIN): a prospective, cohort study. Journal of thoracic disease, 12(10), 5857–5868. https://doi.org/10.21037/jtd-20-1678 [↑](#endnote-ref-42)
43. Barazzoni R, Bischoff SC, Breda J, Wickramasinghe K, Krznaric Z, Nitzan D, Pirlich M, Singer P; endorsed by the ESPEN Council. ESPEN expert statements and practical guidance for nutritional management of individuals with SARS-CoV-2 infection. Clin Nutr. 2020 ;39:1631-1638. [↑](#endnote-ref-43)
44. Sinha P, Fauvel NJ, Singh P, Soni N. Analysis of ventilatory ratio as a novel method to monitor ventilatory adequacy at the bedside. Crit Care. 2013 Feb 27;17(1):R34. doi: 10.1186/cc12541. PMID: 23445563; PMCID: PMC4057449. [↑](#endnote-ref-44)
45. Sinha P, Calfee CS, Beitler JR, et al. Physiologic analysis and clinical performance of the ventilatory ratio in acute respiratory distress syndrome. Am J Respir Crit Care Med 2019; 199: 333–41. [↑](#endnote-ref-45)
46. Grasselli G, Tonetti T, Protti A, Langer T, Girardis M, Bellani G, Laffey J, Carrafiello G, Carsana L, Rizzuto C, Zanella A, Scaravilli V, Pizzilli G, Grieco DL, Di Meglio L, de Pascale G, Lanza E, Monteduro F, Zompatori M, Filippini C, Locatelli F, Cecconi M, Fumagalli R, Nava S, Vincent JL, Antonelli M, Slutsky AS, Pesenti A, Ranieri VM; collaborators. Pathophysiology of COVID-19-associated acute respiratory distress syndrome: a multicentre prospective observational study. Lancet Respir Med. 2020;8:1201-1208Fine modulo [↑](#endnote-ref-46)
47. Liu X, Liu X, Xu Y, Xu Z, Huang Y, Chen S, Li S, Liu D, Lin Z, Li Y. Ventilatory Ratio in Hypercapnic Mechanically Ventilated Patients with COVID-19-associated Acute Respiratory Distress Syndrome. Am J Respir Crit Care Med. 2020 May 15;201(10):1297-1299. doi: 10.1164/rccm.202002-0373LE. PMID: 32203672; PMCID: PMC7233337. [↑](#endnote-ref-47)
48. Fusina F, Albani F, Bertelli M, Cavallo E, Crisci S, Caserta R, Nguyen M, Grazioli M, Schivalocchi V, Rosano A, Natalini G. Corrected Minute Ventilation Is Associated With Mortality in ARDS Caused by COVID-19. Respir Care. 2021;66:619-625. [↑](#endnote-ref-48)
49. Fodil R, Lellouche F, Mancebo J, Sbirlea-Apiou G, Isabey D, Brochard L, Louis B. Comparison of patient-ventilator interfaces based on their computerized effective dead space. Intensive Care Med. 2011 ;37:257-62. doi: 10.1007/s00134-010-2066-3. [↑](#endnote-ref-49)
50. Nava S, Navalesi P, Gregoretti C. Interfaces and Humidification for Noninvasive Mechanical Ventilation. Respir Care 2009;54(1):71– 82. [↑](#endnote-ref-50)
51. Arbelot C, Neto FLD, Gao Y et al. Lung ultrasound in emergency and critically Ill patientsnumber of supervised exams to reach basic competence. Anesthesiology 2020; 132:899–907. [↑](#endnote-ref-51)
52. Dargent, A., Chatelain, E., Kreitmann, L., Quenot, J. P., Cour, M., Argaud, L., & COVID-LUS study group. Lung ultrasound score to monitor COVID-19 pneumonia progression in patients with ARDS. PloS one, 2020; 15: e0236312. [↑](#endnote-ref-52)
53. Lichter, Y., Topilsky, Y., Taieb, P., Banai, A., Hochstadt, A., Merdler, I., Gal Oz, A., Vine, J., Goren, O., Cohen, B., Sapir, O., Granot, Y., Mann, T., Friedman, S., Angel, Y., Adi, N., Laufer-Perl, M., Ingbir, M., Arbel, Y., Matot, I., … Szekely, Y. . Lung ultrasound predicts clinical course and outcomes in COVID-19 patients. Intensive care medicine, 2020; 46: 1873–83. [↑](#endnote-ref-53)
54. Kirkpatrick JN, Mitchell C, Taub C, Kort S, Hung J, Swaminathan M (2020). ASE statement on protection of patients and echocardiography service providers during the 2019 novel coronavirus outbreak. J Am Coll Cardiol. https ://doi.org/10.1016/j.echo.2020.04.001 [↑](#endnote-ref-54)
55. Ji L, Cao C, Gao Y, Zhang W, Xie Y, Duan Y, Kong S, You M, Ma R, Jiang L, Liu J, Sun Z, Zhang Z, Wang J, Yang Y, Lv Q, Zhang L, Li Y, Zhang J, Xie M. Prognostic value of bedside lung ultrasound score in patients with COVID-19. Crit Care. 2020 22;24: 700.. [↑](#endnote-ref-55)
56. Kong S, Wang J, Li Y, Tian Y, Yu C, Zhang D, Li H, Zhang L, Pang X, Xie M. Value of Bedside Lung Ultrasound in Severe and Critical COVID-19 Pneumonia. Respir Care. 2021;66: 920-927. [↑](#endnote-ref-56)
57. Zieleskiewicz L, Markarian T, Lopez A, Taguet C, Mohammedi N, Boucekine M, Baumstarck K, Besch G, Mathon G, Duclos G, Bouvet L, Michelet P, Allaouchiche B, Chaumoître K, Di Bisceglie M, Leone M; AZUREA Network. Comparative study of lung ultrasound and chest computed tomography scan in the assessment of severity of confirmed COVID-19 pneumonia. Intensive Care Med. 2020;46:1707-1713. [↑](#endnote-ref-57)
58. Salaffi F, Carotti M, Tardella M, Borgheresi A, Agostini A, Minorati D, Marotto D, Di Carlo M, Galli M, Giovagnoni A, Sarzi-Puttini P. The role of a chest computed tomography severity score in coronavirus disease 2019 pneumonia. Medicine (Baltimore). 2020;99:e22433. [↑](#endnote-ref-58)
59. Bouhemad B, Brisson H, Le-Guen M, Arbelot C, Lu Q, Rouby JJ. Bedside ultrasound assessment of positive end-expiratory pressure-induced lung recruitment. Am J Respir Crit Care Med 2011;183:341–347. [↑](#endnote-ref-59)
60. Chiumello D, Mongodi S, Algieri I, Vergani GL, Orlando A, Via G, et al. Assessment of lung aeration and recruitment by CT scan and ultrasound in acute respiratory distress syndrome patients. Crit Care Med 2018;46:1761–1768. [↑](#endnote-ref-60)
61. Stevic N,Chatelain E, Dargent A, Argaud L, Cour M, Guérin C

    [Lung Recruitability Evaluated by Recruitment-to-Inflation Ratio and Lung Ultrasound in COVID-19 Acute Respiratory Distress Syndrome](https://www.ncbi.nlm.nih.gov/pmc/articles/PMC8048756/) Am J Respir Crit Care Med. 2021; 203: 1025–1027. [↑](#endnote-ref-61)
62. [ICNARC – Intensive Care National Audit & Research Centre](https://www.icnarc.org/). Last searched on Jan 20th, 2021 [↑](#endnote-ref-62)
63. Bellani G, Grasselli G, Cecconi M, Antolini L, Borelli M, De Giacomi F, Bosio G, Latronico N, Filippini M, Gemma M, Giannotti C, Antonini B, Petrucci N, Zerbi SM, Maniglia P, Castelli GP, Marino G, Subert M, Citerio G, Radrizzani D, Mediani TS, Lorini FL, Russo FM, Faletti A, Beindorf A, Covello RD, Greco S, Bizzarri MM, Ristagno G, Mojoli F, Pradella A, Severgnini P, Da Macallè M, Albertin A, Ranieri VM, Rezoagli E, Vitale G, Magliocca A, Cappelleri G, Docci M, Aliberti S, Serra F, Rossi E, Valsecchi MG, Pesenti A, Foti G; COVID-19 Lombardy ICU Network. Noninvasive Ventilatory Support of COVID-19 Patients Outside the Intensive Care Units (WARd-COVID). Ann Am Thorac Soc. 2021 Jan 4. doi: 10.1513/AnnalsATS.202008-1080OC. Epub ahead of print. PMID: 33395553. [↑](#endnote-ref-63)
64. Grieco DL, Menga LS, Cesarano M, et al. Effect of Helmet Noninvasive Ventilation vs High-Flow Nasal Oxygen on Days Free of Respiratory Support in Patients With COVID-19 and Moderate to Severe Hypoxemic Respiratory Failure: The HENIVOT Randomized Clinical Trial. JAMA. Published online March 25, 2021. doi:10.1001/jama.2021.4682 [↑](#endnote-ref-64)
65. [Outcomes of COVID-19 patients treated with continuous positive airway pressure outside ICU](https://www.ncbi.nlm.nih.gov/pmc/articles/PMC7607967/)

    Rosanna Vaschetto, Francesco Barone-Adesi, Fabrizio Racca, Claudio Pissaia, Carlo Maestrone, Davide Colombo, Carlo Olivieri, Nello De Vita, Erminio Santangelo, Lorenza Scotti, Luigi Castello, Tiziana Cena, Martina Taverna, Luca Grillenzoni, Maria Adele Moschella, Gianluca Airoldi, Silvio Borrè, Francesco Mojoli, Francesco Della Corte, Paolo Navalesi, Gianmaria Cammarota, Marta Baggiani, Sara Baino, Piero Balbo, Simona Bazzano, Valeria Bonato, Sara Carbonati, Federico Crimaldi, Veronica Daffara, Luca De Col, Matteo Maestrone, Mario Malerba, Federica Moroni, Raffaella Perucca, Mario Pirisi, Valentina Rondi, Daniela Rosalba, Letizia Vanni, Francesca Vigone

    ERJ Open Res. 2020 Oct 30 : 00541-2020. doi: 10.1183/23120541.00541-2020 [Epub ahead of print] [↑](#endnote-ref-65)
66. Padrão EMH, Valente FS, Besen BAMP, Rahhal H, Mesquita PS, de Alencar JCG, da Costa MGP, Wanderley APB, Emerenciano DL, Bortoleto FM, Fortes JCL, Marques B, de Souza SFB, Marchini JFM, Neto RAB, de Souza HP; COVIDTEAM. Awake Prone Positioning in COVID-19 Hypoxemic Respiratory Failure: Exploratory Findings in a Single-center Retrospective Cohort Study. Acad Emerg Med. 2020 Dec;27(12):1249-1259. doi: 10.1111/acem.14160. Epub 2020 Nov 21. PMID: 33107664. [↑](#endnote-ref-66)
67. Sophie Alviset, Quentin Riller, Jérôme Aboab, Kelly Dilworth, Pierre-Antoine Billy, Yannis Lombardi, Mathilde Azzi, Luis Ferreira Vargas, Laurent Laine, Mathilde Lermuzeaux, Nathalie Mémain, Daniel Silva, Tona Tchoubou, Daria Ushmorova, Hanane Dabbagh, Simon Escoda, Rémi Lefrançois, Annelyse Nardi, Armand Ngima, Vincent Ioos[Continuous Positive Airway Pressure (CPAP) face-mask ventilation is an easy and cheap option to manage a massive influx of patients presenting acute respiratory failure during the SARS-CoV-2 outbreak: A retrospective cohort study](https://www.ncbi.nlm.nih.gov/pmc/articles/PMC7556440/). PLoS One. 2020; 15: e0240645 [↑](#endnote-ref-67)
68. Rob J Hallifax, Benedict ML Porter, Patrick JD Elder, Sarah B Evans, Chris D Turnbull, Gareth Hynes, Rachel Lardner, Kirsty Archer, Henry V Bettinson, Annabel H Nickol, William G Flight, Stephen J Chapman, Maxine Hardinge, Rachel K Hoyles, Peter Saunders, Anny Sykes, John M Wrightson, Alastair Moore, Ling-Pei Ho, Emily Fraser, Ian D Pavord, Nicholas P Talbot, Mona Bafadhel, Nayia Petousi, Najib M Rahman, On behalf of Oxford Respiratory Group. [Successful awake proning is associated with improved clinical outcomes in patients with COVID-19: single-centre high-dependency unit experience](https://www.ncbi.nlm.nih.gov/pmc/articles/PMC7490910/). BMJ Open Respir Res. 2020; 7(1): e000678 [↑](#endnote-ref-68)
69. Jiqian Xu, Xiaobo Yang, Luyu Yang, Xiaojing Zou, Yaxin Wang, Yongran Wu, Ting Zhou, Yin Yuan, Hong Qi, Shouzhi Fu, Hong Liu, Jia’an Xia, Zhengqin Xu, Yuan Yu, Ruiting Li, Yaqi Ouyang, Rui Wang, Lehao Ren, Yingying Hu, Dan Xu, Xin Zhao, Shiying Yuan, Dingyu Zhang, You Shang [Clinical course and predictors of 60-day mortality in 239 critically ill patients with COVID-19: a multicenter retrospective study from Wuhan, China](https://www.ncbi.nlm.nih.gov/pmc/articles/PMC7336107/). Crit Care. 2020; 24: 394 [↑](#endnote-ref-69)
70. Rassen JA, Shelat AA, Myers J, Glynn RJ, Rothman KJ, Schneeweiss S. One-to-many propensity score matching in cohort studies. Pharmacoepidemiol Drug Saf. 2012;21 S2:69-80. [↑](#endnote-ref-70)
71. Peduzzi P, Concato J, Feinstein AR, Holford TR (1995) Importance of events per independent variable in proportional hazards regression analysis. II. Accuracy and precision of regression estimates. Journal of Clinical Epidemiology 48:1503-1510.  [↑](#endnote-ref-71)
72. Long JS (1997) Regression Models for categorical and limited dependent variables. Thousand Oaks, CA: Sage Publications.  [↑](#endnote-ref-72)
73. Langer T, Brioni M, Guzzardella A, Carlesso E, Cabrini L, Castelli G, Dalla Corte F, De Robertis E, Favarato M, Forastieri A, Forlini C, Girardis M, Grieco DL, Mirabella L, Noseda V, Previtali P, Protti A, Rona R, Tardini F, Tonetti T, Zannoni F, Antonelli M, Foti G, Ranieri M, Pesenti A, Fumagalli R, Grasselli G; PRONA-COVID Group. Prone position in intubated, mechanically ventilated patients with COVID-19: a multi-centric study of more than 1000 patients. Crit Care. 2021;25:128 [↑](#endnote-ref-73)
74. Matthews JN, Altman DG, Campbell MJ, Royston P. Analysis of serial measurements in medical research. BMJ. 1990 Jan 27;300(6719):230-5. [↑](#endnote-ref-74)
75. Trevor A. Craney & James G. Surles (2002) Model-Dependent Variance Inflation Factor Cutoff Values, Quality Engineering, 14:3, 391-403, DOI: [10.1081/QEN-120001878](https://doi.org/10.1081/QEN-120001878) [↑](#endnote-ref-75)
76. Langer T, Brioni M, Guzzardella A, Carlesso E, Cabrini L, Castelli G, Dalla Corte F, De Robertis E, Favarato M, Forastieri A, Forlini C, Girardis M, Grieco DL, Mirabella L, Noseda V, Previtali P, Protti A, Rona R, Tardini F, Tonetti T, Zannoni F, Antonelli M, Foti G, Ranieri M, Pesenti A, Fumagalli R, Grasselli G; PRONA-COVID Group. Prone position in intubated, mechanically ventilated patients with COVID-19: a multi-centric study of more than 1000 patients. Crit Care. 2021;25:128 [↑](#endnote-ref-76)
77. Dignam JJ, Zhang Q, Kocherginsky M. The use and interpretation of competing risks regression models. Clin Cancer Res. 2012;18:2301-2308. [↑](#endnote-ref-77)
